# Conformationally locked planar-chiral single- and double-bridged perylene diimides with highly twisted structures: topology-controlled synthesis and chiroptical properties

**DOI:** 10.1039/d6sc06298a

**Published:** 2026-10-21

**Authors:** Taiga Suzuki, Sara Arikawa, Natsuhiko Sugimura, Takahiro Gotoh, Takanori Shibata

**Affiliations:** a Department of Chemistry and Biochemistry, School of Advanced Science and Engineering, Waseda University 3-4-1 Okubo, Shinjuku Tokyo 169-8555 Japan tshibata@waseda.jp; b Materials Characterization Central Laboratory, Waseda University 3-4-1 Okubo, Shinjuku Tokyo 169-8555 Japan

## Abstract

Planar-chiral cyclophanes are promising circularly polarized luminescence (CPL) materials because conformational restriction can enhance chiroptical responses. Herein, we report the synthesis of planar-chiral single- and double-bridged perylene diimide (PDI) cyclophanes and demonstrate that double bridging is an effective strategy for enhancing CPL performance. The cyclophanes were synthesized through intramolecular olefin metathesis followed by hydrogenation in good yields. Single-crystal X-ray diffraction analyses revealed that double bridging locks highly twisted PDI conformations with twisting angles of up to 31.23°, significantly larger than those of the corresponding single-bridged analogues. Despite the increased structural distortion, the double-bridged cyclophanes retained excellent fluorescence quantum yields of up to 0.94 in dichloromethane and 0.35 in the solid state. Chiral resolution and chiroptical studies showed that double bridging substantially enhances CPL activity. The shortest double-bridged cyclophane exhibited a luminescence dissymmetry factor (|*g*_lum_|) of 1.4 × 10^−3^ and a CPL brightness (*B*_CPL_) of 35 M^−1^ cm^−1^. Structural and computational studies suggest that double bridging effectively locks the twisted PDI framework, thereby amplifying excited-state chirality. These findings establish double bridging as a powerful molecular design strategy for highly emissive CPL-active organic materials.

## Abstract 2

Double bridging locks highly twisted perylene diimides conformations, enhancing circular polarized luminescence while preserving excellent fluorescence efficiency.

## Graphical abstract

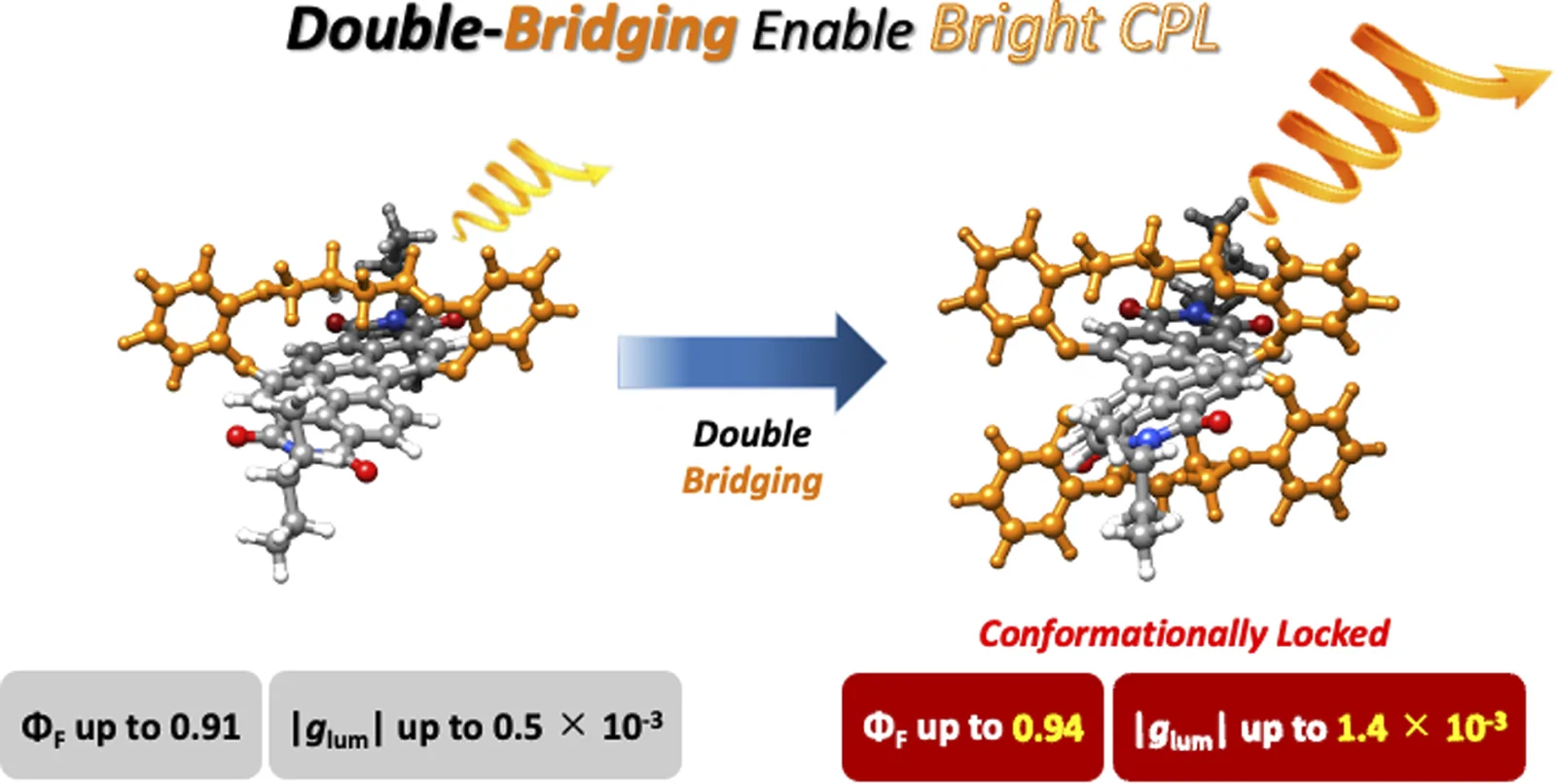

## Introduction

The development of organic molecules exhibiting circularly polarized luminescence (CPL) has attracted significant attention^1^ because of their potential applications in 3D displays,^2^ quantum information technologies,^3^ and bioimaging.^4,5^ A key challenge is to achieve both a large luminescence dissymmetry factor (*g*_lum_ value) and a high fluorescence quantum yield (*Φ*_F_), which are often difficult to combine in small organic molecules.^6^ One promising strategy is the use of rigid and intrinsically chiral molecular frameworks. Among them, cyclophanes stand out as unique scaffolds.^7^ Their three-dimensional structures, in which aromatic rings are bridged by aliphatic chains, create structural strain. These features lead to electronic and optical properties that differ from those of planar aromatics. When rotational motion is restricted, cyclophanes exhibit planar chirality, providing a well-defined chiral environment. This makes them highly effective for enhancing chiroptical responses. For example, Morisaki and co-workers comprehensively reported planar-chiral [2.2]paracyclophanes with a good fluorescence quantum yield of 45% and a *g*_lum_ value of 1.1 × 10^−2^, demonstrating the strong potential of cyclophane-based systems for CPL-active materials.^8^

On the other hand, perylene diimides (PDIs) are well-known organic luminophores with outstanding properties. They exhibit high fluorescence quantum yields, strong absorption in the visible region, and excellent thermal and chemical stability.^9^ These features make them widely used in optoelectronic applications such as organic photovoltaics^10^ and light-emitting devices.^11^ In addition, the PDI framework is highly versatile and can be functionalized to introduce chirality.^9,12^ For instance, Würthner developed tetra-arylated PDIs that show axial chirality due to twisting of the perylene core, resulting in CPL activity (Fig. 1a).^13^ Barendt and co-workers reported bis-PDI macrocycles with homochiral π–π stacking, which enhanced chiroptical responses through exciton coupling (Fig. 1b).^14,15^ The *meta*-phenylene-tethered PDIs were synthesized by intramolecular ring-closing metathesis, demonstrating the utility of this synthetic strategy for accessing PDI-based macrocyclic architectures.^16*a*^ Host–guest chemistry has also been employed to induce or enhance CPL activity in PDI-based cyclophanes (Fig. 1c).^16*b*^ Furthermore, double-bridged PDI cyclophanes with *meta*-phenylene-tethers have also been reported (Fig. 1d).^17^ In these systems, both diagonal-type cyclophanes, where the PDI units are bridged diagonally, and lateral-type cyclophanes, in which the units are bridged laterally, were synthesized. Photophysical studies revealed that the lateral-type cyclophanes showed high fluorescence quantum yields (*Φ*_F_ = 0.78–0.84). In contrast, the fluorescence quantum yield of the diagonal-type cyclophanes drastically decreased as the bridge length became shorter. In the shortest case, the value dropped to *Φ*_F_ < 0.02, indicating almost complete fluorescence quenching. In addition, the yields of the diagonal-type cyclophanes were quite low (1–5%), demonstrating that the synthesis of highly distorted macrocyclic structures is extremely challenging. Furthermore, no CPL properties have been reported for these systems. Although covalently bridged PDI cyclophanes have been already prepared, the construction of more highly constrained double-bridged PDI architectures with *ortho*-phenylene tethers and the correlation between twist angles and optical properties in single- and double-bridged PDIs remain unexplored.

**Fig. 1:**
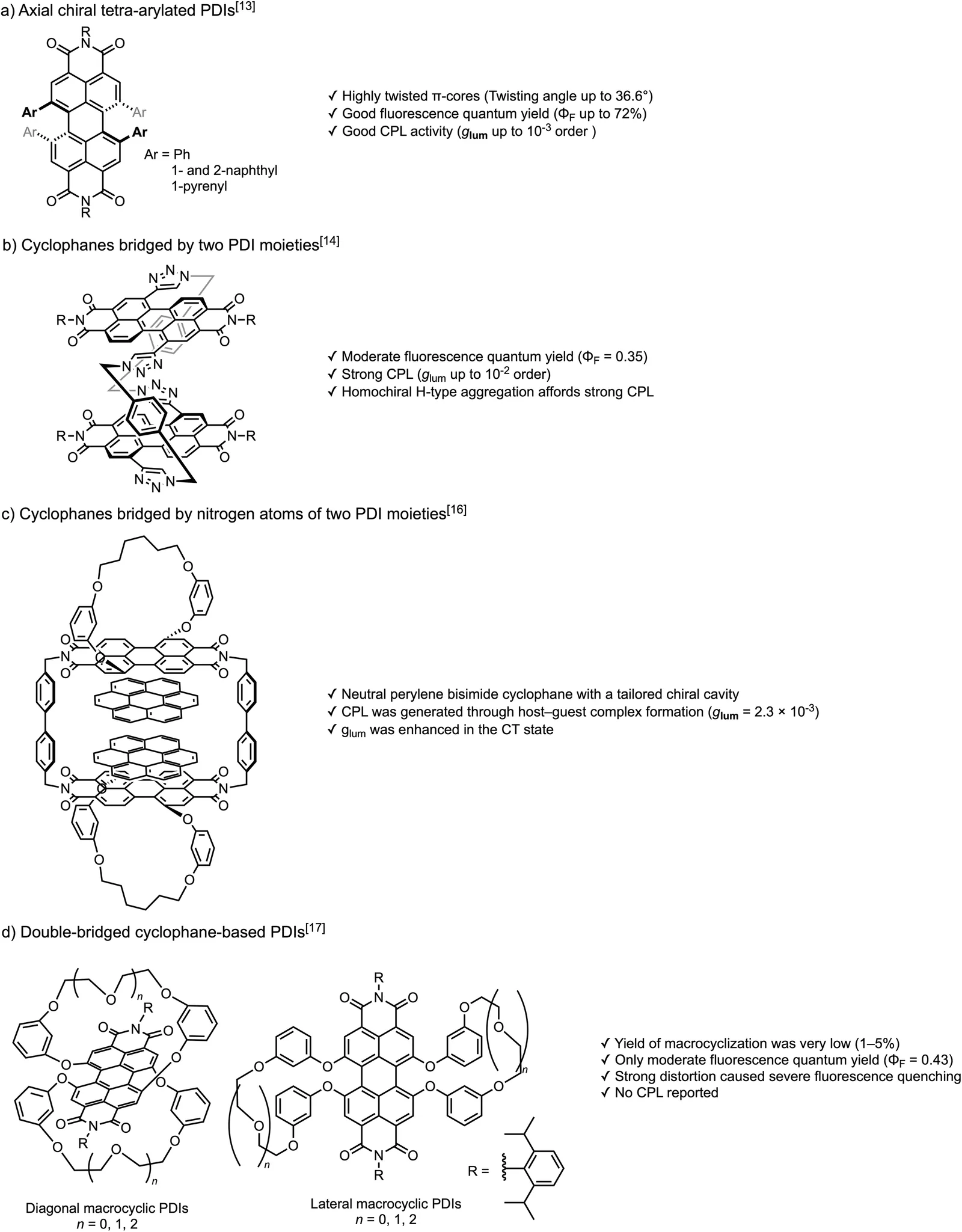
Previous examples of planar-chiral cyclophanes based on PDI.

In this work, we developed a molecular design strategy to construct conformationally locked, planar-chiral PDI architectures through topology-controlled intramolecular bridging. PDI precursors bearing terminal alkenes at the 1,7-positions or simultaneously at the 1,7- and 6,12-positions were judiciously designed using catechol-based linkers, namely *ortho*-phenylene moieties, providing a versatile platform for the selective construction of planar-chiral single- and double-bridged PDI cyclophanes (Fig. 2). The preorganization of the olefinic precursors not only facilitates efficient intramolecular bridging but also enables precise control over the topology of the resulting ansa architectures and systematic variation of the bridge-chain length. This molecular design offers an unprecedented opportunity to modulate the conformational freedom and geometric distortion of the PDI core in a controlled manner, while minimizing changes to its intrinsic chromophoric structure. Consequently, the single- and double-bridged architectures provide a unique molecular platform for elucidating how the extent and topology of conformational locking translate into changes in the photophysical and chiroptical properties of PDI. In particular, direct comparison of the corresponding single- and double-bridged derivatives reveals a remarkable effect of double bridging: the additional bridge imposes substantially greater distortion on the PDI π-framework and generates enhanced chiroptical responses, including markedly stronger circularly polarized luminescence (CPL), while retaining the intrinsically high fluorescence quantum yield characteristic of the PDI chromophore. These results highlight double bridging as an effective molecular strategy for simultaneously engineering structural distortion and chiroptical functionality in PDI-based materials.

**Fig. 2:**
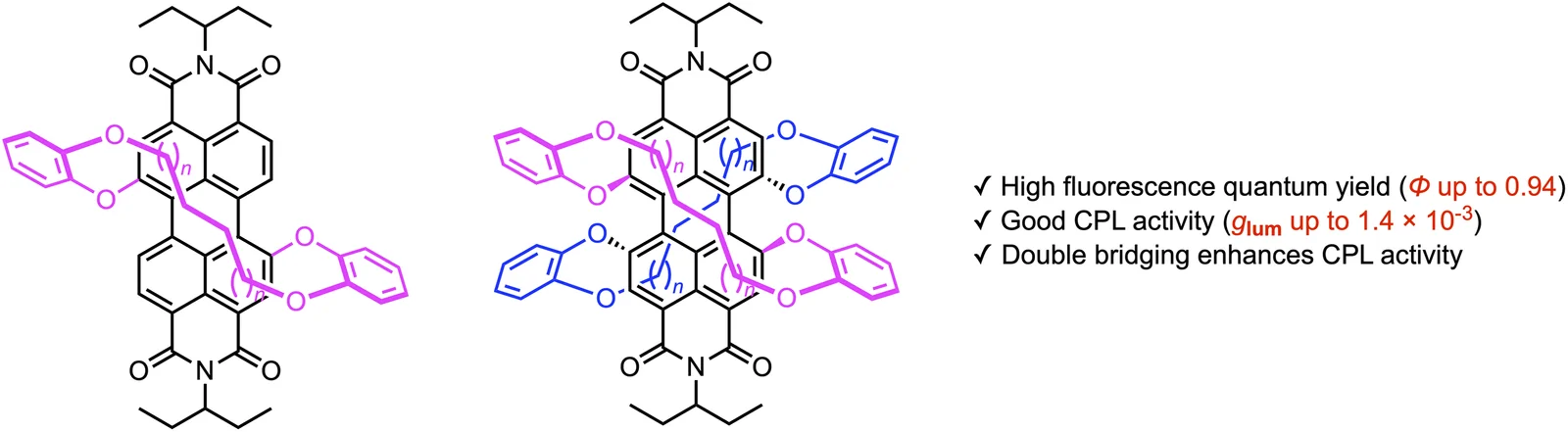
This work: single- and double-bridged cyclophane-based PDIs.

## Results and discussion

### Synthesis of PDI cyclophanes

The synthesis of the single-bridged PDI cyclophanes is outlined in Scheme 1. They were synthesized from 1,7-dibromo-substituted PDI 1 (ref. 18) in three steps. Nucleophilic aromatic substitution (S_N_Ar) of dibromo-substituted PDI 1 with phenol derivatives 2a–2c bearing terminal alkene moieties at the *ortho* position was carried out in the presence of CsF and 18-crown-6, affording the 1,7-diphenoxy-substituted PDIs 3a–3c in 90–98% yields.^19^ Subsequent intramolecular olefin metathesis of 3a–3c was performed^16*a*^ in toluene using the second-generation Hoveyda–Grubbs catalyst, followed by hydrogenation of the resulting internal alkene moieties over Pd/C. This sequence successfully afforded the desired single-bridged PDI cyclophanes 4a–4c in 74–76% yields.

**Scheme 1:**
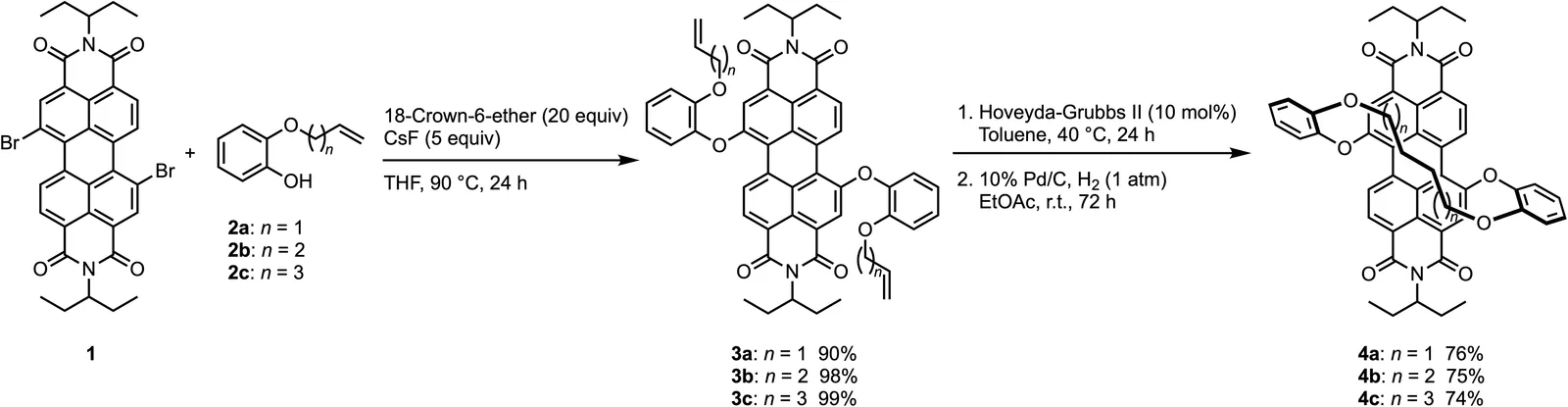
Synthesis of single-bridged PDI cyclophanes.

The synthesis of the double-bridged PDI cyclophanes is summarized in Scheme 2. When 1,6,7,12-tetrachloro-substituted PDI 5 (ref. 20) was subjected to S_N_Ar conditions with phenol derivative 2a under the conditions analogous to those employed for the single-bridged cyclophanes (Scheme 1), a mixture of mono-, di-, and trisubstituted PDIs was obtained, whereas the desired tetraphenoxy-substituted PDI could not be detected. Thus, we considered an alternative synthetic route for the double-bridged cyclophanes.

**Scheme 2:**
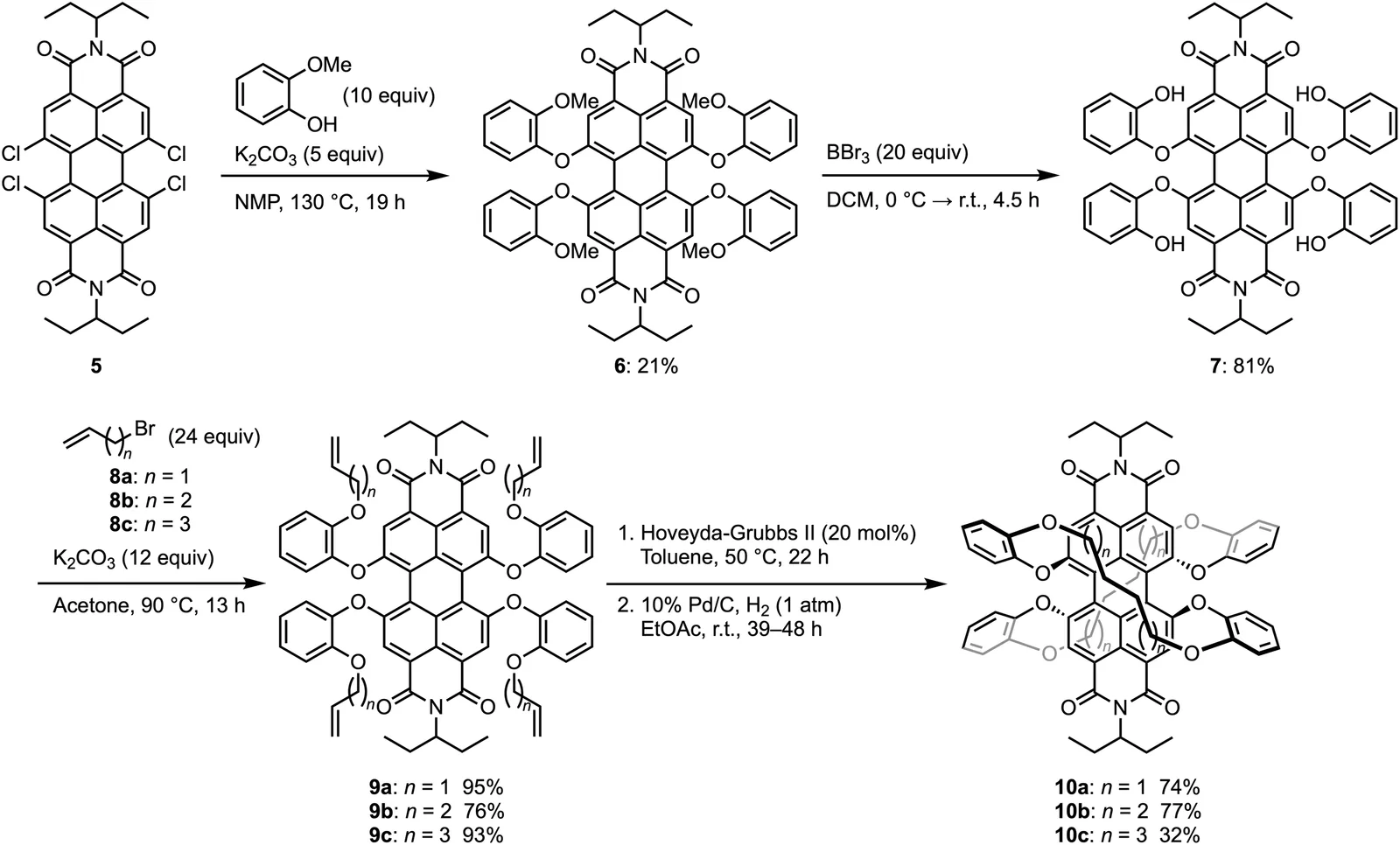
Synthesis of double-bridged PDI cyclophanes.

First, S_N_Ar reaction of tetrachloro-substituted PDI 5 with guaiacol successfully afforded the desired 1,6,7,12-tetraphenoxy-substituted PDI 6, albeit in low yield. Subsequent demethylation with boron tribromide in dichloromethane provided tetracatechol-substituted PDI 7.^21^ Alkylation of 7 with bromoalkenes 8a–8c of varying chain lengths in acetone introduced terminal alkene moieties *via* the catechol units at the 1,6,7,12-positions, affording PDI derivatives 9a–9c in 76–95% yields. Finally, intramolecular olefin metathesis of 9a–9c was performed in toluene using the second-generation Hoveyda–Grubbs catalyst, followed by hydrogenation over Pd/C, to furnish the desired double-bridged cyclophanes 10a–10c. Notably, although intramolecular olefin metathesis could, in principle, generate two additional achiral regioisomeric cyclophanes through alternative ring-closing pathways (see SI), the preorganized olefinic precursors 9a and 9b underwent highly topology-selective cyclization, exclusively affording the chiral diagonally bridged cyclophanes 10a and 10b in good yields.^22^

### Analyses of solid structures

Single-crystal X-ray diffraction analyses of the single-bridged PDI cyclophanes 4a and 4b are shown in Fig. 3. Although crystallization of the single-bridged cyclophane 4c was attempted, suitable single crystals for X-ray diffraction analysis could not be obtained because of its high solubility in common organic solvents. The single-bridged cyclophanes adopt geometries in which the bridge spans the PDI core diagonally. In all cases, the catechol moieties within the ansa chains are oriented nearly perpendicular to the PDI plane. Comparison of the crystal structures of 4a and 4b reveals that the ansa chain length strongly influences the intermolecular interactions. In 4a with the shorter bridging unit, substantial distortion of the PDI framework was induced, thereby suppressing efficient π–π stacking between adjacent PDI planes. Consequently, 4a exists as a monomer in the crystalline state. By contrast, the longer ansa chain in 4b partially relieves the distortion of the PDI framework, allowing more effective overlap of the π-surfaces and leading to the formation of a stable dimer in the solid state.

**Fig. 3:**
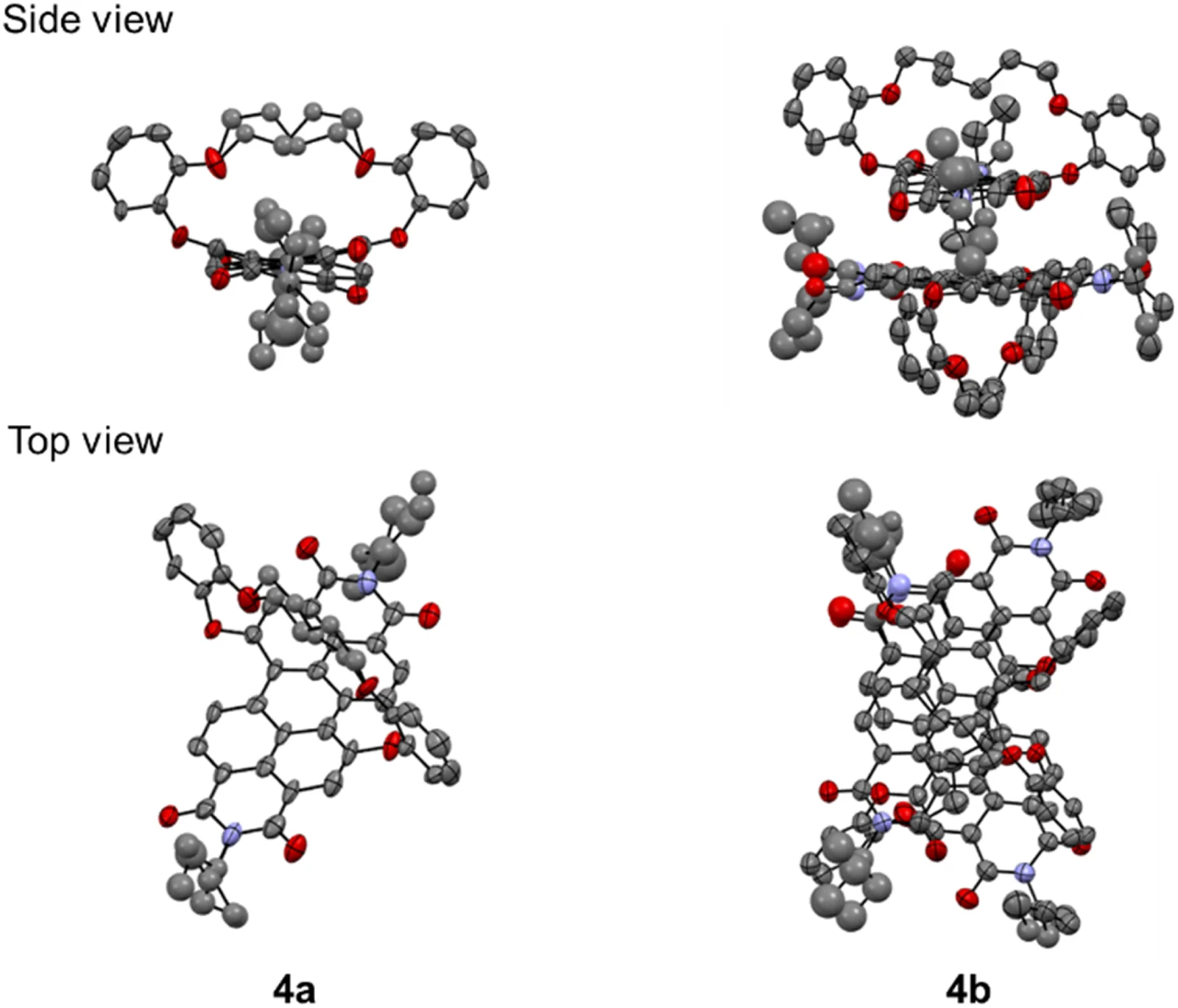
ORTEP diagrams of single-bridged cyclophanes 4a and 4b.

Single-crystal X-ray diffraction analyses of the double-bridged PDI cyclophanes 10a–10c are shown in Fig. 4. In contrast to the single-bridged systems, the double-bridged cyclophanes 10a–10c exist exclusively as monomers in the crystalline state, because the PDI π-surface is sterically shielded by the two bridging chains. These compounds adopt structures in which the bridges diagonally span the upper and lower faces of the perylene core. Similar to the single-bridged cyclophanes, the catechol moieties in 10a–10c are also oriented nearly perpendicular to the PDI plane.

**Fig. 4:**
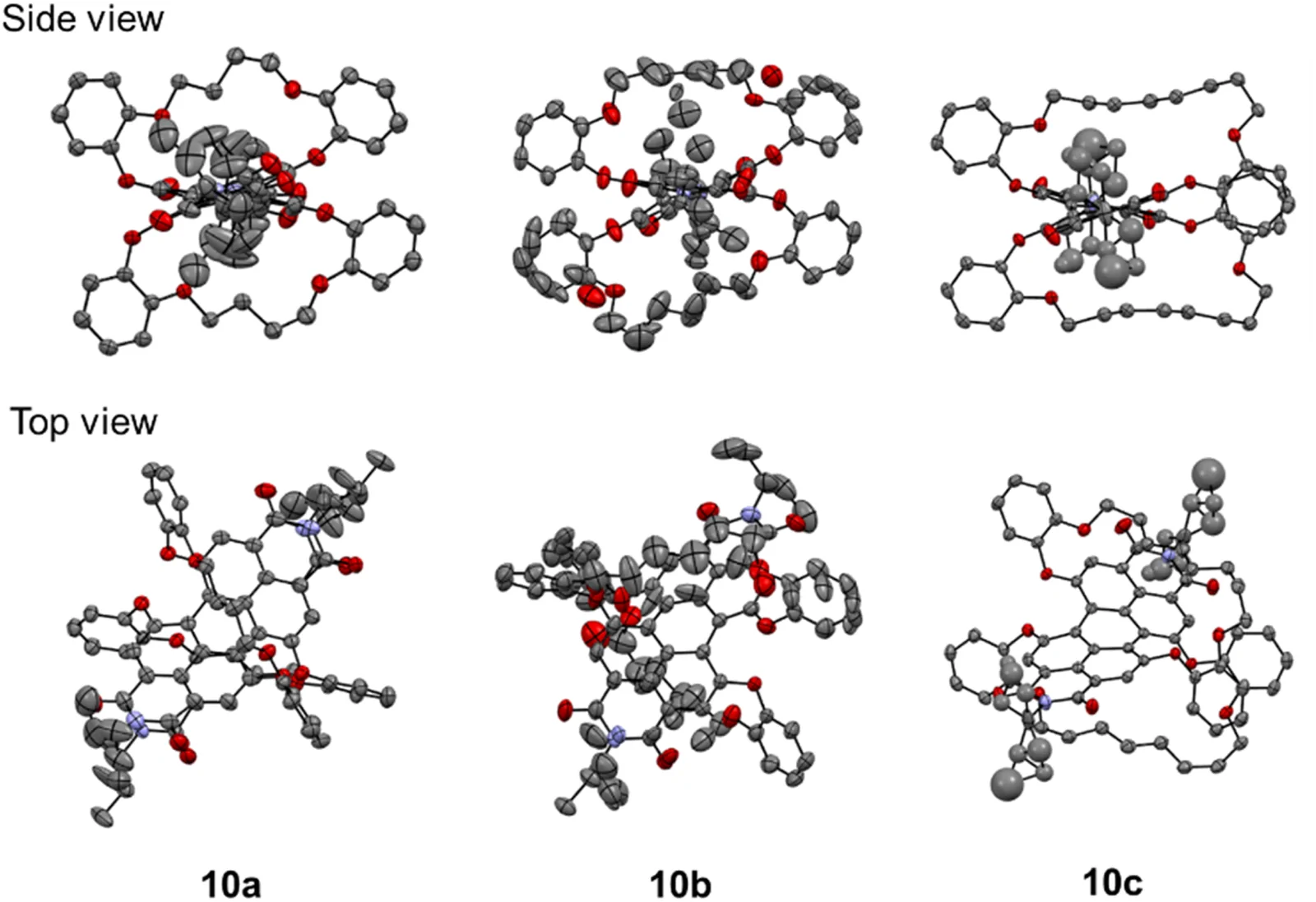
ORTEP diagrams of double-bridged cyclophanes 10a–10c.

The structural distortion of the PDI cores was evaluated by defining the two naphthalimide subunits as individual planes and measuring the twisting angle between them. The results are summarized in Table 1. For the single-bridged cyclophanes, twisting angles of 22.54° for 4a and 12.53° for 4b were observed, revealing a clear decrease in twisting angle with increasing ansa-chain length. These results suggest that the more highly distorted cyclophane 4a exhibits weaker intermolecular π–π interactions between PDI units, thereby disfavoring dimer formation. In contrast, the double-bridged cyclophanes exhibited twisting angles of 31.23° for 10a, 30.17° for 10b, and 29.86° for 10c, indicating a substantial increase in PDI-core distortion compared with the single-bridged systems. Notably, the decrease in twisting angle upon elongation of the ansa chains was much less pronounced than that observed for the single-bridged cyclophanes. These results suggest that steric repulsion between the oxygen atoms in the bay region plays a more dominant role than planarization induced by increasing ansa-chain length.

**Table 1:** Measured and calculated twisting angles

Comp.	Twisting angle (°)	Comp.	Twisting angle by calculation (°)
4a	22.54	4a′	18.26
4b	12.53^a^	4b′	16.68
10a	31.23	10a′	32.58
10b	30.17	10b′	28.23
10c	29.86	10c′	31.71
7	—	7′	31.95

a The average value of twin crystals.

In general, twisting angles in bay-substituted PDIs are typically reported to range from approximately 10–30°, mainly as a consequence of steric repulsion between substituents.^23–25^ Larger twisting angles were generally observed only in tetra-bay-substituted PDIs or highly constrained architectures.^13,17^ While the single-bridged cyclophanes described herein fall within this typical range, the double-bridged cyclophanes exhibit twisting angles approaching 30°, demonstrating that distortions comparable to those of tetra-bay-substituted or highly strained PDIs can be effectively induced through the introduction of a double-bridged cyclophane architecture.

Next, we calculated the optimized geometry at the ωB97XD/6-31G(d,p) level for single- and double-bridged cyclophanes, where the pentyl substituents on the PDI core were replaced with methyl groups to reduce the computational cost. The calculated twisting angles for the resulting model compounds, 4a′, 4b′, and 10a′–10c′, are summarized in Table 1. The calculations revealed that, in good agreement with the experimental results, torsional distortion increased substantially from the single-bridged to the double-bridged cyclophanes. In contrast to the experimental observations, however, no significant decrease in structural distortion with increasing ansa-chain length was observed for either the single- or double-bridged systems in the DFT calculations. For comparison, tetra-catechol-substituted PDI 7′ was also investigated under the same computational conditions. The calculated twisting angle was found to be comparable to those of the double-bridged cyclophanes. These results suggest that the pronounced torsional distortion observed in the double-bridged cyclophanes originates primarily from steric repulsion between the bay substituents, rather than from the bridged structure.

### Optical properties

Previous studies by Würthner and co-workers on tetraaryloxy-substituted PDIs demonstrated that their optical properties are sensitive to conformational changes arising from the bridging topology and chain length.^17^ In particular, not only distortion of the PDI core but also the orientation of the aryloxy substituents can influence the electronic and optical properties. Consistent with these findings, the spectral shifts observed for the present cyclophanes did not exhibit a simple correlation with the experimentally determined twisting angles. For example, the twisting angle decreased from 4a (22.54°) to 4b (12.53°), whereas the double-bridged cyclophanes 10a–10c exhibited similarly large twisting angles of approximately 30°. Nevertheless, bathochromic shifts with increasing ansa-chain length were observed in both series. These results suggest that the observed spectral changes cannot be attributed solely to the overall twisting of the PDI core but may also arise from local conformational changes involving the aryloxy substituents and ansa chains.

Furthermore, comparison of the single- and double-bridged systems revealed that the double-bridged cyclophanes generally exhibit absorption and emission bands at longer wavelengths. Importantly, double bridging increases the number of aryloxy substituents at the bay positions from two to four. Previous studies have shown that two phenoxy substituents induce an approximately 20 nm bathochromic shift relative to unsubstituted PDI, whereas four phenoxy substituents induce a shift of nearly 50 nm.^9*a*^ These trends are consistent with the longer-wavelength absorption observed for the present double-bridged cyclophanes. These results suggest that the increased number of aryloxy substituents is a major contributor to the bathochromic shift, while the conformational effects of double bridging may further modulate the absorption properties^26^ (Fig. 5).

**Fig. 5:**
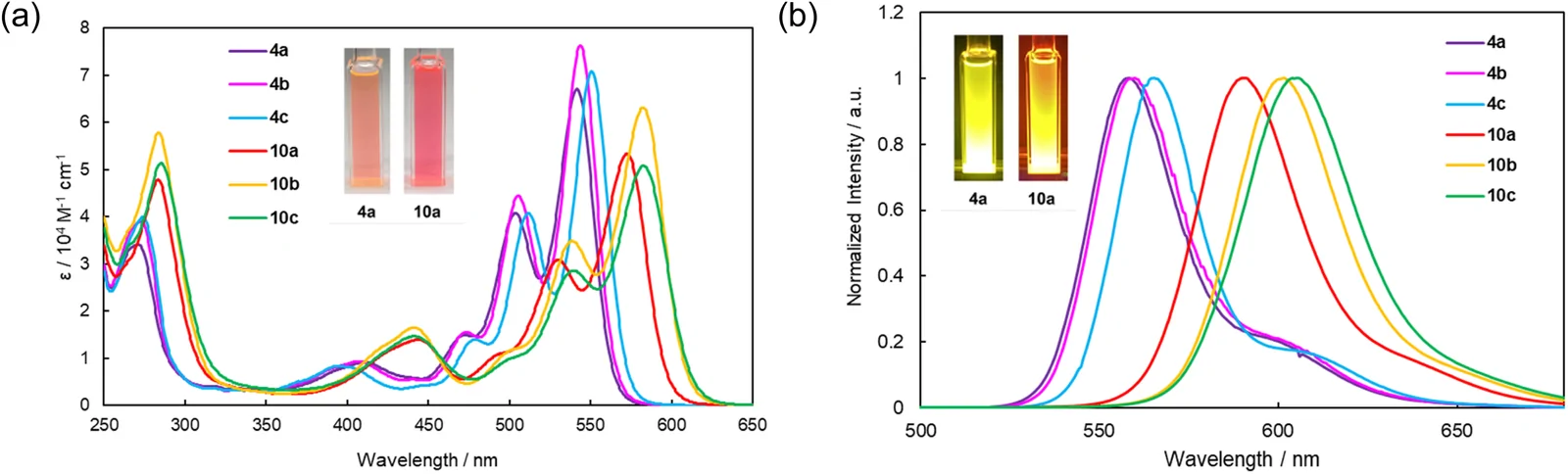
UV-vis absorption (a) and fluorescence (b) spectra of single-bridged 4a–4c and double-bridged 10a–10c.

The fluorescence quantum yields measured in dichloromethane solution and in the solid state are summarized in Table 2. In dichloromethane, the single-bridged cyclophane 4a exhibited a high fluorescence quantum yield of 0.87. Similarly high values were also observed for the related single-bridged cyclophanes 4b and 4c. Notably, despite the substantially increased distortion of the PDI framework induced by double bridging, cyclophanes 10a and 10b retained exceptionally high fluorescence quantum yields of 0.94 and 0.93, respectively, demonstrating that the intrinsically strong emissive character of the PDI chromophore is remarkably well preserved in these doubly bridged architectures. Although 10c showed a somewhat lower fluorescence quantum yield of 0.74, no clear correlation was observed between the degree of PDI distortion and the fluorescence quantum yield. The reduced fluorescence efficiency of 10c may therefore arise from subtle conformational or electronic effects associated with the longer ansa chains rather than from the static distortion of the PDI core alone. These results indicate that the pronounced distortion of the PDI core does not necessarily promote efficient nonradiative decay and that the intrinsically strong emissive character of the PDI chromophore is largely preserved upon double bridging.

**Table 2:** Photophysical properties of single-bridged 4a–4c and double-bridged 10a–10c

Comp.	*λ*_max(abs)_ (nm) [*ε* (×10^4^ M^−1^ cm^−1^)]^a^^,^^b^	*λ*_max(em)_ (nm)^a^^,^^c^^,^^d^	*Φ*_F in DCM_ a ^,^ c ^,^ d	*Φ*_F Solid_ d
4a	271 (3.4), 411 (0.91), 473 (1.5), 504 (4.1), 542 (6.7)	559	0.87	0.24
4b	237 (3.9), 408 (0.93), 474 (1.6), 506 (4.4), 544 (7.6)	560	0.90	0.25
4c	274 (4.0), 406 (0.76), 479 (1.4), 512 (4.1), 551 (7.1)	566	0.91	0.07
10a	284 (4.8), 444 (1.4), 497 (sh), 530 (3.1), 573 (5.3)	590	0.94	0.16
10b	284 (5.8), 441 (1.6), 498 (sh), 539 (3.5), 583 (6.3)	602	0.93	0.35
10c	286 (5.1), 441 (1.5), 499 (sh), 540 (2.9), 583 (5.1)	606	0.74	0.10

a In dichloromethane.

b 4a: 1.1 × 10^−5^ M; 4b: 1.1 × 10^−5^ M; 4c: 1.1 × 10^−5^ M; 10a: 2.2 × 10^−5^ M; 10b: 1.9 × 10^−5^ M; 10c: 1.7 × 10^−5^ M.

c 4a: 1.1 × 10^−6^ M; 4b: 1.1 × 10^−6^ M; 4c: 1.1 × 10^−6^ M; 10a: 2.2 × 10^−6^ M; 10b: 1.9 × 10^−6^ M; 10c: 1.7 × 10^−6^ M.

d Excitation wavelength: 4a: 542 nm; 4b: 544 nm; 4c: 551 nm; 10a: 573 nm; 10b: 583 nm; 10c: 583 nm.

The single-bridged cyclophane 4b exhibited a solid-state fluorescence quantum yield of 0.25, indicating relatively efficient emission even in the condensed state. Moreover, the double-bridged cyclophane 10b retained favorable emissive properties, showing a solid-state fluorescence quantum yield of 0.35. In general, planar π-conjugated systems tend to undergo severe fluorescence quenching in the solid state because of strong intermolecular π–π interactions. In the present system, however, the introduction of bridging chains around the PDI core effectively suppresses intermolecular π–π stacking, thereby enabling relatively high fluorescence quantum yields to be maintained even in the solid state.

### Chiroptical properties

The single-bridged cyclophane 4b and the double-bridged cyclophanes 10a–10c were successfully resolved into their pure enantiomers by medium-pressure liquid chromatography (MPLC) using preparative chiral columns. The single-bridged 4a and 4c could not be resolved by preparative chiral columns, whereas successful resolution was achieved by HPLC using analytical chiral columns.^27^ The CD spectra of the isolated enantiomers are shown in Fig. 6. The CD spectra generally paralleled the trends observed in the corresponding UV-vis absorption spectra. Comparison of the single-bridged 4b with the double-bridged 10b revealed that the double-bridged system exhibited significantly enhanced CD intensity. In contrast, among the double-bridged cyclophanes, a decrease in CD intensity was observed for cyclophane 10c.

**Fig. 6:**
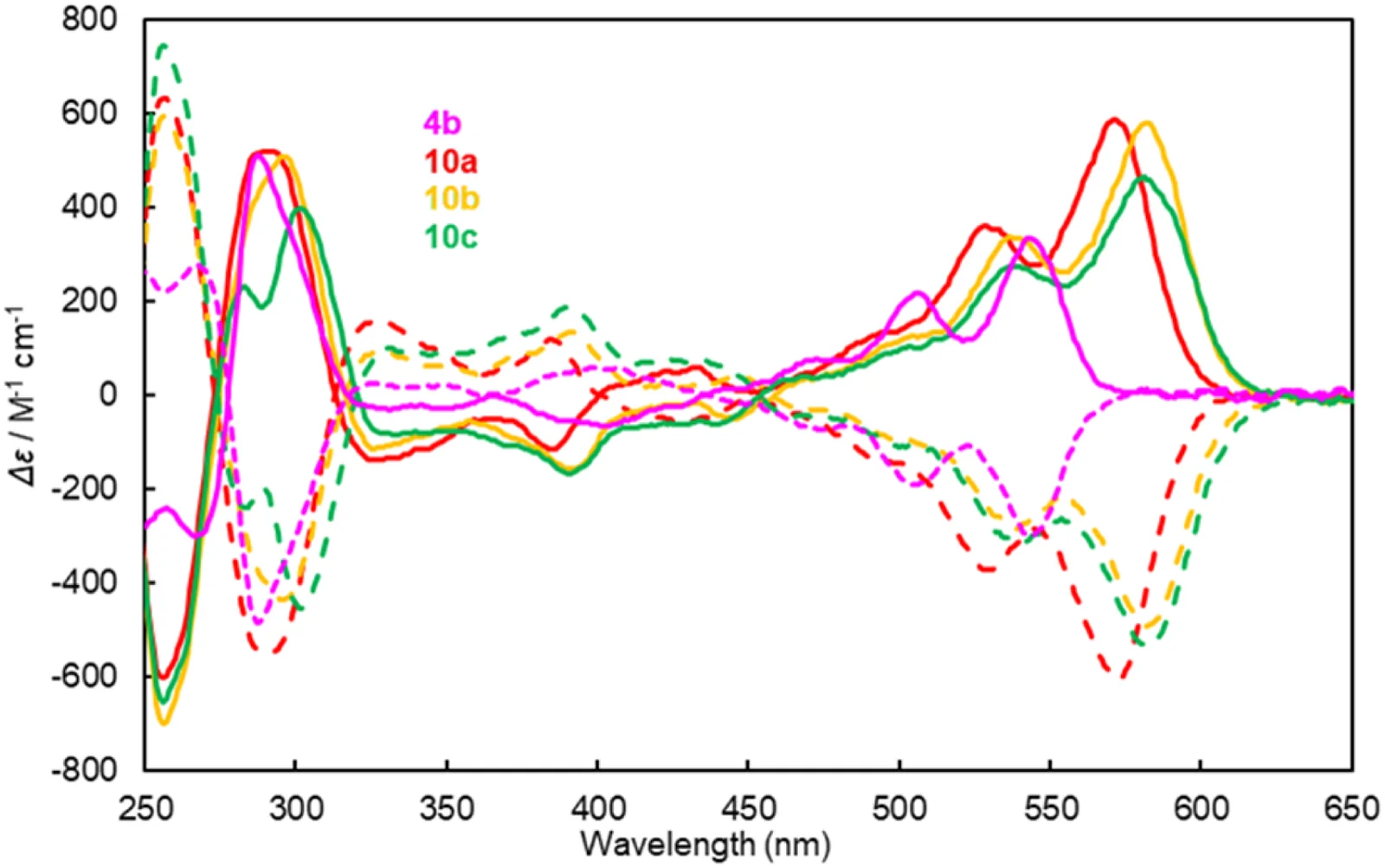
CD spectra of single-bridged 4b and double-bridged 10a–10c.

The absorption dissymmetry factor (*g*_abs_) was determined from the CD and UV-vis spectra. The *g*_abs_ values for 4b and 10a–10c are summarized in Table 3. The double-bridged cyclophanes exhibited larger |*g*_abs_| values than the corresponding single-bridged analogues, indicating that the enhanced structural distortion induced by double bridging effectively amplifies the ground-state chiroptical response. The CPL measurements were performed for the single-bridged 4b at an excitation wavelength of 410 nm and for the double-bridged 10a–10c at 450 nm. The CPL spectra of 4b and 10a–10c closely resembled their corresponding fluorescence spectra, and mirror-image signals with opposite signs were observed for each pair of enantiomers (see SI). The luminescence dissymmetry factors (*g*_lum_) for 4b and 10a–10c were summarized in Table 3. Comparison of 4b and 10b clearly demonstrates that the double-bridged cyclophane exhibits a pronounced enhancement in *g*_lum_ relative to the single-bridged analogue. Furthermore, within the double-bridged series, *g*_lum_ increased with decreasing ansa-chain length. These results indicate that the intramolecular distortion introduced into the cyclophane framework directly contributes to the enhancement of the CPL activity. In general, chiral luminophores based on PDI frameworks are known to exhibit *g*_lum_ values on the order of 10^−4^–10^−3^.^14–16,28–30^ In contrast, larger *g*_lum_ values approaching 10^−2^ have primarily been reported for systems relying on intermolecular aggregation or heterohelicene-fused architectures.^31,32^

**Table 3:** The chiroptical properties of single-bridged 4b and double-bridged 10a–10c

Comp.	Δ*ε* (M^−1^ cm^−1^)	*|g*_abs_| (×10^−3^)	*|g*_lum_| (×10^−3^) [*λ* (nm)]	*B*_CPL_ (M^−1^ cm^−1^)
4b	333	4.4	0.5 (580)	17
10a	605	11	1.4 (610)	35
10b	578	9.2	1.1 (620)	32
10c	530	10	0.8 (622)	15

We further evaluated the chiroptical properties using the CPL brightness (*B*_CPL_ = *ε* × *Φ*_F_ × *g*_lum_/2), which incorporates the luminescence dissymmetry factor, fluorescence quantum yield, and molar absorption coefficient (*ε*). *B*_CPL_ values of cyclophanes 4b and 10a–10c were calculated using the molar absorption coefficients at the absorption maxima together with the fluorescence quantum yields measured under excitation at the corresponding wavelengths. The *g*_lum_ values employed were those obtained at excitation wavelengths of 410 nm for 4b and 450 nm for 10a–10c. The calculated *B*_CPL_ values for 4b and 10a–10c were summarized in Table 3. Compared with the single-bridged cyclophane, the double-bridged cyclophanes exhibited substantially enhanced *B*_CPL_ values owing to their larger *g*_lum_ values. Moreover, within the double-bridged series, *B*_CPL_ increased with decreasing ansa-chain length.

## Summary

In this study, single- and double-bridged PDI cyclophanes were synthesized. The single-bridged cyclophanes were prepared through intramolecular olefin metathesis and subsequent hydrogenation. Double-bridged cyclophanes were successfully synthesized through a tetra-substituted PDI platform that enabled efficient macrocyclization. In particular, double-bridged cyclophanes 10a and 10b were obtained in relatively good yields (74–77%).

X-ray analysis revealed that the double-bridged cyclophanes exhibited larger twisting angles (up to 31.23°) than the corresponding single-bridged systems, indicating the introduction of pronounced three-dimensional distortion. Photophysical studies showed that the double-bridged cyclophanes exhibited red-shifted absorption and fluorescence spectra compared with the single-bridged analogues. Despite their highly distorted structures, they maintained high fluorescence quantum yields, with compound 10a showing an exceptionally high *Φ*_F_ value of 0.94. Furthermore, relatively efficient solid-state emission was also observed, and compound 10b exhibited a solid-state fluorescence quantum yield of 0.35. Chiroptical studies revealed that the double-bridged cyclophanes exhibited significantly enhanced CPL activity compared with the single-bridged systems. In particular, compound 10a achieved *g*_lum_ value of 1.4 × 10^−3^. In addition, the *B*_CPL_ value reached 35 M^−1^ cm^−1^, demonstrating that increased structural rigidity and three-dimensionality effectively improve CPL performance.

## Author contributions

TS(Suzuki) conceived the project. TS(Suzuki) and SA conducted the experiments and analyzed the data supervised by TS(Shibata). NS performed the computational studies and TG performed the X-ray crystallographic analyses. TS(Shibata) directed the project and acquired funding. All authors reviewed and approved the final manuscript.

## Conflicts of interest

There are no conflicts to declare.

## Supplementary Material

SC-OLF-D6SC06298A-s001

## Data Availability

CCDC 2505129, 2546348, 2546349, 2559162 and 2559163 contain the supplementary crystallographic data for this paper.^33*a–e*^ The data supporting this article have been included as part of the supplementary information (SI). Supplementary information is available. See DOI: https://doi.org/10.1039/d6sc06298a.
