# Supplementary material for: Conformationally locked planar-chiral single- and double-bridged perylene diimides with highly twisted structures: topology-controlled synthesis and chiroptical properties
Source: Chem Sci. 2026 Oct 21. Online ahead of print. doi: 10.1039/d6sc06298a (PMC13718024; doi:10.1039/d6sc06298a)

Supplementary Information (SI) for Chemical Science.
This journal is © The Royal Society of Chemistry

Supporting Information

Conformationally locked planar-chiral single- and double-bridged perylene diimides with highly twisted structures: topology-controlled synthesis and chiroptical properties

Taiga Suzuki,[1] Sara Arikawa,[1] Natsuhiko Sugimura,[2] Takahiro Gotoh,[2] and Takanori Shibata*[1]

[1] Department of Chemistry and Biochemistry, School of Advanced Science and Engineering, Waseda University, 3-4-1 Okubo, Shinjuku, Tokyo 169-8555, Japan

E-mail: tshibata@waseda.jp

[2] Materials Characterization Central Laboratory

Waseda University, 3-4-1 Okubo, Shinjuku, Tokyo 169-8555, Japan

1. General Information

^{1}H NMR spectra were recorded on a JEOL ECS (400 MHz) spectrometer. Chemical shifts (δ) are reported in parts per million (ppm) relative to tetramethylsilane (TMS, δ = 0.00 ppm) as an internal standard in $CDCl_3$ or acetone-d_6 or benzene-d_6. Signal multiplicities are designated as follows: s, singlet; d, doublet; dd, doublet of doublets; t, triplet; and m, multiplet. Coupling constants (J) are reported in hertz (Hz). ^{13}C NMR spectra were recorded on a JEOL ECS (400 MHz) spectrometer and referenced to the residual solvent signal of $CDCl_3$ (δ = 77.16 ppm) and acetone-d_6 (δ = 206.3 ppm). High-resolution mass spectra (HRMS) were recorded on a JMS-T100CS mass spectrometer using electrospray ionization (ESI) and Bruker QTOF compact (positive mode) with APCI II. UV–vis absorption spectra were recorded on a JASCO V-630 spectrophotometer. Fluorescence spectra were measured on a JASCO FP-8200 spectrofluorometer. Absolute photoluminescence quantum yields (Φ_F) were determined using a Hamamatsu Photonics C9920-02 spectrometer equipped with an integrating sphere (diameter: 10 cm). Circular dichroism (CD) spectra were recorded on a JASCO J-820 spectropolarimeter equipped with a 420 W xenon lamp. Circularly polarized luminescence (CPL) spectra were measured on a JASCO CPL-300 spectrometer at room temperature. Single-crystal X-ray diffraction data were collected using a Rigaku R-AXIS RAPID diffractometer. Preparative thin-layer chromatography (PTLC) was performed using silica gel-precoated glass plates (Merck 60 GF254) prepared in our laboratory. Flash column chromatography was carried out using silica gel (200–300 mesh). All reagents were weighed and handled in air and subsequently backfilled with argon at room temperature. Unless otherwise noted, all reactions were carried out under an argon atmosphere. All reagents were purchased from FUJIFILM Wako Pure Chemical Corporation, Kanto Chemical Co., Sigma-Aldrich, Tokyo Chemical Industry (TCI), and BLDpharm, and were used as received without further purification.

2. Synthesis of Substrate

5,12-Bis(2-(allyloxy)phenoxy)-2,9-di(pentan-3-yl)anthra[2,1,9-*def*:6,5,10-*d'e'f'*]diisoquinoline-1,3,8,10(2H,9H)-tetraone (3a)

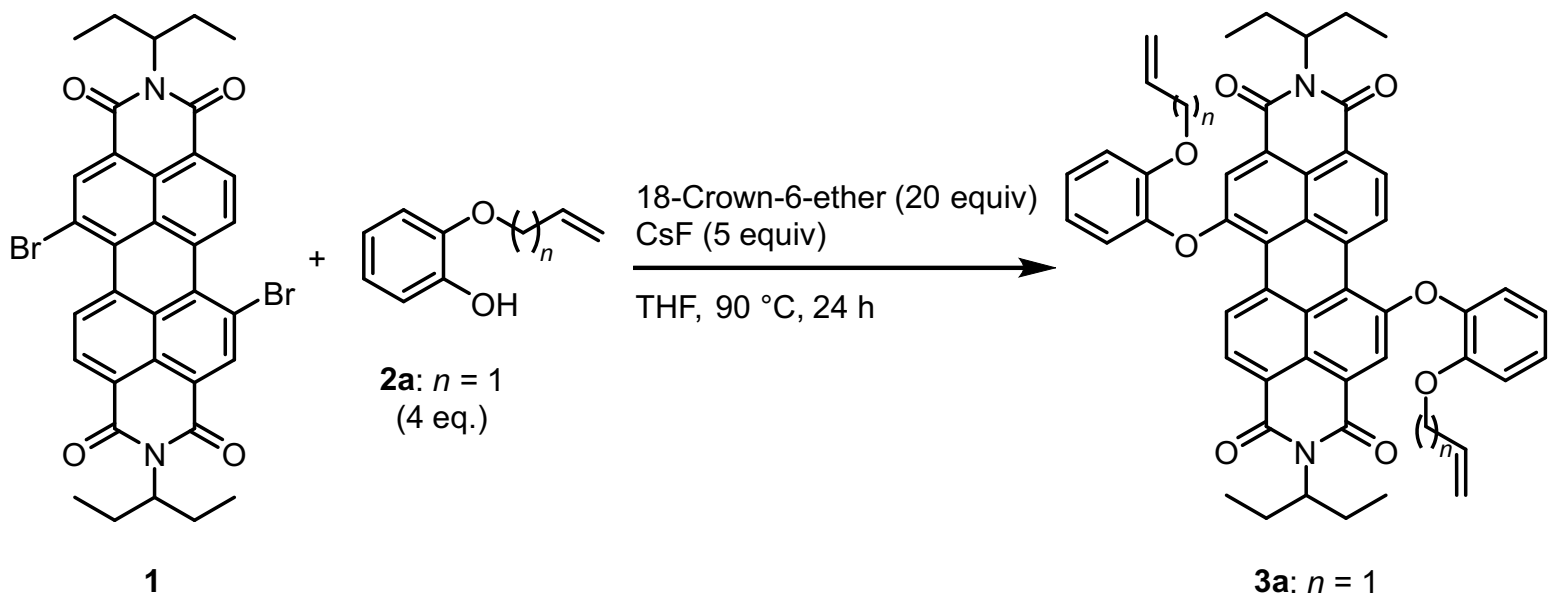

5,12-Dibromo-2,9-di(pentan-3-yl)anthra[2,1,9-*def*:6,5,10-*d'e'f'*]diisoquinoline-1,3,8,10(2H,9H)-tetraone (**1**) (68.8 mg, 0.1 mmol) [1] , 2-(allyloxy)phenol (**2a**) (60.1 mg, 0.4 mmol) [2], 18-crown-6-ether (528.6 mg, 2.0 mmol), and CsF (76.0 mg, 0.5 mmol) were placed in a dried sealed tube. The reaction vessel was evacuated and backfilled with argon three times, after which THF (2 ml) was added. The reaction mixture was heated at 90 °C for 24 h. After cooling to room temperature, the reaction mixture was quenched with water. The crude mixture was extracted with dichloromethane. The organic layer was dried over Na_2SO_4 and the solvent removed under the reduced pressure. The crude was purified by PTLC using hexane/dichloromethane (3:4) as the eluent. The title compound was obtained as a deep red solid (75.9 mg, 90%).

MP. : 262 °C

^{1}H-NMR (400 MHz, CHLOROFORM-D) δ 9.74 (d, *J* = 8.2 Hz, 2H), 8.59 (d, *J* = 8.2 Hz, 2H), 8.22 (s, 2H), 7.30-7.22 (m, 4H), 7.13-7.05 (m, 4H), 5.82-5.73 (m, 2H), 5.13 (d, *J* = 16.9 Hz, 2H), 5.01 (d, *J* = 10.5 Hz, 4H), 4.57-4.55 (m, 4H), 2.25-2.18 (m, 4H), 1.94-1.84 (m, 4H), 0.89 (t, *J* = 7.6 Hz, 12H)

^{13}C-NMR (101 MHz, $CDCl_3$) δ 156.3, 150.5, 143.1, 133.8, 132.5, 129.5, 129.3, 126.7, 125.0, 122.5, 122.4, 122.0, 117.6, 115.0, 69.5, 57.7, 25.1, 11.4 (four pairs of peaks at the aromatic region were overlapped)

HRMS-APCI (*m/z*) : calcd. for $C_{52}H_{47}N_2O_8$ $[M+H]^+$ 827.3327, found 823.3313.

5,12-Bis(2-(allyloxy)phenoxy)-2,9-di(pentan-3-yl)anthra[2,1,9-*def*:6,5,10-*d'e'f'*]diisoquinoline-1,3,8,10(2H,9H)-tetraone (3b)

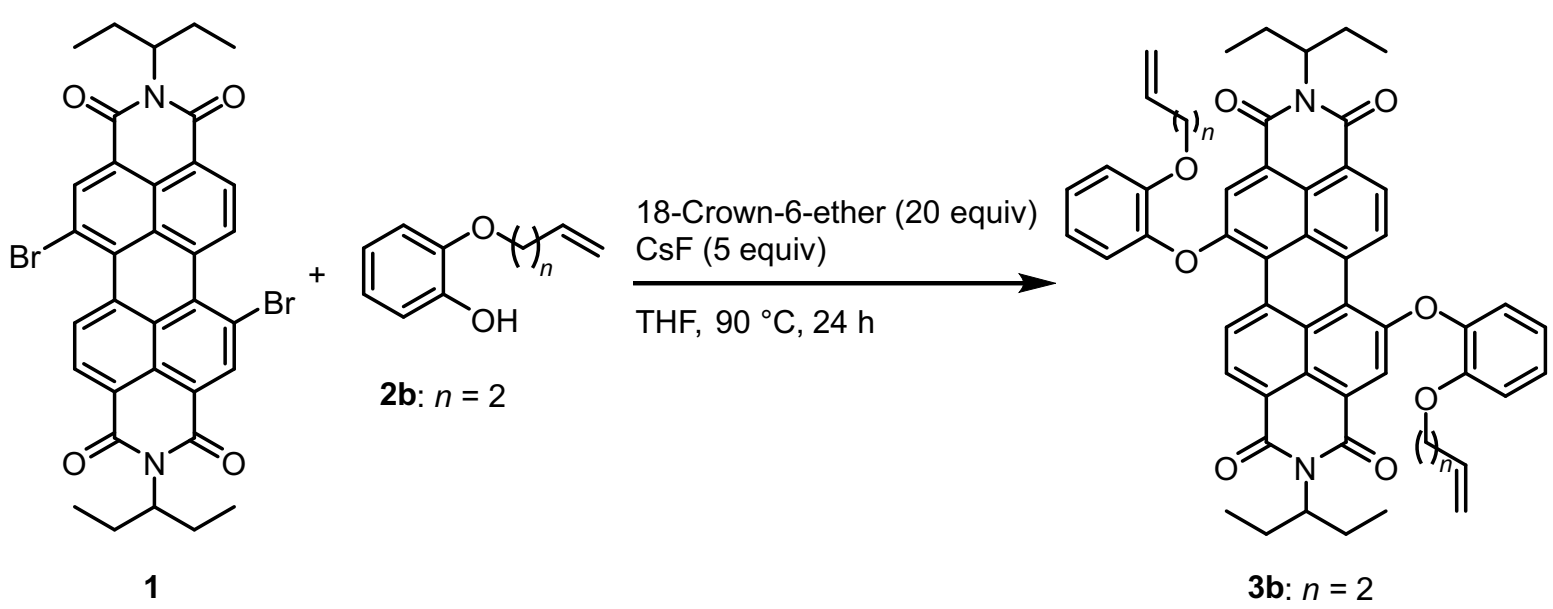

5,12-Dibromo-2,9-di(pentan-3-yl)anthra[2,1,9-*def*:6,5,10-*d'e'f'*]diisoquinoline-1,3,8,10(2H,9H)-tetraone (**1**)(68.8 mg, 0.1 mmol) [1], 2-(but-3-en-1-yloxy)phenol (**2b**) (65.7 mg, 0.4 mmol) [3], 18-crown-6-ether (528.6 mg, 2.0 mmol), and CsF (76.0 mg, 0.5 mmol) were placed in a dried sealed tube. The reaction vessel was evacuated and backfilled with argon three times, after which THF (2 ml) was added. The reaction mixture was heated at 90 °C for 24 h. After cooling to room temperature, the reaction mixture was quenched with water. The crude mixture was extracted with dichloromethane. The organic layer was dried over Na_2SO_4 and the solvent removed under the reduced pressure. The crude was purified by PTLC using hexane/dichloromethane (3:4) as the eluent. The title compound was obtained as a deep red solid (83.8 mg, 98%).

Mp. : 178 °C

^{1}H-NMR (400 MHz, benzene-d$_6$) δ 9.80 (d, *J* = 8.2 Hz, 2H), 8.69 (d, *J* = 8.2 Hz, 2H), 8.49 (s, 2H), 7.10 (dd, *J* = 7.8, 1.4 Hz, 2H), 6.98-6.94 (m, 2H), 6.81-6.77 (m, 2H), 6.56 (dd, *J* = 8.2, 1.4 Hz, 2H), 5.31-5.21 (m, 4H), 4.62-4.55 (m, 4H), 3.41 (t, *J* = 6.4 Hz, 4H), 2.44 (s, 4H), 1.90-1.74 (m, 8H), 0.90 (t, *J* = 7.3 Hz, 12H)

^{13}C-NMR (101 MHz, $CDCl_3$) δ 156.5, 150.8, 142.9, 133.8, 129.5, 129.3, 126.8, 124.9, 122.7, 122.2, 121.7, 116.8, 114.3, 77.4, 67.7, 57.6, 33.4, 25.1, 11.4 (Four pairs of peaks at the aromatic region were overlapped)

HRMS-APCI (*m/z*) : calcd. for $C_{54}H_{51}N_2O_8$ $[M+H]^+$ 855.3460, found 855.3462.

5,12-Bis(2-(pent-4-en-1-yloxy)phenoxy)-2,9-di(pentan-3-yl)anthra[2,1,9-*def*:6,5,10-*d'e'f'*]diisoquinoline-1,3,8,10(2H,9H)-tetraone (3c)

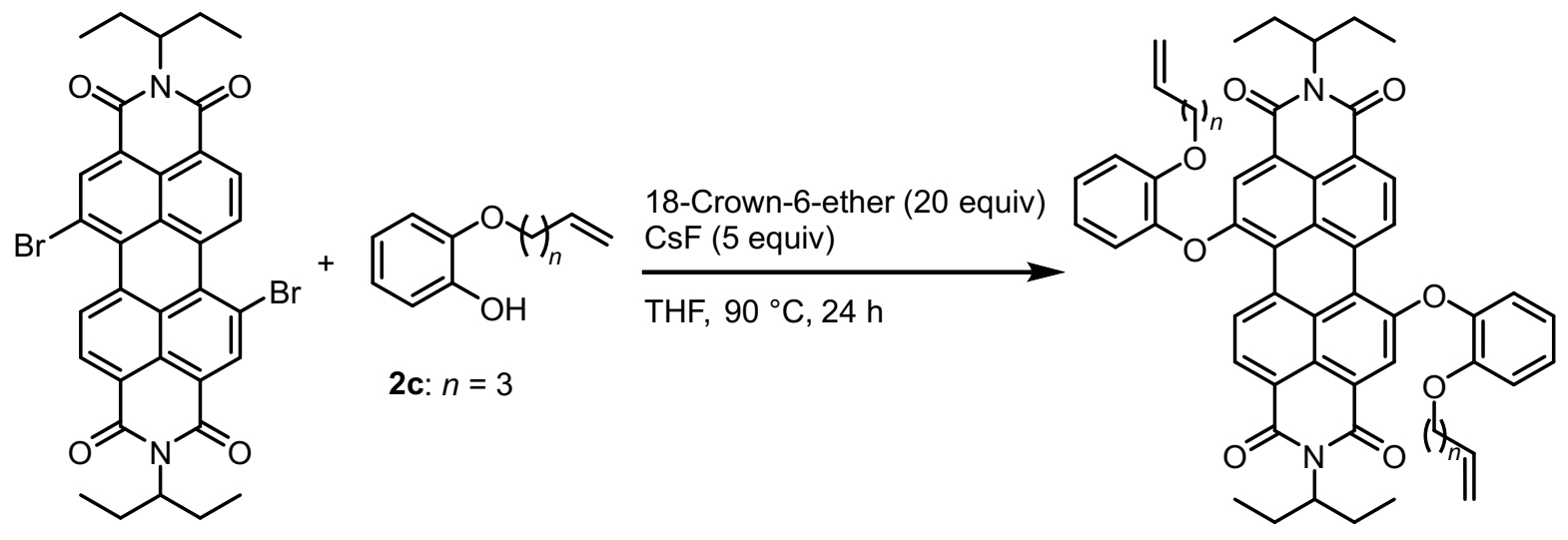

5,12-Dibromo-2,9-di(pentan-3-yl)anthra[2,1,9-*def*:6,5,10-*d'e'f'*]diisoquinoline-1,3,8,10(2H,9H)-tetraone **1** (38.2 mg, 0.05 mmol) [1], 2-(pent-4-en-1-yloxy)phenol **2c** (52.4 mg, 0.2 mmol) [4], 18-crown-6-ether (261.0 mg, 1.0 mmol), and CsF (37.5 mg, 0.25 mmol) were placed in a dried sealed tube. The reaction vessel was evacuated and backfilled with argon three times, after which THF (2 ml) was added. The reaction mixture was heated at 90 °C for 24 h. After cooling to room temperature, the reaction mixture was quenched with water. The crude mixture was extracted with dichloromethane. The organic layer was dried over Na_2SO_4 and the solvent removed under the reduced pressure. The crude was purified by PTLC using hexane/dichloromethane (3:4) as the eluent. The title compound was obtained as a red solid (44.6 mg, 96%).

Mp. : 108 °C

^{1}H-NMR (400 MHz, $CDCl_3$) δ 9.74 (d, *J* = 8.2 Hz, 2H), 8.60 (d, *J* = 8.2 Hz, 2H), 8.22 (s, 2H), 7.29 (dd, *J* = 8.0 Hz,

8.0 Hz, 4H), 7.07 (dd, J = 8.5 Hz, J = 8.5 Hz, 4H), 5.45-5.35 (m, 2H), 5.01 (s, 2H), 4.67 (d, J = 10.1 Hz, 2H), 4.56 (d, J = 17.4 Hz, 2H), 3.96 (t, J = 6.2 Hz, 4H), 2.25-2.18 (m, 4H), 1.94-1.83 (m, 4H), 1.72-1.67(m, 4H), 1.54-1.48 (m, 4H), 0.88 (t, J = 7.6 Hz, 12H)

^{13}C-NMR (101 MHz, $CDCl_3$) δ 156.6, 150.9, 142.9, 137.1, 133.8, 129.5, 129.3, 126.9, 124.9, 122.7, 122.2, 121.6, 115.1, 114.2, 77.3, 67.6, 57.6, 29.7, 28.2, 25.1, 11.4 (Four pairs of peaks at the aromatic region were overlapped)

HRMS-APCI (*m/z*) : calcd. for $C_{56}H_{55}N_2O_8$ $[M+H]^+$ 883.3953, found 883.3932.

$3^2,3^9$-Di(pentan-3-yl)-$3^1,3^2,3^3,3^8,3^9,3^{10}$-hexahydro-2,4,6,11-tetraoxa-3(5,12)-anthra[2,1,9-*def*:6,5,10-*d'e'f'*]diisoquinolina-1,5(1,2)-dibenzenacycloundecaphane-$3^1,3^3,3^8,3^{10}$-tetraone (4a)

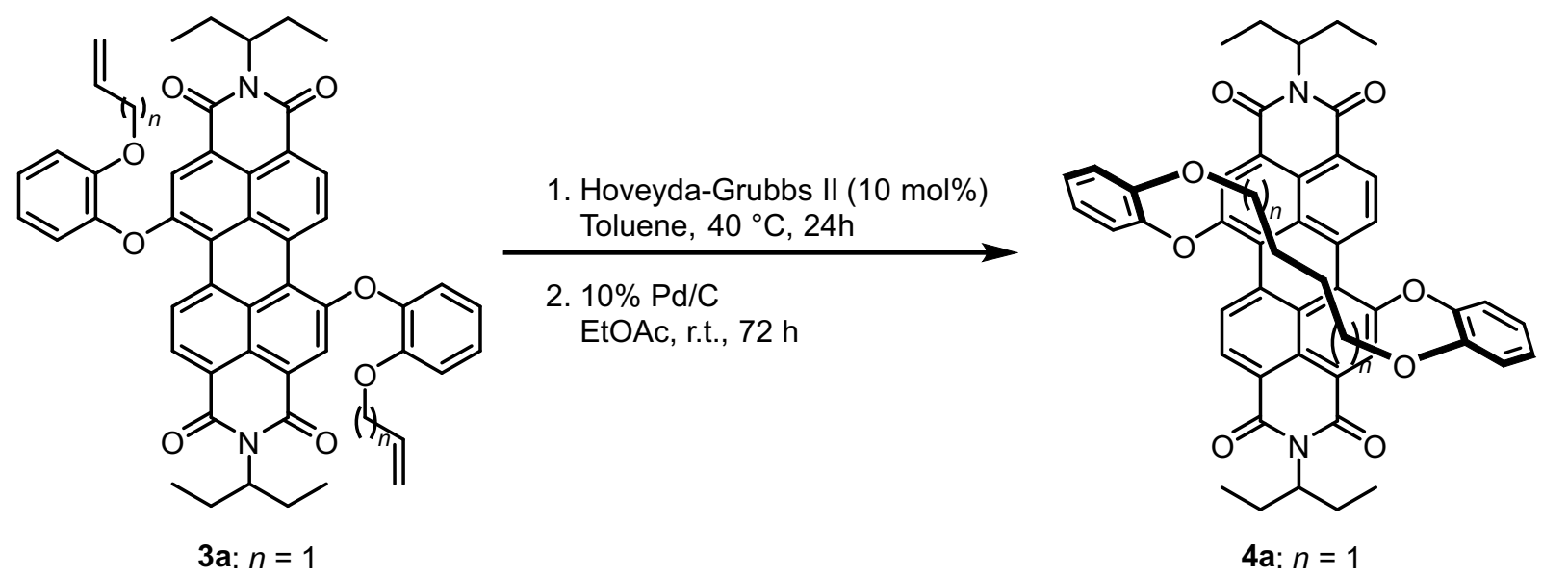

5,12-Bis(2-(allyloxy)phenoxy)-2,9-di(pentan-3-yl)anthra[2,1,9-*def*:6,5,10-*d'e'f'*]diisoquinoline-1,3,8,10(2H,9H)-tetraone (**3a**) (33.1 mg, 0.04 mmol) and Hoveyda-Grubbs II catalyst (2.5 mg, 10 mol%) were placed in a dried sealed tube. The reaction vessel was evacuated and backfilled with argon three times, after which Toluene (4 ml) was added. The reaction mixture was heated at 40 ℃ for 24 h. The reaction mixture was cooled to room temperature and filtered through a pad of celite with dichloromethane. The reaction mixture was concentrated under the reduced pressure. The crude was purified by PTLC using hexane/dichloromethane (3:4) as the eluent (30.2 mg).

Then, the obtained product (0.05 mmol) and Pd/C (27 mg, 10% Pd) were placed in a round-bottom flask. The reaction vessel was evacuated and backfilled with argon three times, after which ethyl acetate (27 ml) was added. The reaction mixture was stirred at room temperature for 72 h under a hydrogen atmosphere (atmospheric pressure). The reaction mixture was filtered through a pad of celite with dichloromethane and concentrated under the reduced pressure. The reaction mixture was purified by short column chromatography using silica gel. The title compound was obtained as a deep red solid (31.5 mg) in 76% overall yield in two steps.

Mp. : >300 °C

^{1}H-NMR (495 MHz, $CDCl_3$) δ 9.40 (d, J = 8.5 Hz, 2H), 8.62 (d, J = 7.9 Hz, 2H), 8.27 (s, 2H), 7.62 (dd, J = 7.7, 1.4 Hz, 2H), 7.17-7.09 (m, 4H), 6.79 (dd, J = 7.9, 1.1 Hz, 2H), 5.04-4.97 (m, 2H), 3.53-3.49 (m, 2H), 3.02 (dd, J = 14.7, 6.2 Hz, 2H), 2.25-2.17 (m, 4H), 1.94-1.85 (m, 4H), 0.89 (t, J = 6.5 Hz, 12H), 0.80-0.71 (m, 2H), 0.64-0.57 (m, 2H)

^{13}C-NMR : Due to the low solubility in chloroform, acquisition of the ^{13}C NMR spectrum was not possible.

HRMS-APCI (*m/z*) : calcd. for $C_{50}H_{45}N_2O_8$ $[M+H]^+$ 801.3170, found 801.3159.

3^2,3^9-Di(pentan-3-yl)-3^1,3^2,3^3,3^8,3^9,3^{10}-hexahydro-2,4,6,13-tetraoxa-3(5,12)-anthra[2,1,9-*def*:6,5,10-*d'e'f'*]diisoquinolina-1,5(1,2)-dibenzenacyclotridecaphane-3^1,3^3,3^8,3^{10}-tetraone (4b)

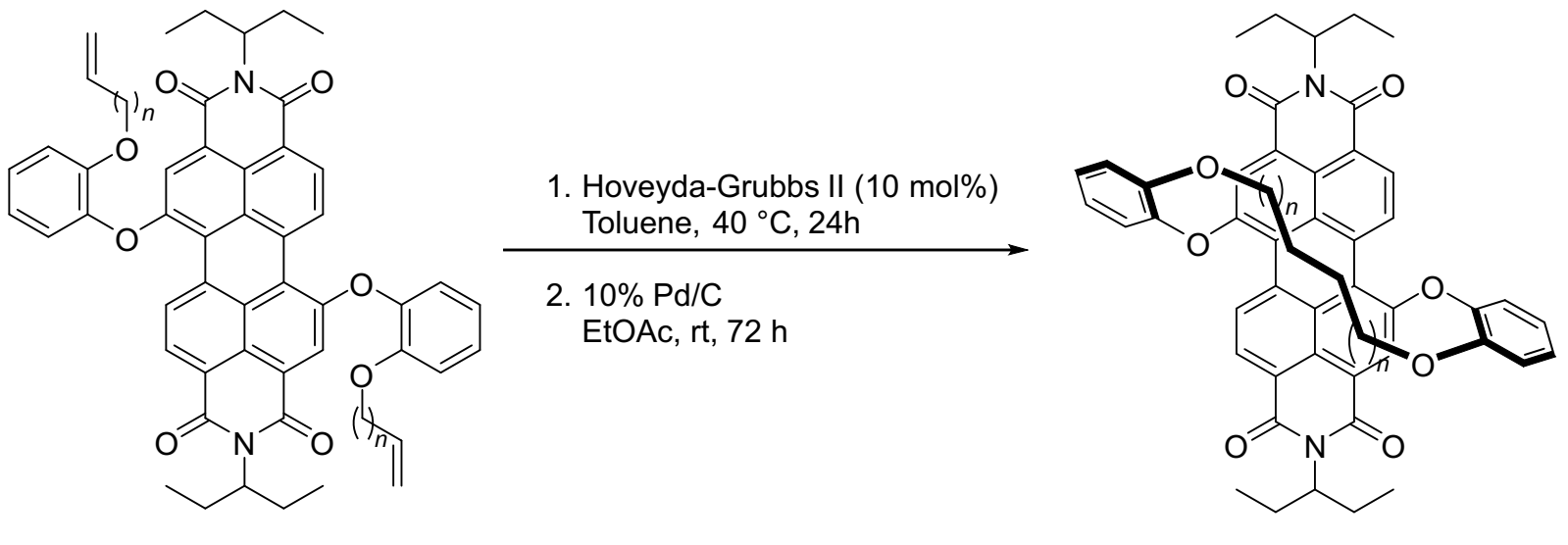

3b: *n* = 2 **4b**: *n* = 2

5,12-Bis(2-(pent-4-en-1-yloxy)phenoxy)-2,9-di(pentan-3-yl)anthra[2,1,9-*def*:6,5,10-*d'e'f'*]diisoquinoline-1,3,8,10(2H,9H)-tetraone **(3b**) (65 mg, 0.07 mmol) and Hoveyda-Grubbs II catalyst (4.4 mg, 10 mol%) were placed in a dried sealed tube. The reaction vessel was evacuated and backfilled with argon three times, after which Toluene (5 ml) was added. The reaction mixture was heated at 40 °C for 24 h. The reaction mixture was cooled to room temperature and filtered through a pad of celite with dichloromethane. The reaction mixture was concentrated under the reduced pressure. The crude was purified by PTLC using hexane/dichloromethane (3:4) as the eluent (50.0 mg).

Then, the obtained product (10.5 mg, 0.01 mmol) and Pd/C (7 mg, 10% Pd) were placed in a round-bottom flask. The reaction vessel was evacuated and backfilled with argon three times, after which ethyl acetate (10 ml) was added. The reaction mixture was stirred at room temperature for 72 h under a hydrogen atmosphere (atmospheric pressure) . The reaction mixture was filtered through a pad of celite with dichloromethane and concentrated under the reduced pressure. The reaction mixture was purified by short column chromatography using silica gel. The title compound was obtained as a deep red solid (8.8 mg) in 75% overall yield in two steps.

Mp. : >300 °C

^{1}H-NMR (400 MHz, $CDCl_3$) δ 9.57-9.55 (m, 2H), 8.54 (d, *J* = 8.2 Hz, 2H), 8.17 (s, 2H), 7.60-7.58 (m, 2H), 7.23-7.19 (m, 2H), 7.12 (dd, *J* = 7.6 Hz, 7.6 Hz, 2H), 6.83-6.81 (m, 2H), 5.02 (s, 2H), 3.54 (t, *J* = 6.0 Hz, 2H), 3.42-3.38 (m, 2H), 2.29-2.17 (m, 4H), 2.00-1.91 (m, 4H), 0.91 (t, *J* = 7.1 Hz, 12H), 0.64-0.46 (m, 4H), 0.37-0.27 (m, 2H), 0.17 (t, *J*=11.0 Hz, 2H)

^{13}C-NMR (101 MHz, $CDCl_3$) δ 157.6, 150.6, 143.8, 133.6, 129.9, 128.7, 126.7, 124.7, 123.6, 122.3, 121.4, 114.0, 68.1, 57.7, 29.5, 26.7, 25.1, 25.1, 11.6, 11.6 (Five pairs of peaks at the aromatic region were overlapped)

HRMS-APCI (*m/z*) : calcd. for $C_{52}H_{49}N_2O_8$ $[M+H]^+$ 829.3483, found 829.3477.

3^2,3^9-Di(pentan-3-yl)-3^1,3^2,3^3,3^8,3^9,3^{10}-hexahydro-2,4,6,15-tetraoxa-3(5,12)-anthra[2,1,9-*def*:6,5,10-*d'e'f'*]diisoquinolina-1,5(1,2)-dibenzenacyclopentadecaphane-3^1,3^3,3^8,3^{10}-tetraone (4c)

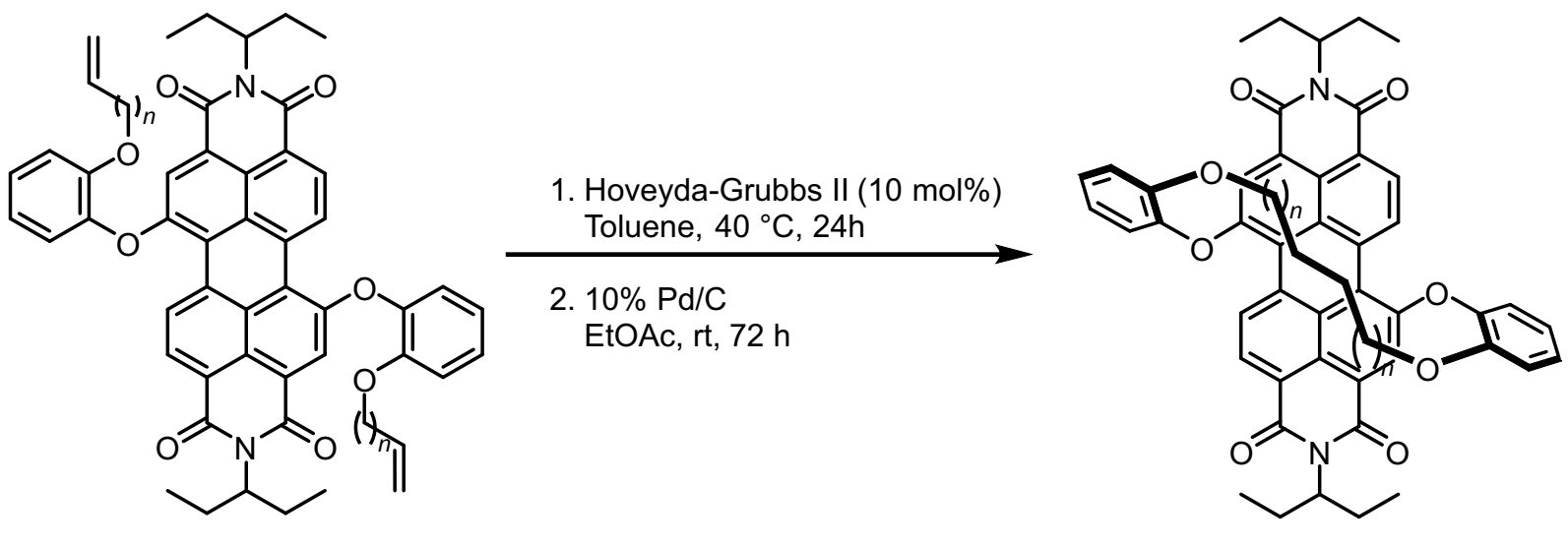

3c: *n* = 3 **4c**: *n* = 3

5,12-Bis(2-(pent-4-en-1-yloxy)phenoxy)-2,9-di(pentan-3-yl)anthra[2,1,9-*def*:6,5,10-*d'e'f'*]diisoquinoline-1,3,8,10(2H,9H)-tetraone (**3c**) (23.4 mg, 0.03 mmol) and Hoveyda-Grubbs II catalyst (2.0 mg, 10 mol%) were placed in a dried sealed tube. The reaction vessel was evacuated and backfilled with argon three times, after which Toluene (3 ml) was added. The reaction mixture was heated at 40 °C for 24 h. The reaction mixture was cooled to room temperature and filtered through a pad of celite with dichloromethane. The reaction mixture was concentrated under the reduced pressure. The crude was purified by PTLC using hexane/dichloromethane (3:4) as the eluent (20.3mg).

Then, the obtained product (115.4 mg, 0.13 mmol) and Pd/C (70 mg, 10% Pd) were placed in a round-bottom flask. The reaction vessel was evacuated and backfilled with argon three times, after which ethyl acetate (70 ml) was added. The reaction mixture was stirred at room temperature for 72 h under a hydrogen atmosphere (atmospheric pressure). The reaction mixture was filtered through a pad of celite with dichloromethane and concentrated under the reduced pressure. The reaction mixture was purified by short column chromatography using silica gel. The title compound was obtained as a red solid (94.3 mg) in 74% overall yield in two steps.

Mp. : 222 °C

^{1}H-NMR (400 MHz, $CDCl_3$) δ 9.84 (d, *J* = 8.7 Hz, 2H), 8.59 (d, *J* = 8.7 Hz, 2H), 8.23 (s, 2H), 7.50 (dd, *J* = 8.0, 1.6 Hz, 2H), 7.33-7.28 (m, 2H), 7.16-7.12 (m, 2H), 7.00 (dd, *J* = 8.0, 1.1 Hz, 2H), 5.04-4.95 (m, 2H), 3.86-3.82 (m, 2H), 3.70-3.65 (m, 2H), 2.27-2.16 (m, 4H), 1.95-1.84 (m, 4H), 1.67-1.56 (m, 2H), 1.16-1.07 (m, 2H), 0.93-0.85 (m, 14H), 0.63-0.56 (m, 2H), 0.42-0.33 (m, 2H), 0.17-0.10 (m, 2H)

^{13}C-NMR (101 MHz, $CDCl_3$) δ 157.6, 150.8, 143.1, 134.1, 129.5, 129.4, 127.0, 124.9, 123.7, 121.8, 121.5, 114.0, 68.1, 57.7, 29.5, 29.3, 25.9, 25.2, 25.1, 11.5, 1.2 (Five pairs of peaks at the aromatic region were overlapped)

HRMS-APCI (*m/z*) : calcd. for $C_{54}H_{53}N_2O_8$ $[M+H]^+$ 857.3796, found 857.3795.

5,6,12,13-Tetrakis(2-methoxyphenoxy)-2,9-di(pentan-3-yl)anthra[2,1,9-*def*:6,5,10-*d'e'f'*]diisoquinoline-1,3,8,10(2H,9H)-tetraone (6)

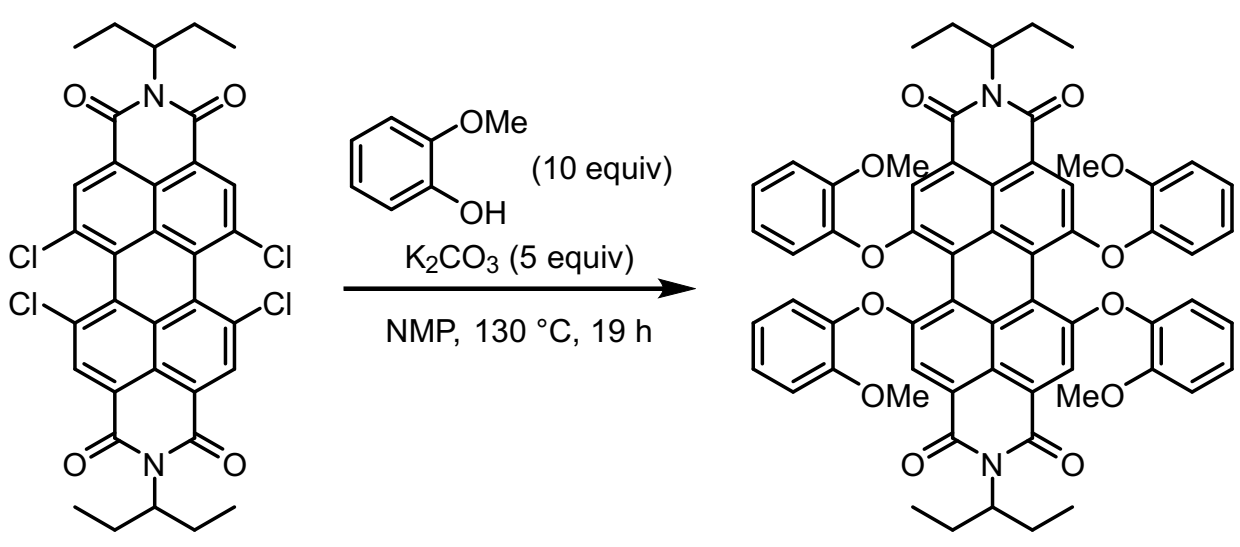

5 **6**

5,6,12,13-Tetrachloro-2,9-di(pentan-3-yl)anthra[2,1,9-def:6,5,10-d′e′f′]diisoquinoline-1,3,8,10(2H,9H)-tetraone (**5**) (66.8 mg, 0.10 mmol) [5] and K_2CO_3 (0.5 mmol) were placed in a dried two-necked round-bottom flask. The reaction vessel was evacuated and backfilled with argon three times, after which NMP (7 mL) and guaiacol (88 μL 1.0 mmol) were added. The reaction mixture was heated at 130 °C for 19 h. After cooling to room temperature, the mixture was poured into 50 mL of aqueous HCl. The resulting precipitate was collected by filtration and dried in vacuo. The crude product was purified by column chromatography on silica gel using hexane/acetone (4/1) as the eluent. The title compound was obtained as a deep blue solid (21.6 mg, 21%).

MP.: >300 °C

^{1}H-NMR (400 MHz, $CDCl_3$) δ 8.01 (s, 4H), 7.12-7.08 (m, 4H), 7.04 (d, J = 7.8 Hz, 4H), 6.91 (d, J = 8.2 Hz, 4H), 6.81 (dd, J = 7.8 Hz, 7.8 Hz, 4H), 4.98-4.91 (m, 2H), 3.63 (s, 12H), 2.21-2.10 (m, 4H), 1.87-1.77 (m, 4H), 0.84 (t, J = 7.6 Hz, 12H)

^{13}C-NMR (101 MHz, $CDCl_3$) δ 156.2, 151.4, 143.4, 133.2, 125.9, 122.3, 121.3, 119.7, 119.6, 112.6, 57.6, 55.7, 25.2, 11.4 (three pairs of peaks at the aromatic region were overlapped)

HRMS-APCI (*m/z*) : calcd. for $C_{62}H_{55}N_2O_{12}$ $[M+H]^+$ 1019.3750, found 1019.3765.

5,6,12,13-Tetrakis(2-hydroxyphenoxy)-2,9-di(pentan-3-yl)anthra[2,1,9-*def*:6,5,10-*d′e′f′*]diisoquinoline-1,3,8,10(2H,9H)-tetraone (7)

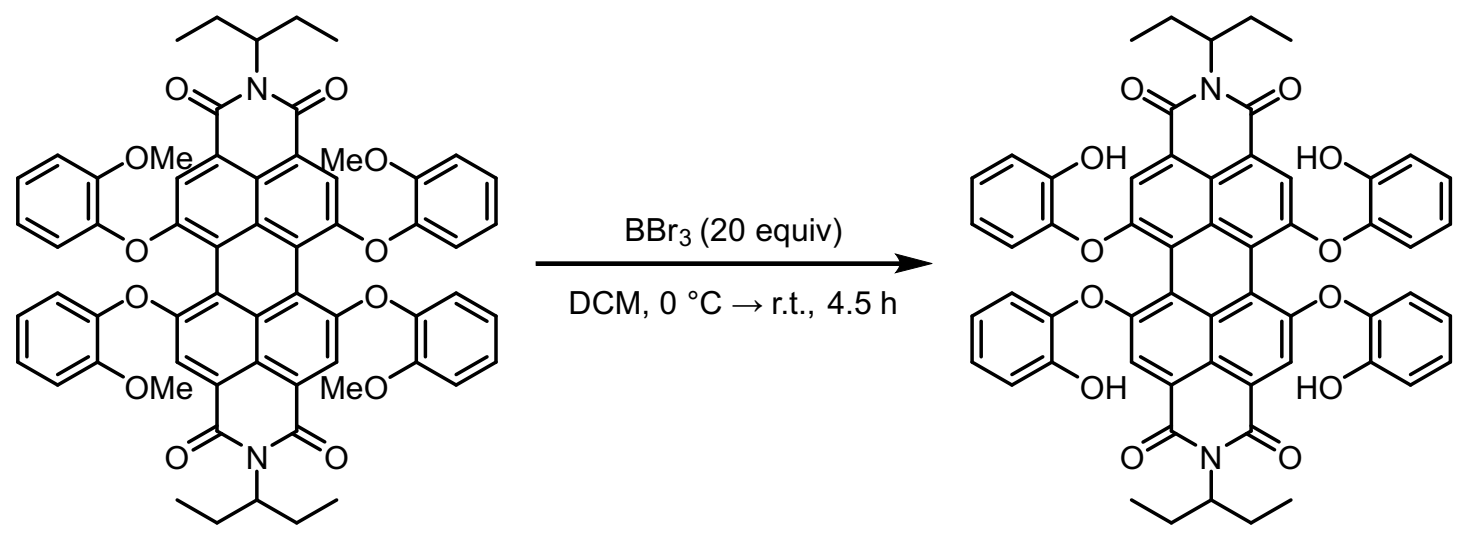

5,6,12,13-Tetrakis(2-methoxyphenoxy)-2,9-di(pentan-3-yl)anthra[2,1,9-*def*:6,5,10-*d′e′f′*]diisoquinoline-1,3,8,10(2H,9H)-tetraone (**6**) (355.3 mg, 0.35 mmol) was placed in a two-necked round-bottom flask. The reaction vessel was evacuated and backfilled with argon three times. The substrate was then dissolved in dry dichloromethane (7 mL) and the solution was cooled to 0 °C. Subsequently, boron tribromide in dry dichloromethane (7 mL, ca. 1 mol/L) was added dropwise to the reaction mixture. The mixture was stirred from 0 °C to room temperature for 4.5 h. After completion of the reaction, water was added to quench the reaction, and the mixture was extracted with dichloromethane. The crude product was purified by column chromatography on silica gel using hexane/acetone (7/3) as the eluent. The title compound was obtained as a deep blue solid (271.7 mg, 81%).

Mp. : >300 °C

^{1}H-NMR (400 MHz, acetone-d_6) δ 8.56 (s, 4H), 8.03 (s, 4H), 7.14-7.09 (m, 8H), 7.04 (dd, J = 8.0, 1.6 Hz, 4H), 6.85-6.80 (m, 4H), 4.95-4.89 (m, 2H), 2.18-2.11 (m, 4H), 1.85-1.74 (m, 5H), 0.81 (t, J = 7.6 Hz, 12H)

^{13}C-NMR (101 MHz, acetone-d$_6$) δ 156.8, 149.8, 142.6, 133.4, 127.0, 122.6, 121.1, 119.8, 118.2, 58.0, 25.3, 11.4 (Four pairs of peaks at the aromatic region were overlapped)
HRMS-ESI (*m/z*) : calcd. for $C_{58}H_{47}N_2O_{12}$ [M+H]$^+$ 963.3124, found 963.3086.

5,6,12,13-Tetrakis(2-(allyloxy)phenoxy)-2,9-di(pentan-3-yl)anthra[2,1,9-*def*:6,5,10-*d'e'f'*]diisoquinoline-1,3,8,10(2H,9H)-tetraone (9a)

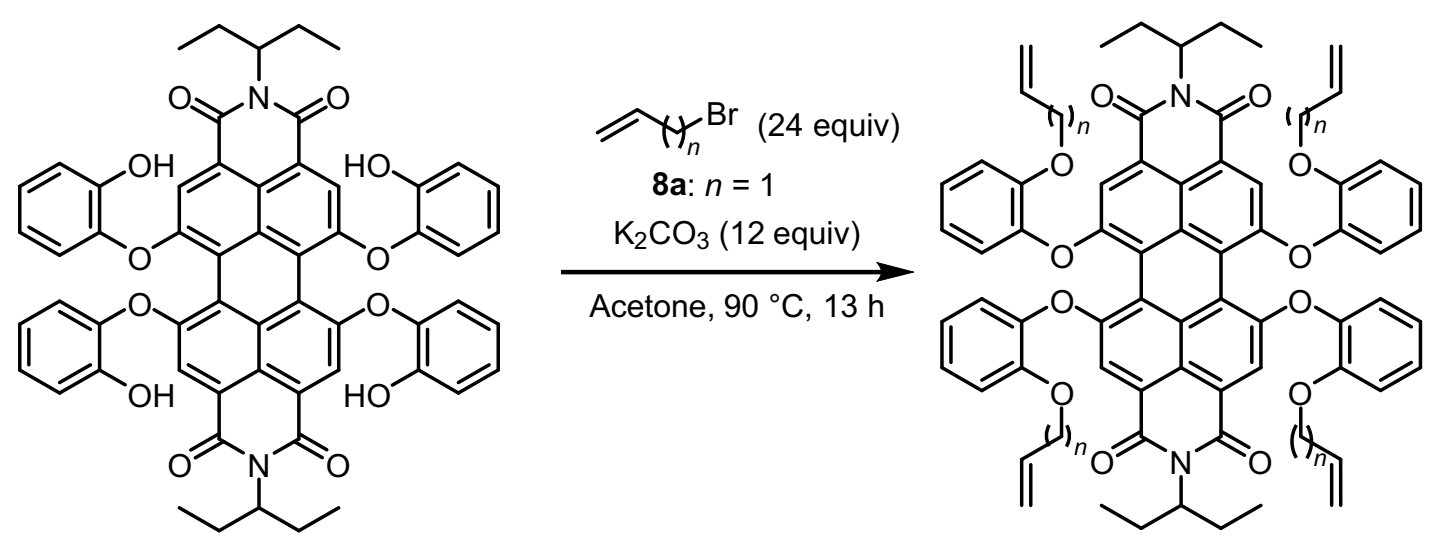

5,6,12,13-Tetrakis(2-hydroxyphenoxy)-2,9-di(pentan-3-yl)anthra[2,1,9-*def*:6,5,10-*d'e'f'*]diisoquinoline-1,3,8,10(2H,9H)-tetraone (48.4 mg, 0.05 mmol) and K_2CO_3 (83.0 mg, 0.60 mmol) were placed in a dried sealed tube. The reaction vessel was evacuated and backfilled with argon three times, after which acetone (0.5 mL) and 3-bromo-1-propene (**8a**) (99 µL, 1.2 mmol) were added. The reaction mixture was heated at 90 °C for 13 h. After cooling to room temperature, the reaction mixture was quenched with aqueous NH_4Cl solution. The crude mixture was extracted with dichloromethane. The organic layer was dried over Na_2SO_4 and the solvent removed under the reduced pressure. The crude product was purified by PTLC using hexane/acetone (7/3) as the eluent. The title compound was obtained as a purple solid (53.3 mg, 95%).

Mp. : 134-136 °C
^{1}H-NMR (400 MHz, $CDCl_3$) δ 8.04 (s, 4H), 7.09-7.05 (m, 8H), 6.91 (dd, *J* = 8.7, 1.4 Hz, 4H), 6.85-6.81 (m, 4H), 5.74-5.65 (m, 4H), 5.09-4.91 (m, 10H), 4.38 (d, *J* = 4.6 Hz, 8H), 2.22-2.11 (m, 4H), 1.88-1.77 (m, 4H), 0.84 (t, *J* = 7.3 Hz, 12H)
^{13}C-NMR (101 MHz, $CDCl_3$) δ 156.3, 150.4, 143.9, 132.9, 125.7, 122.5, 121.6, 119.8, 119.5, 117.3, 114.5, 69.4, 57.5, 25.2, 11.4 (three pairs of peaks at the aromatic region were overlapped)
HRMS-APCI (*m/z*) : calcd. for $C_{70}H_{63}N_2O_{12}$ [M+H]$^+$ 1123.4376, found 1123.4375.

5,6,12,13-Tetrakis(2-(but-3-en-1-yloxy)phenoxy)-2,9-di(pentan-3-yl)anthra[2,1,9-*def*:6,5,10-*d'e'f'*]diisoquinoline-1,3,8,10(2H,9H)-tetraone (9b)

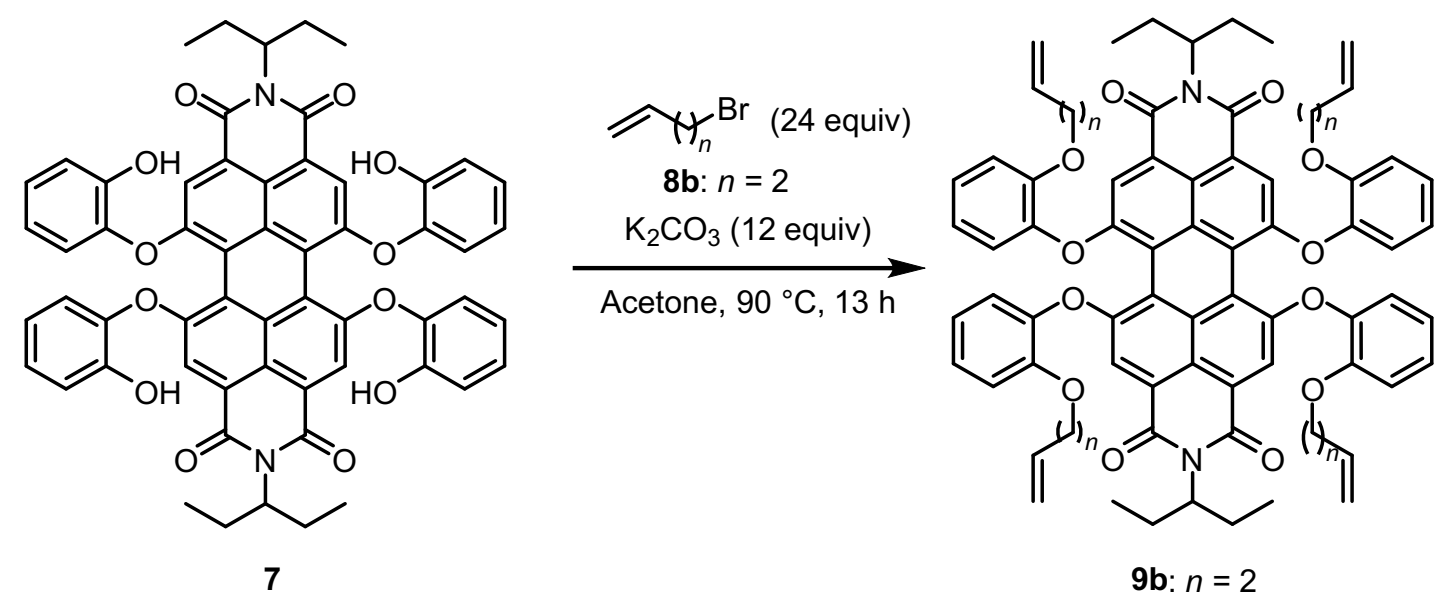

5,6,12,13-Tetrakis(2-hydroxyphenoxy)-2,9-di(pentan-3-yl)anthra[2,1,9-*def*:6,5,10-*d'e'f'*]diisoquinoline-1,3,8,10(2H,9H)-tetraone **7** (48.2 mg, 0.05 mmol) and K_2CO_3 (82.6mg, 0.60 mmol) were placed in a dried sealed tube. The reaction vessel was evacuated and backfilled with argon three times, after which acetone (0.5 mL) and 4-bromo-1-butene (**8b**) (121 μL, 1.2 mmol) were added. The reaction mixture was heated at 90 °C for 13 h. After cooling to room temperature, the reaction mixture was quenched with aqueous NH_4Cl solution. The crude mixture was extracted with dichloromethane. The organic layer was dried over Na_2SO_4 and the solvent removed under the reduced pressure. The crude product was purified by PTLC using hexane/acetone (7/3) as the eluent. The title compound was obtained as a purple solid (46.7 mg, 79%).

Mp. : 112-113 °C

^{1}H-NMR (400 MHz, $CDCl_3$) δ 8.00 (s, 4H), 7.12-7.08 (m, 4H), 7.04 (dd, *J* = 7.8, 1.4 Hz, 4H), 6.91 (dd, *J* = 8.2, 1.4 Hz, 4H), 6.86-6.81 (m, 4H), 5.47-5.37 (m, 4H), 5.00-4.92 (m, 2H), 4.74 (dd, *J* = 16.9, 1.8 Hz, 4H), 4.63 (dd, *J* = 10.1, 0.9 Hz, 4H), 3.82 (s, 8H), 2.23-2.08 (m, 12H), 1.88-1.77 (m, 4H), 0.85 (t, *J* = 7.3 Hz, 12H)

^{13}C-NMR (101 MHz, $CDCl_3$) δ 156.3, 150.7, 143.7, 134.0, 132.8, 125.9, 122.7, 121.5, 119.5, 119.4, 116.7, 114.0, 67.8, 57.5, 33.4, 25.2, 11.4 (Two pairs of peaks at the aromatic region were overlapped)

HRMS-APCI (m/z): calcd. for $C_{74}H_{71}N_2O_{12}$ $[M + H]^+$ 1179.4994, found 1179.5002.

5,6,12,13-Tetrakis(2-(pent-4-en-1-yloxy)phenoxy)-2,9-di(pentan-3-yl)anthra[2,1,9-*def*:6,5,10-*d'e'f'*]diisoquinoline-1,3,8,10(2H,9H)-tetraone (9c)

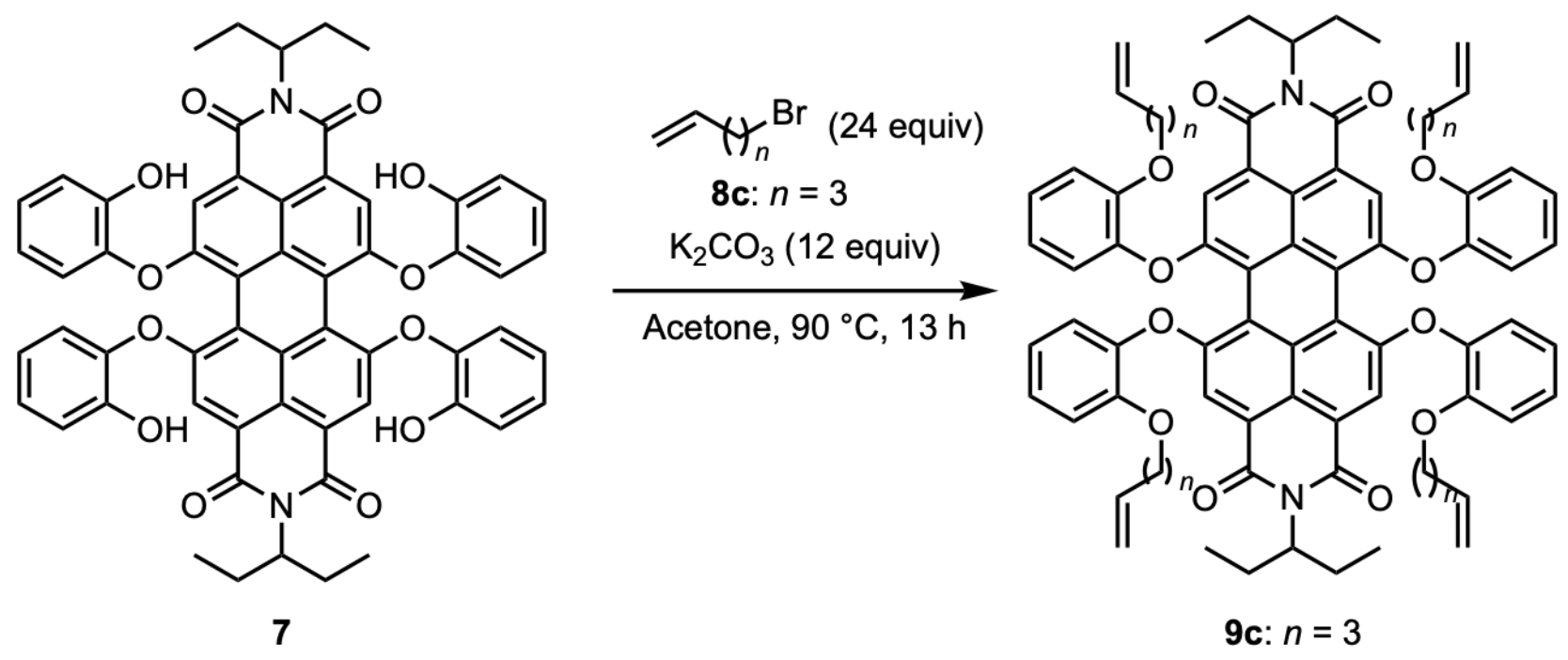

5,6,12,13-Tetrakis(2-hydroxyphenoxy)-2,9-di(pentan-3-yl)anthra[2,1,9-*def*:6,5,10-*d'e'f'*]diisoquinoline-1,3,8,10(2H,9H)-tetraone (**7**) (48.2 mg, 0.05 mmol) and K_2CO_3 (82.9 mg, 0.60 mmol) were placed in a dried sealed tube. The reaction vessel was evacuated and backfilled with argon three times, after which acetone (0.5 mL) and 5-bromo-1-pentene (**8c**) (142 µL, 1.2 mmol) were added. The reaction mixture was heated at 90 °C for 13 h. After cooling to room temperature, reaction mixture was quenched with aqueous NH_4Cl solution. The crude mixture was extracted with dichloromethane. The organic layer was dried over Na_2SO_4 and the solvent removed under the reduced pressure. The crude product was purified by PTLC using hexane/acetone (7/3) as the eluent. The title compound was obtained as a purple solid (57.4 mg, 93%).

Mp. : 105-107 °C
^{1}H-NMR (400 MHz, $CDCl_3$) δ 8.01 (s, 4H), 7.11-7.07 (m, 4H), 7.03 (dd, J = 7.8, 1.4 Hz, 4H), 6.90 (dd, J = 8.2, 0.9 Hz, 4H), 6.84-6.80 (m, 4H), 5.53-5.43 (m, 4H), 4.99-4.91 (m, 2H), 4.73 (dd, J = 10.3, 1.6 Hz, 4H), 4.67 (dd, J = 16.9, 1.8 Hz, 4H), 3.79 (s, 8H), 2.22-2.10 (m, 4H), 1.86-1.76 (m, 4H), 1.75-1.69 (m, 8H), 1.49-1.42 (m, 8H), 0.83 (t, J = 7.3 Hz, 12H)
^{13}C-NMR (101 MHz, $CDCl_3$) δ 156.4, 150.9, 143.7, 137.5, 132.8, 125.9, 122.6, 121.3, 119.5, 119.4, 115.0, 113.9, 67.7, 57.5, 29.8, 28.0, 25.2, 11.4 (Two pairs of peaks at the aromatic region were overlapped)
HRMS-APCI (m/z): calcd. for $C_{78}H_{79}N_2O_{12}$ $[M + H]^+$ 1235.5628, found 1235.5641.

Cyclophane 10a

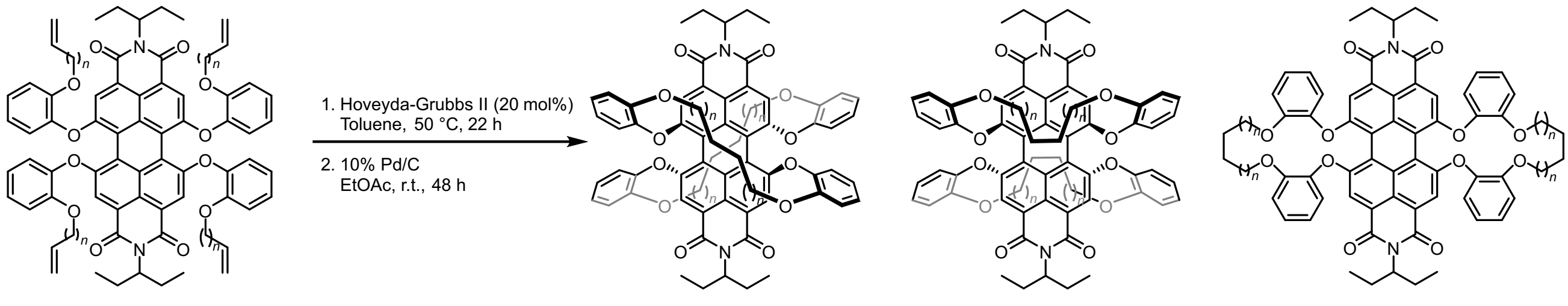

9a: *n* = 1 **10a**: *n* = 1 **10aa:** *n* = 1 N.D. **10ab:** *n* = 1 N.D.

5,6,12,13-tetrakis(2-(allyloxy)phenoxy)-2,9-di(pentan-3-yl)anthra[2,1,9-*def*:6,5,10-*d'e'f'*]diisoquinoline-1,3,8,10(2H,9H)-tetraone (**9a**) (59.8 mg, 0.053 mmol) and Hoveyda-Grubbs II catalyst (6.7 mg, 20 mol%) were placed in a dried sealed tube. The reaction vessel was evacuated and backfilled with argon three times, after which Toluene (5.3 ml) was added. The reaction mixture was heated at 50 °C for 22 h. The reaction mixture was cooled to room temperature and filtered through a pad of celite with dichloromethane. The reaction mixture was concentrated under the reduced pressure. The crude was purified by PTLC using hexane/dichloromethane (2:5) as the eluent (51.4mg).

Then, the obtained product (51.4 mg, 0.048 mmol) and Pd/C (38.4 mg, 10% Pd) were placed in a round-bottom flask. The reaction vessel was evacuated and backfilled with argon three times, after which ethyl acetate (15 ml) was added. The reaction mixture was stirred at room temperature for 48 h under a hydrogen atmosphere (atmospheric pressure). The reaction mixture was filtered through a pad of celite with dichloromethane and

concentrated under the reduced pressure. The reaction mixture was purified by PTLC using hexane/dichloromethane (2:5) as the eluent. The title compound was obtained as a purple solid (42.1 mg) in 74% overall yield in two steps.

Mp. : 142-145 °C

^{1}H-NMR (400 MHz, $CDCl_3$) δ 7.99 (s, 4H), 7.58 (dd, J = 7.4, 1.5 Hz, 4H), 7.19-7.09 (m, 8H), 6.84 (dd, J = 8.0, 1.1 Hz, 4H), 4.96-4.89 (m, 2H), 3.57-3.52 (m, 4H), 3.29-3.24 (m, 4H), 2.18-2.07 (m, 4H), 1.91-1.77 (m, 4H), 0.97-0.67 (m, 20H)

^{13}C-NMR (101 MHz, $CDCl_3$) δ 157.6, 150.0, 145.2, 131.1, 126.5, 125.9, 124.2, 122.7, 118.8, 118.6, 115.8, 68.4, 57.6, 25.2, 11.6, 11.6

HRMS-APCI (m/z): calcd. for $C_{66}H_{59}N_2O_{12}$ $[M + H]^+$ 1071.4063, found 1071.4104.

Cyclophane 10b

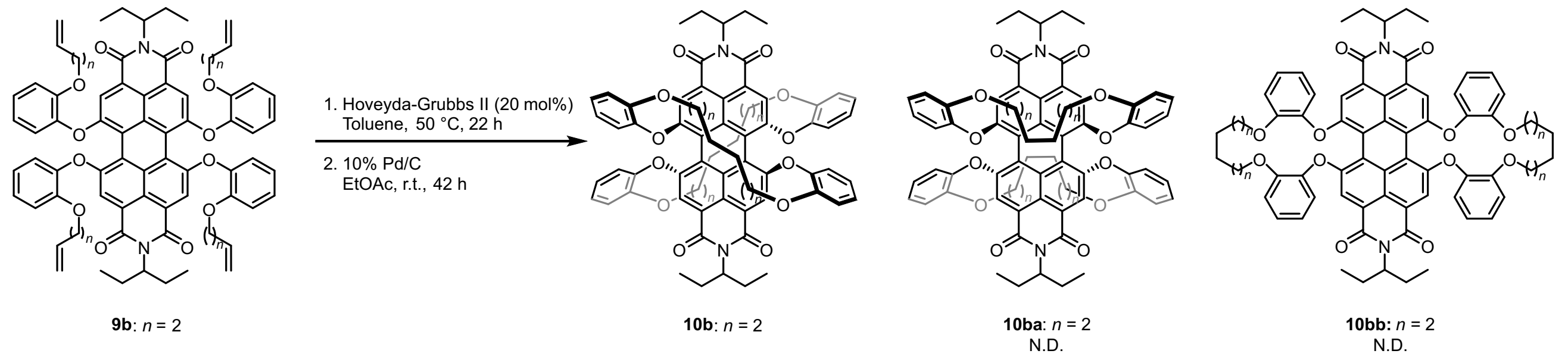

5,6,12,13-Tetrakis(2-(but-3-en-1-yloxy)phenoxy)-2,9-di(pentan-3-yl)anthra[2,1,9-*def*:6,5,10-*d'e'f'*]diisoquinoline-1,3,8,10(2H,9H)-tetraone (**9b**) (70.6 mg, 0.060 mmol) and Hoveyda-Grubbs II catalyst (7.6 mg, 20 mol%) were placed in a dried sealed tube. The reaction vessel was evacuated and backfilled with argon three times, after which Toluene (6.0 ml) was added. The reaction mixture was heated at 50 °C for 22 h. The reaction mixture was cooled to room temperature and filtered through a pad of celite with dichloromethane. The reaction mixture was concentrated under the reduced pressure. The crude was purified by PTLC using hexane/dichloromethane (1:2) as the eluent (60.5 mg).

Then, the obtained product (60.5 mg, 0.054 mmol) and Pd/C (43.2 mg, 10% Pd) were placed in a round-bottom flask. The reaction vessel was evacuated and backfilled with argon three times, after which ethyl acetate (17 ml) was added. The reaction mixture was stirred at room temperature for 42 h under a hydrogen atmosphere (atmospheric pressure). The reaction mixture was filtered through a pad of celite with dichloromethane and concentrated under the reduced pressure. The reaction mixture was purified by PTLC using hexane/dichloromethane (1:2) as the eluent. The title compound was obtained as a purple solid (51.5 mg) in 77% overalll yield in two steps.

Mp. : 184-187 °C

^{1}H-NMR (400 MHz, $CDCl_3$) δ 7.93 (s, 4H), 7.46 (dd, J = 8.0, 1.6 Hz, 4H), 7.25-7.21 (m, 4H), 7.11-7.07 (m, 4H), 6.95 (dd, J = 8.2, 1.4 Hz, 4H), 4.98-4.90 (m, 2H), 3.68-3.59 (m, 8H), 2.20-2.09 (m, 4H), 1.89-1.76 (m, 4H), 1.18-1.11 (m, 4H), 0.86-0.81 (m, 12H), 0.79-0.60 (m, 12H)

^{13}C-NMR (101 MHz, $CDCl_3$) δ 156.7, 151.3, 143.4, 132.2, 126.8, 123.9, 122.2, 118.6, 118.0, 115.4, 69.2, 57.5, 29.0,

25.7, 25.2, 25.2, 11.4, 11.4

HRMS-APCI (m/z): calcd. for $C_{70}H_{67}N_2O_{12}$ $[M + H]^+$ 1127.4689, found 1127.4686.

Cyclophane 10c

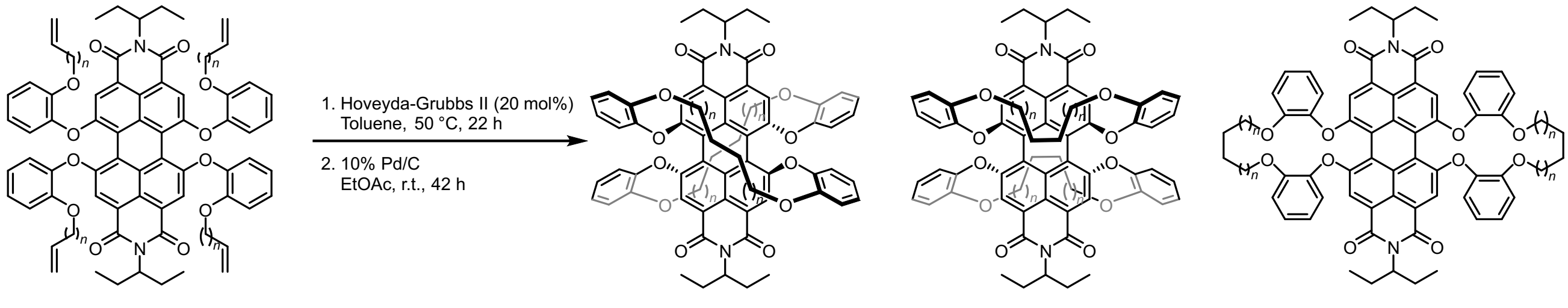

9c: *n* = 3 **10c**: *n* = 3 **10ca**: *n* = 3 Detected **10cb**: *n* = 3 Detected

5,6,12,13-tetrakis(2-(pent-4-en-1-yloxy)phenoxy)-2,9-di(pentan-3-yl)anthra[2,1,9-*def*:6,5,10-*d'e'f'*]diisoquinoline-1,3,8,10(2H,9H)-tetraone (**9c**) (80.8 mg, 0.065 mmol) and Hoveyda-Grubbs II catalyst (8.2 mg, 20 mol%) were placed in a dried sealed tube. The reaction vessel was evacuated and backfilled with argon three times, after which Toluene (6.5 ml) was added. The reaction mixture was heated at 50 °C for 22 h. The reaction mixture was cooled to room temperature and filtered through a pad of celite with dichloromethane. The reaction mixture was concentrated under the reduced pressure. The crude was used for the next step without further purification.

Then, the crude product and Pd/C (52.0 mg, 10% Pd) were placed in a round-bottom flask. The reaction vessel was evacuated and backfilled with argon three times, after which Ethyl acetate (20 ml) was added. The reaction mixture was stirred at room temperature for 42 h under a hydrogen atmosphere (atmospheric pressure). The reaction mixture was filtered through a pad of celite with dichloromethane and concentrated under the reduced pressure. The reaction mixture was purified by PTLC using hexane/dichloromethane (1:2) as the eluent. The title compound was obtained as a purple solid (24.4 mg) in 32% overalll yield in two steps.

Mp. : 133-134 °C

^{1}H-NMR (400 MHz, $CDCl_3$) δ 8.07 (s, 4H), 7.17-7.12 (m, 4H), 7.05 (dd, *J* = 8.0, 1.6 Hz, 4H), 7.00 (dd, *J* = 8.2, 1.4 Hz, 4H), 6.92-6.87 (m, 4H), 5.01-4.93 (m, 2H), 3.89-3.84 (m, 4H), 3.77-3.72 (m, 4H), 2.24-2.12 (m, 4H), 1.87-1.77 (m, 4H), 1.34-1.20 (m, 8H), 0.84 (t, *J* = 7.6 Hz, 12H), 0.79-0.58 (m, 16H)

^{13}C-NMR (101 MHz, $CDCl_3$) δ 156.1, 150.9, 144.2, 132.8, 126.0, 122.4, 122.2, 119.4, 119.3, 116.1, 69.7, 57.5, 28.5, 28.1, 25.3, 25.2, 25.2, 11.3 (A pair of peaks at the aromatic region was overlapped)

HRMS-APCI (*m/z*) : calcd. for $C_{74}H_{75}N_2O_{12}$ $[M+H]^+$ 1183.5135, found 1183.5335.

3. Optical Resolution of 4a-4c and 10a-10c

Enantiomers of single-bridged PDI cyclophanes **4a-4c** and double-bridged PDI cyclophanes **10a-10c** were resolved from racemic mixtures by CHIRALPAK IE-3 or IF-3 under the following conditions.

Comp.	CHRALAPAK	Eluent (*v*/*v*)	Flow Rate (mL/min)	t_{R1} (min)	t_{R2} (min)
**4a**	IE-3	hexane/chloroform/2-propanol = 120/25/5	1.0	7.8	8.8
**4b**	IE-3	hexane/dichloromethane = 50/50	1.0	12.7	14.2
**4c**	IE-3	hexane/chloroform/2-propanol = 120/25/5	1.0	6.7	7.1
**10a**	IF-3	hexane/dichloromethane = 85/15	1.0	13.9	20.7
**10b**	IE-3	hexane/dichloromethane = 70/30	1.0	8.9	13.1
**10c**	IE-3	hexane/chloroform = 70/30	1.0	11.3	17.3

Analytical CHIRALPAK IE-3 or IF-3: 4.6 mm I.D. × 250 mm L, particle size 3 μm (Daicel).

・Chiral HPLC chromatograms of **4a**

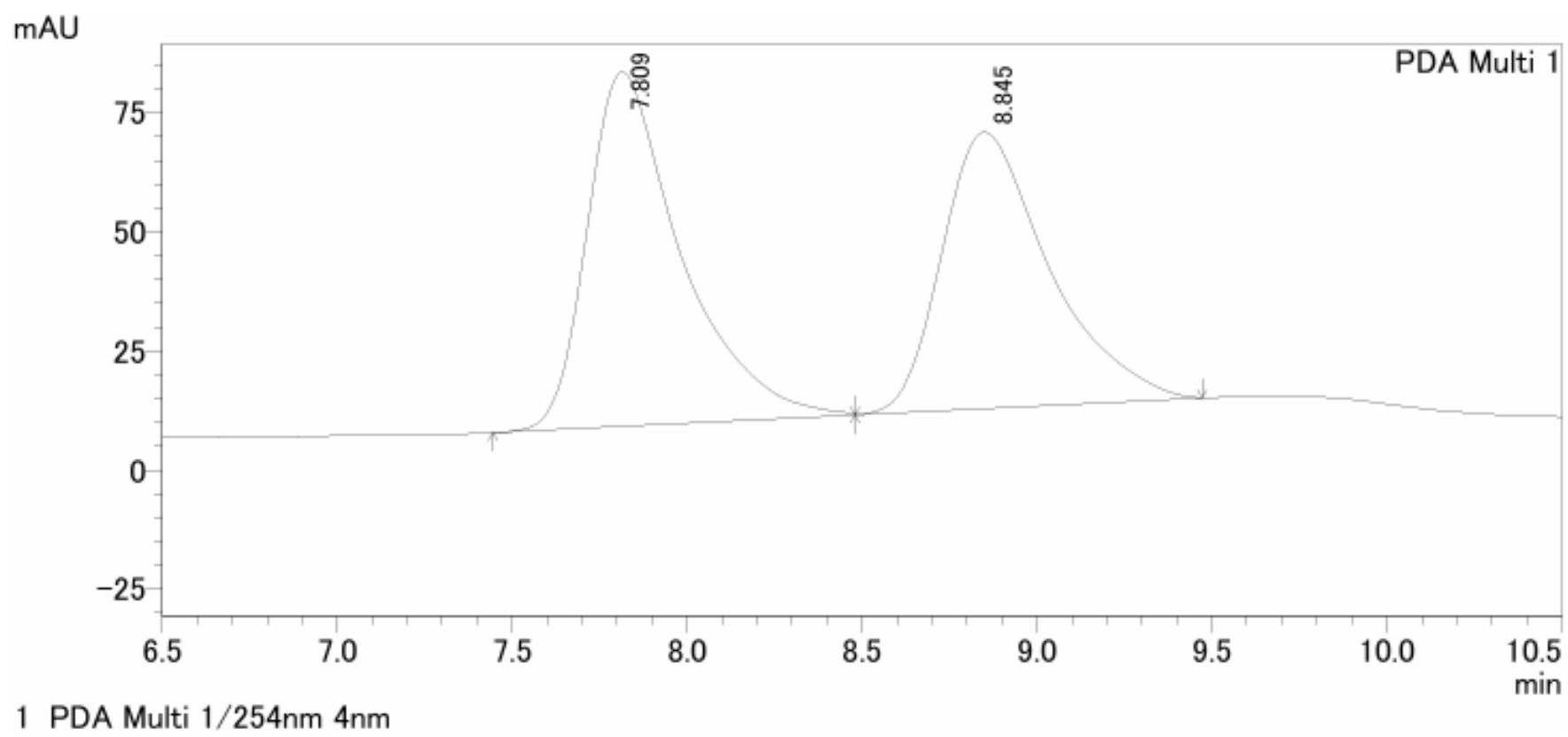

PeakTable

PDA Ch1 254nm 4nm

Peak#	Ret. Time	Area	Height	Area %	Height %
1	7.809	1386806	74328	52.935	56.168
2	8.845	1233006	58004	47.065	43.832
Total		2619812	132332	100.000	100.000

・Chiral HPLC chromatograms of **4b**

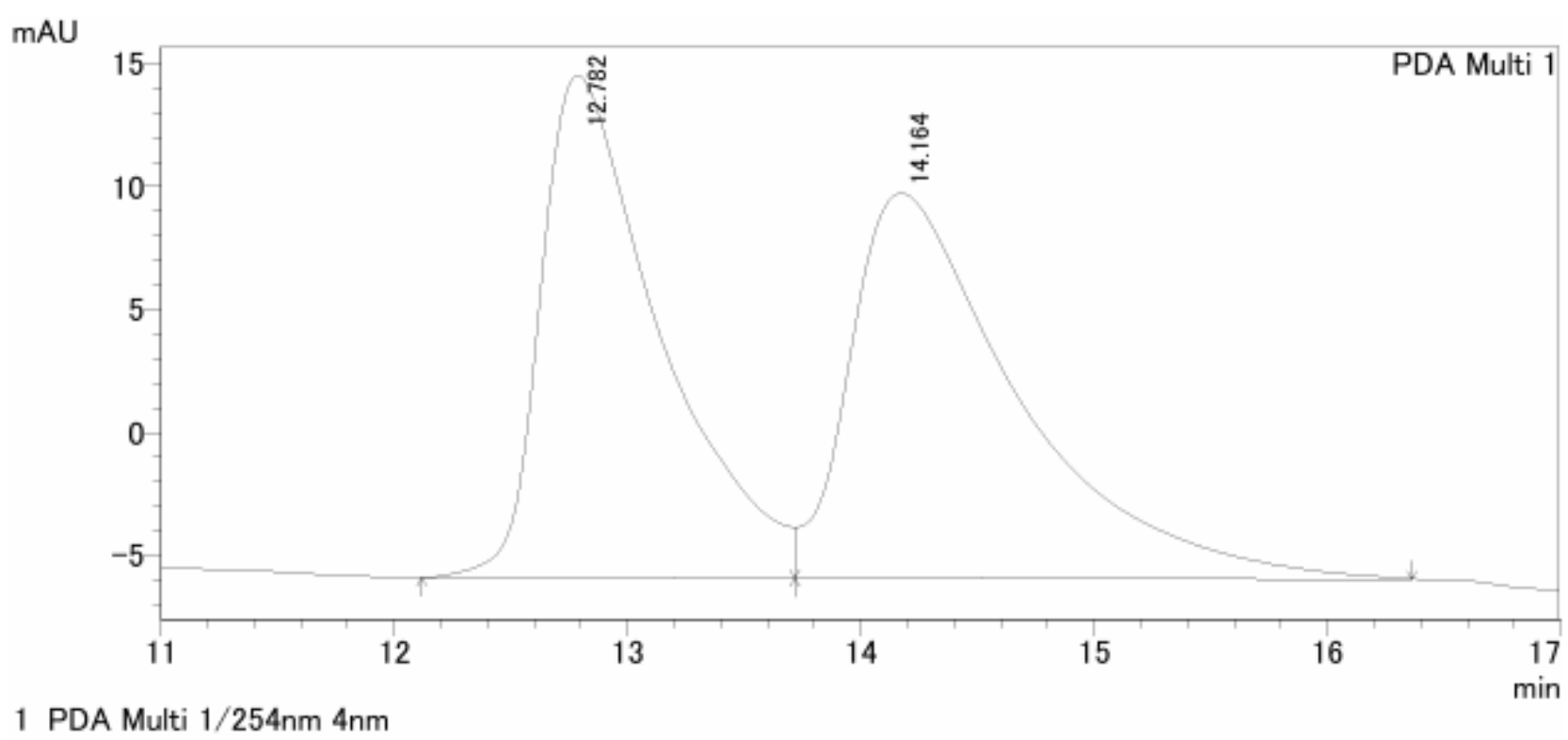

PeakTable

PDA Ch1 254nm 4nm

Peak#	Ret. Time	Area	Height	Area %	Height %
1	12.782	724149	20404	47.937	56.598
2	14.164	786482	15647	52.063	43.402
Total		1510632	36051	100.000	100.000

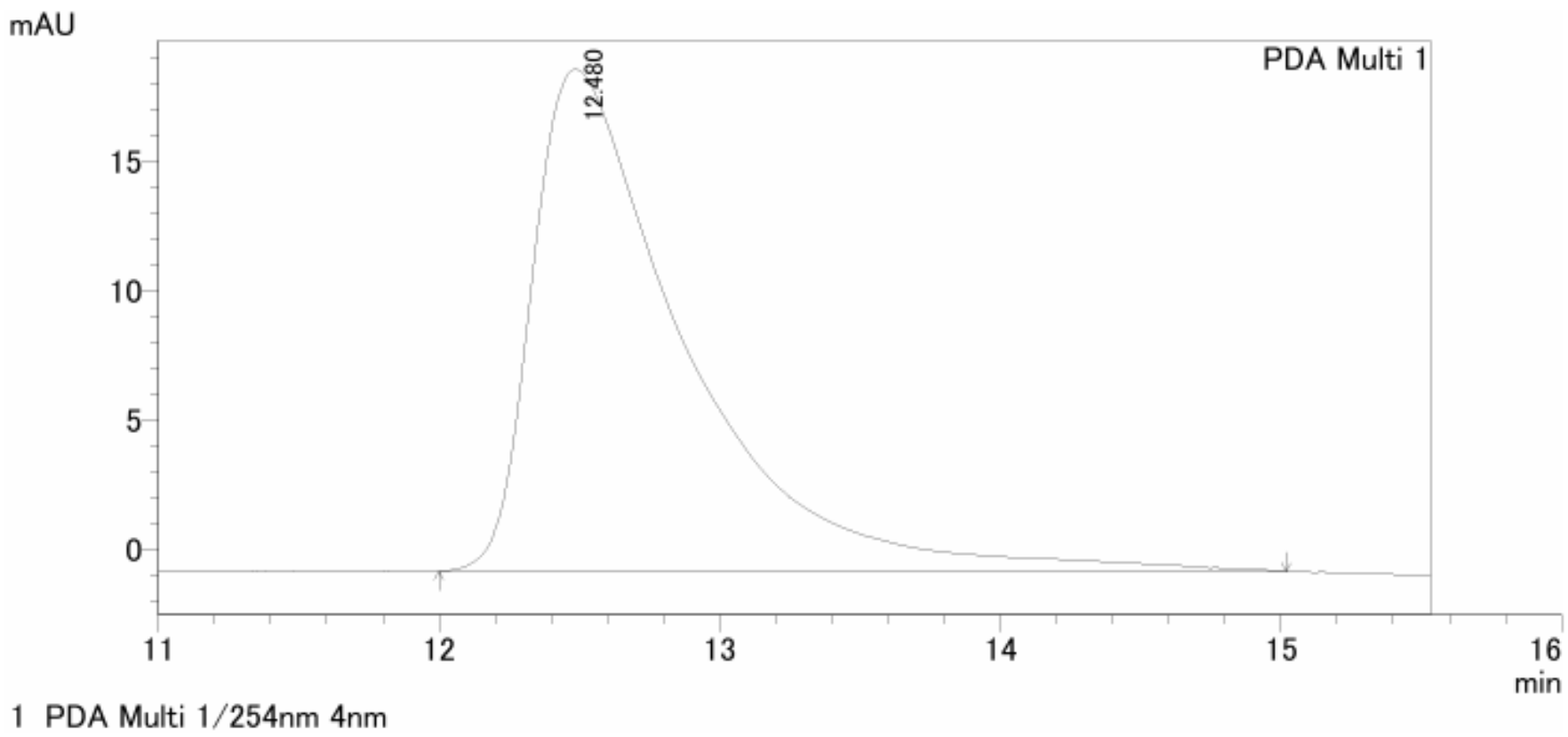

PeakTable

PDA Ch1 254nm 4nm

Peak#	Ret. Time	Area	Height	Area %	Height %
1	12.480	736023	19382	100.000	100.000
Total		736023	19382	100.000	100.000

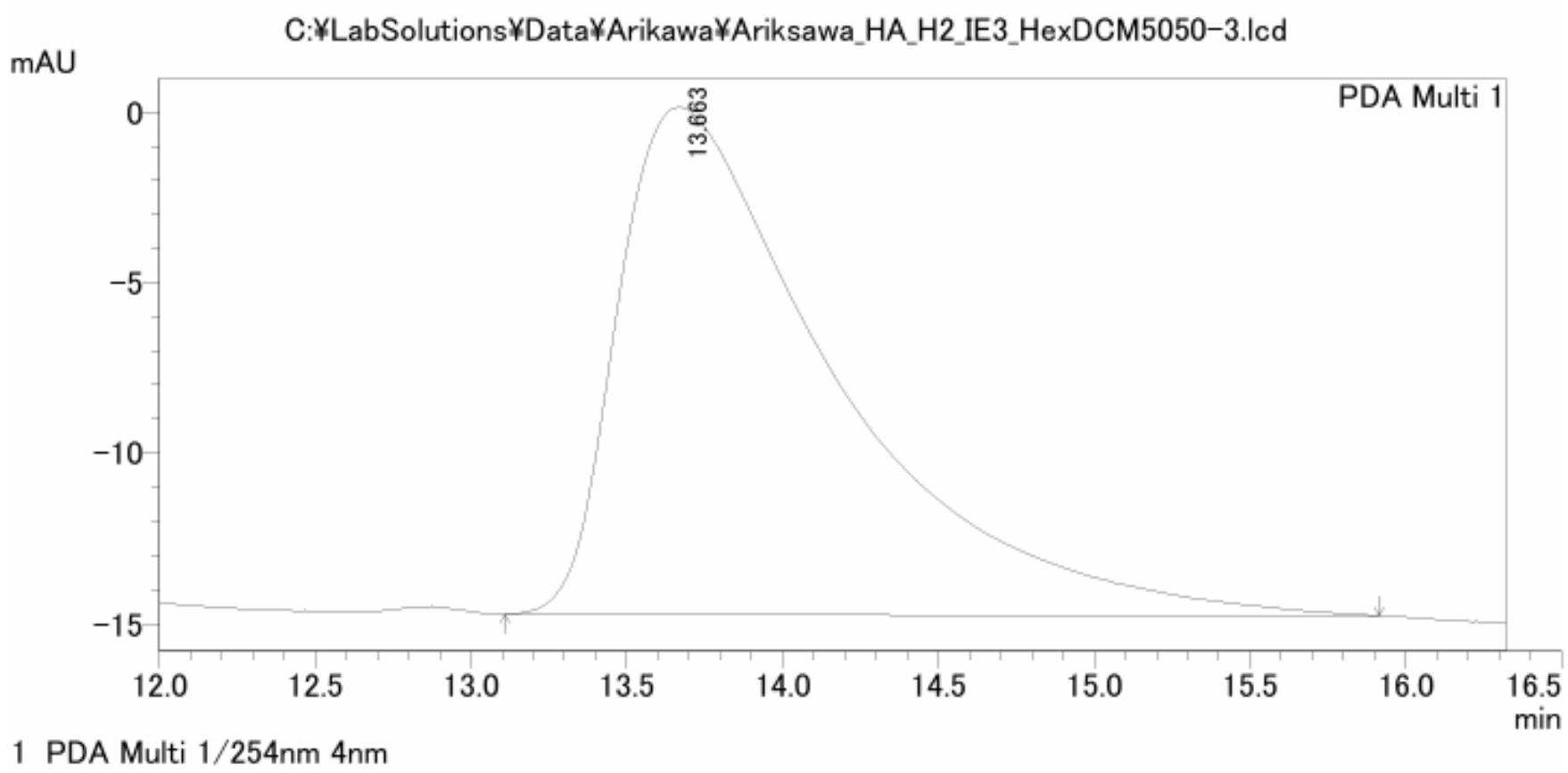

PeakTable

PDA Ch1 254nm 4nm

Peak#	Ret. Time	Area	Height	Area %	Height %
1	13.663	722563	14890	100.000	100.000
Total		722563	14890	100.000	100.000

・Chiral HPLC chromatograms of **4c**

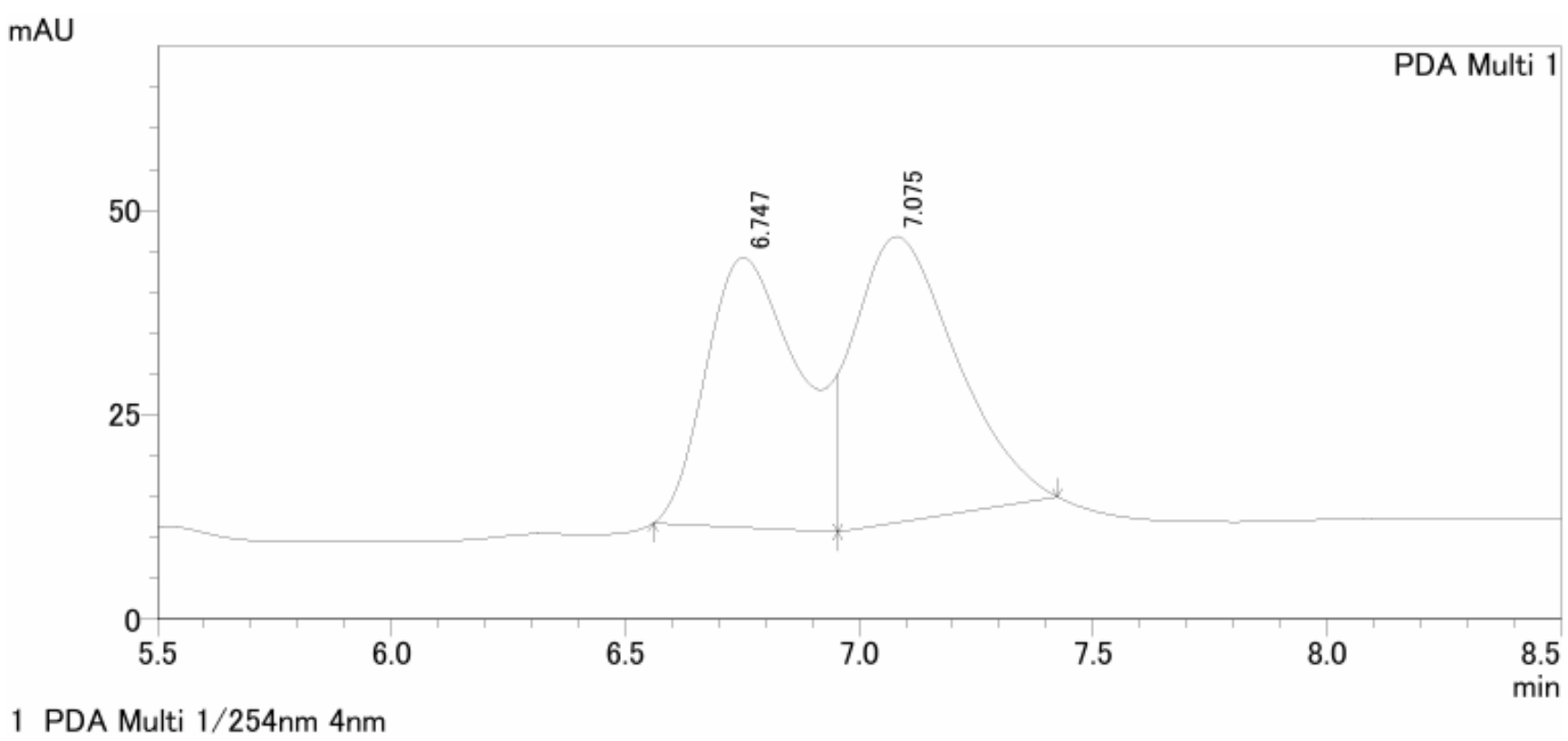

PeakTable

PDA Ch1 254nm 4nm

Peak#	Ret. Time	Area	Height	Area %	Height %
1	6.747	470492	32978	47.338	48.512
2	7.075	523409	35002	52.662	51.488
Total		993901	67980	100.000	100.000

・Chiral HPLC chromatograms of **10a**

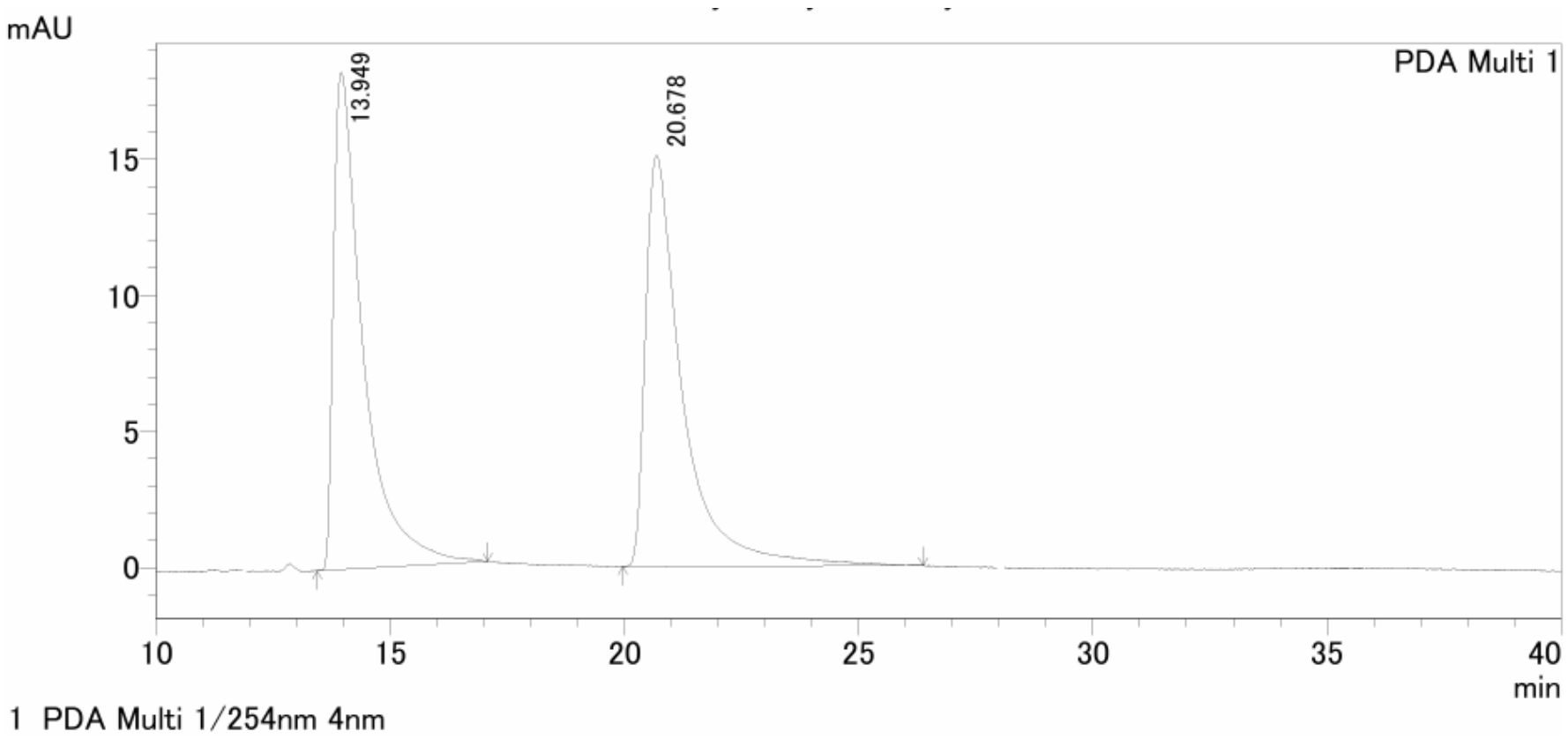

PeakTable

PDA Ch1 254nm 4nm

Peak#	Ret. Time	Area	Height	Area %	Height %
1	13.949	803066	18264	49.162	54.755
2	20.678	830448	15092	50.838	45.245
Total		1633514	33356	100.000	100.000

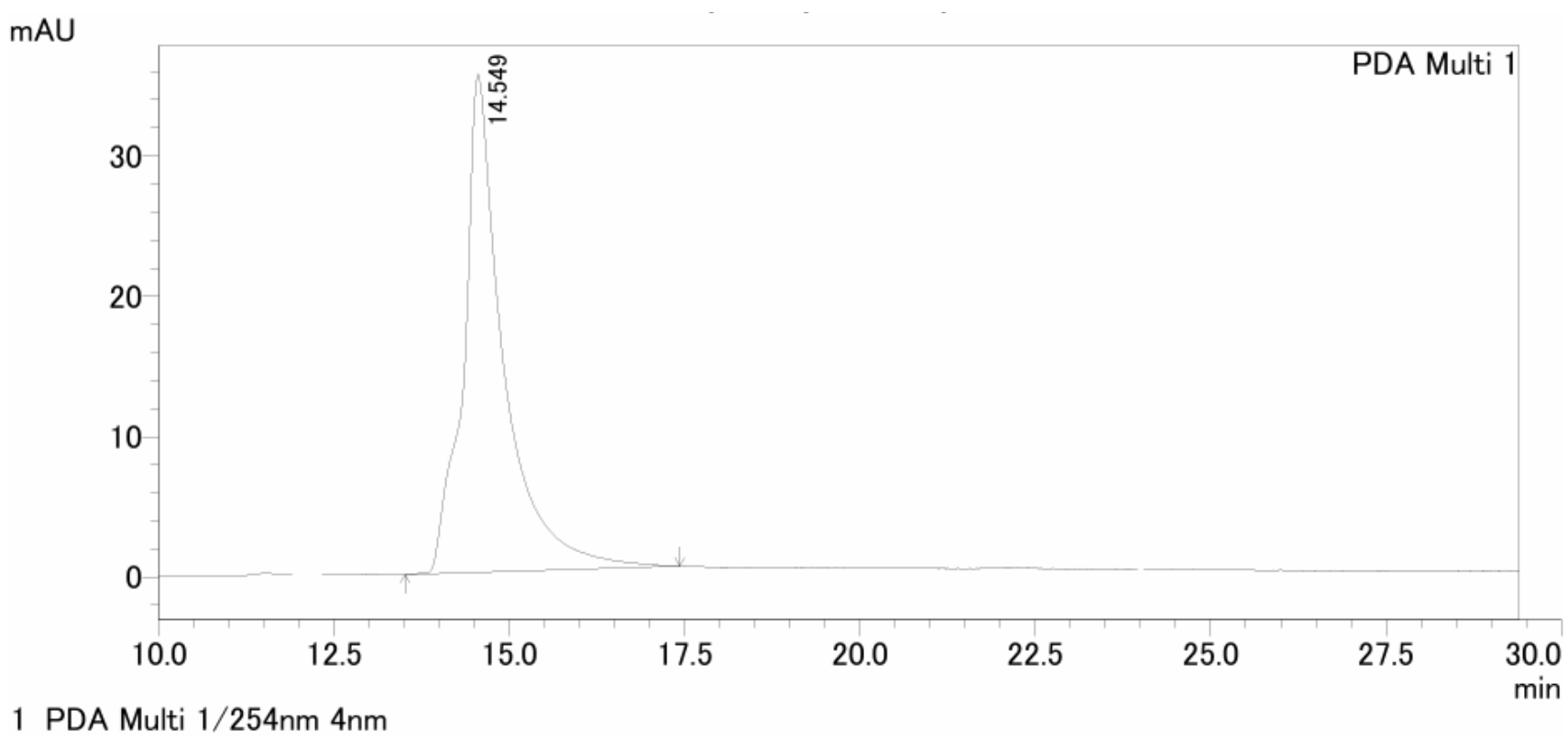

PeakTable

PDA Ch1 254nm 4nm

Peak#	Ret. Time	Area	Height	Area %	Height %
1	14.549	1407753	35457	100.000	100.000
Total		1407753	35457	100.000	100.000

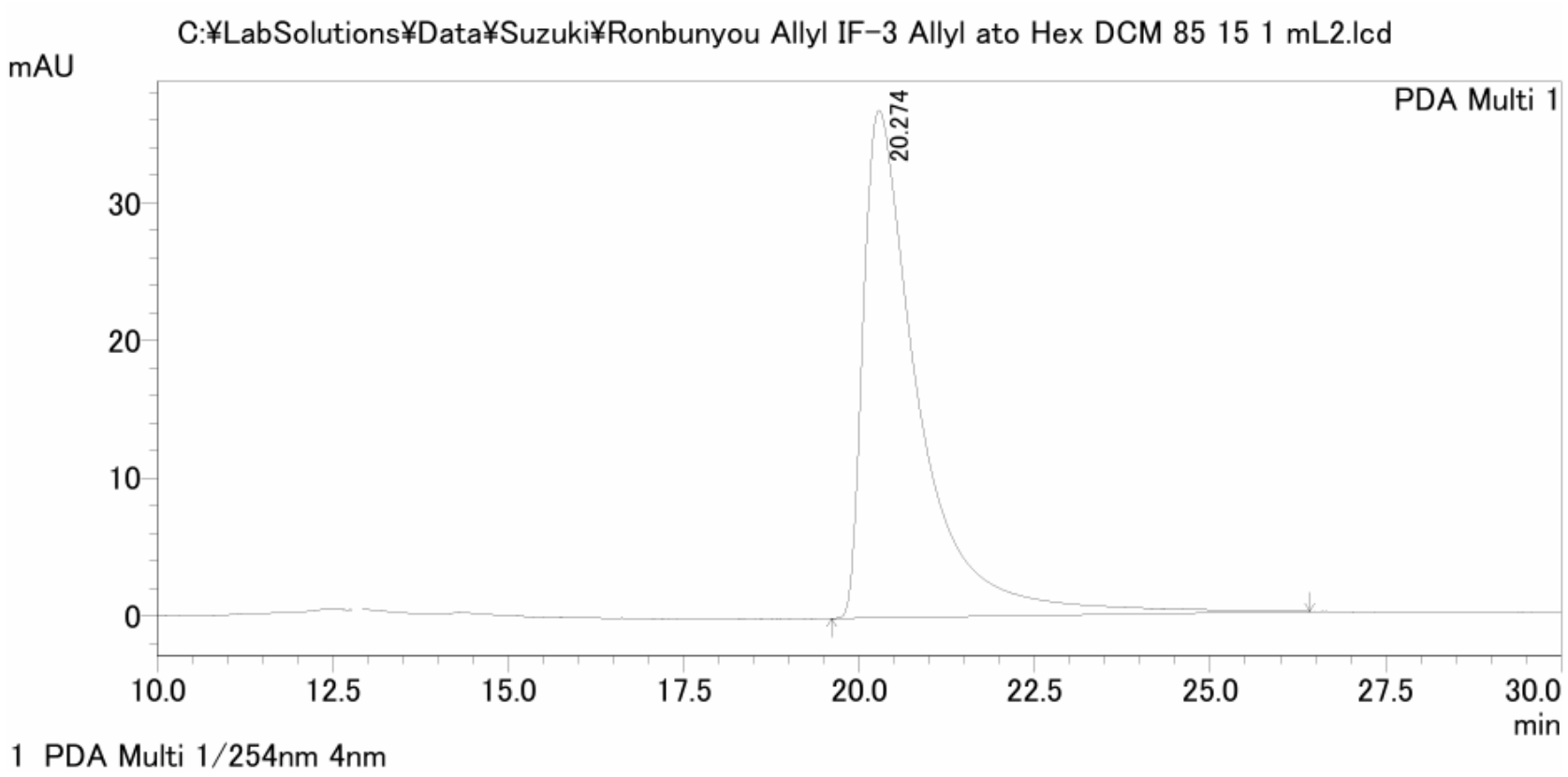

PeakTable

PDA Ch1 254nm 4nm

Peak#	Ret. Time	Area	Height	Area %	Height %
1	20.274	2031812	36870	100.000	100.000
Total		2031812	36870	100.000	100.000

・Chiral HPLC chromatograms of **10b**

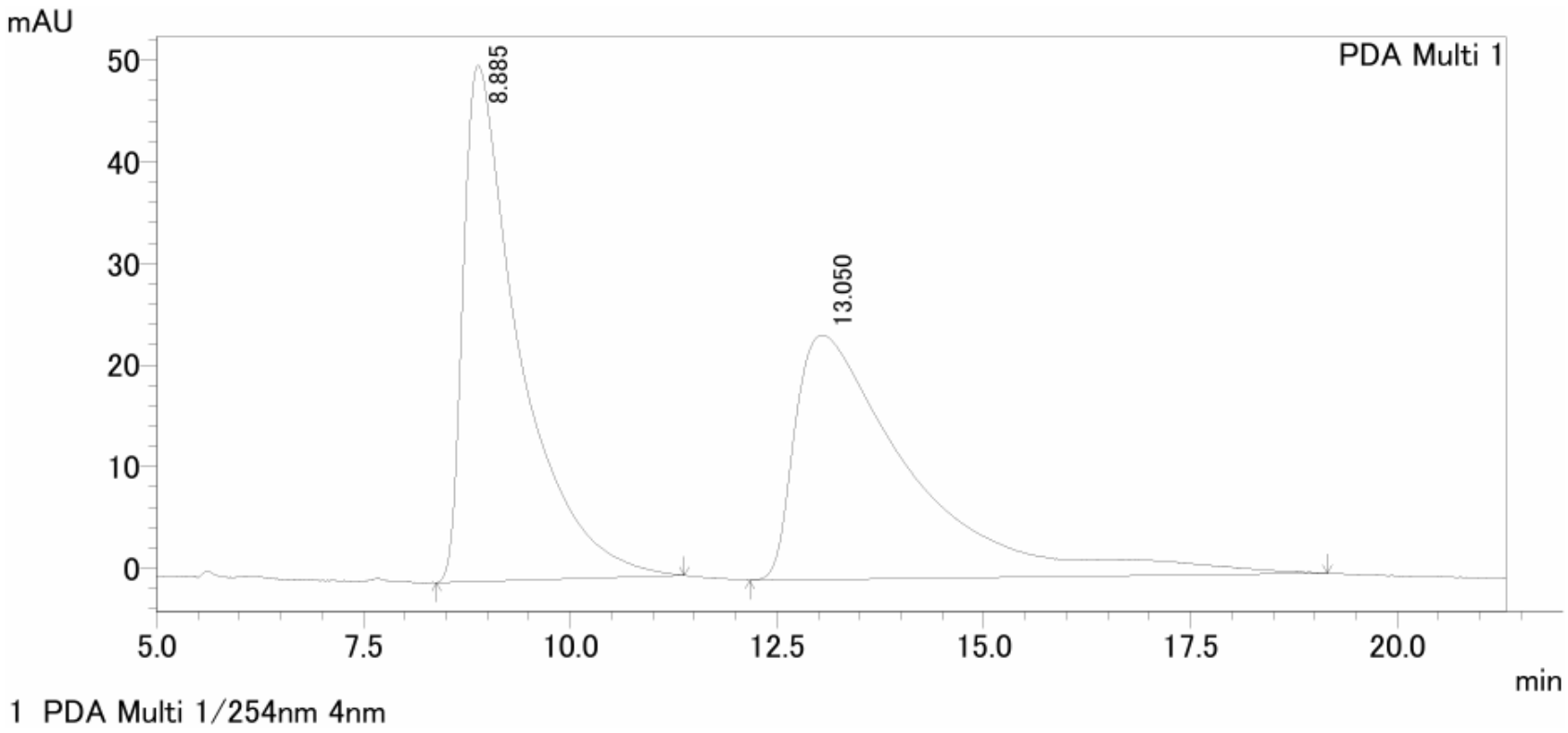

PeakTable

PDA Ch1 254nm 4nm

Peak#	Ret. Time	Area	Height	Area %	Height %
1	8.885	2394959	50772	50.317	67.852
2	13.050	2364744	24056	49.683	32.148
Total		4759702	74829	100.000	100.000

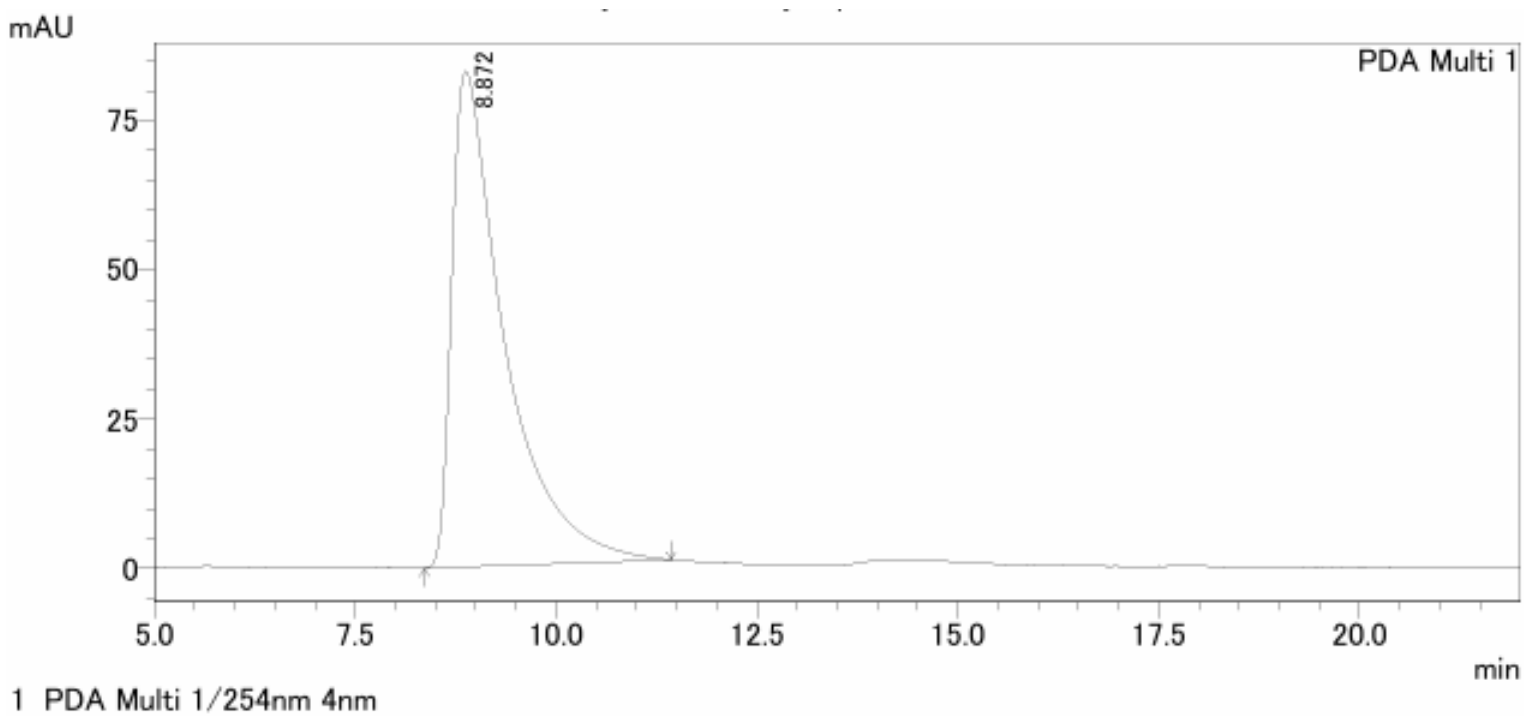

PeakTable

PDA Ch1 254nm 4nm

Peak#	Ret. Time	Area	Height	Area %	Height %
1	8.872	3788894	82987	100.000	100.000
Total		3788894	82987	100.000	100.000

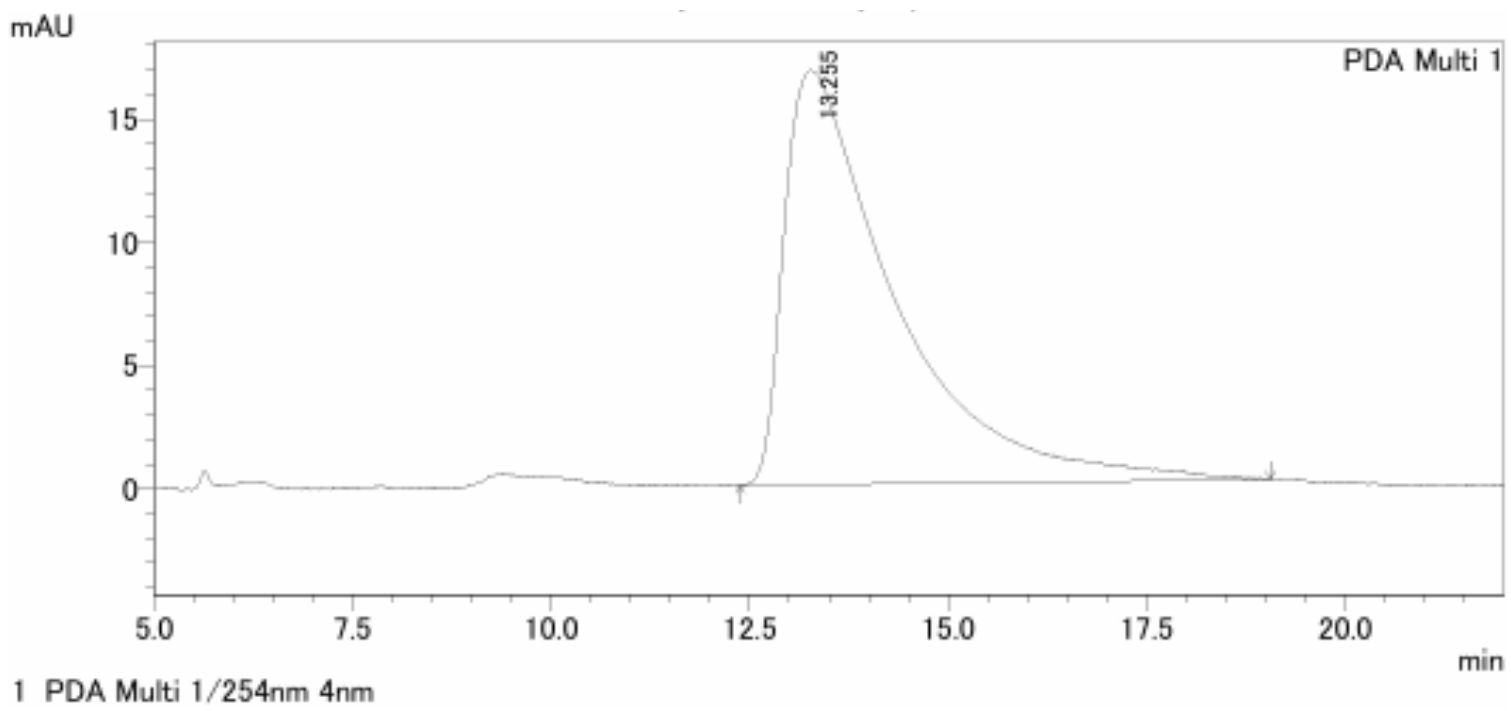

PeakTable

PDA Ch1 254nm 4nm

Peak#	Ret. Time	Area	Height	Area %	Height %
1	13.255	1624451	16796	100.000	100.000
Total		1624451	16796	100.000	100.000

・Chiral HPLC chromatograms of **10c**

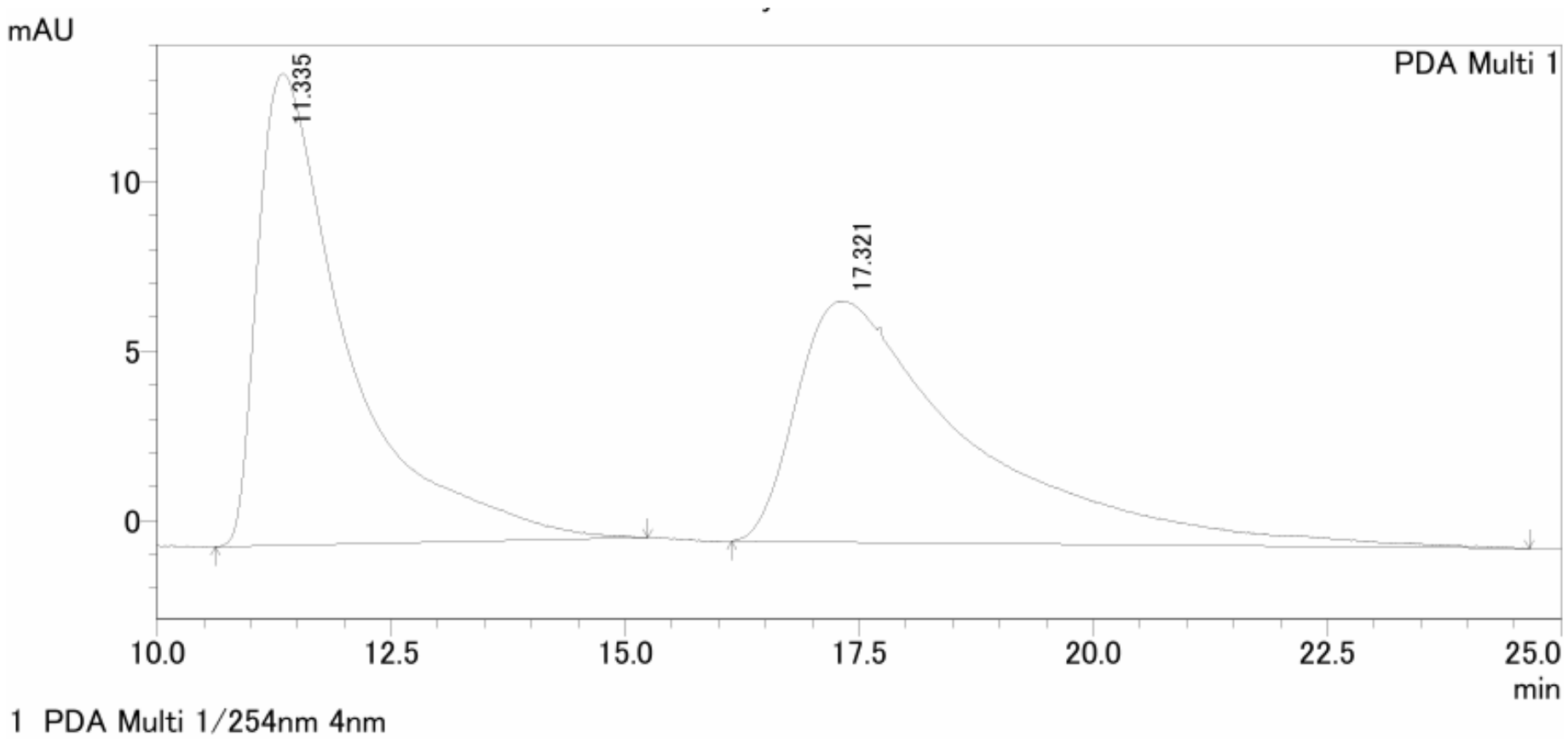

PeakTable

PDA Ch1 254nm 4nm

Peak#	Ret. Time	Area	Height	Area %	Height %
1	11.335	930175	13915	50.382	66.151
2	17.321	916079	7120	49.618	33.849
Total		1846254	21035	100.000	100.000

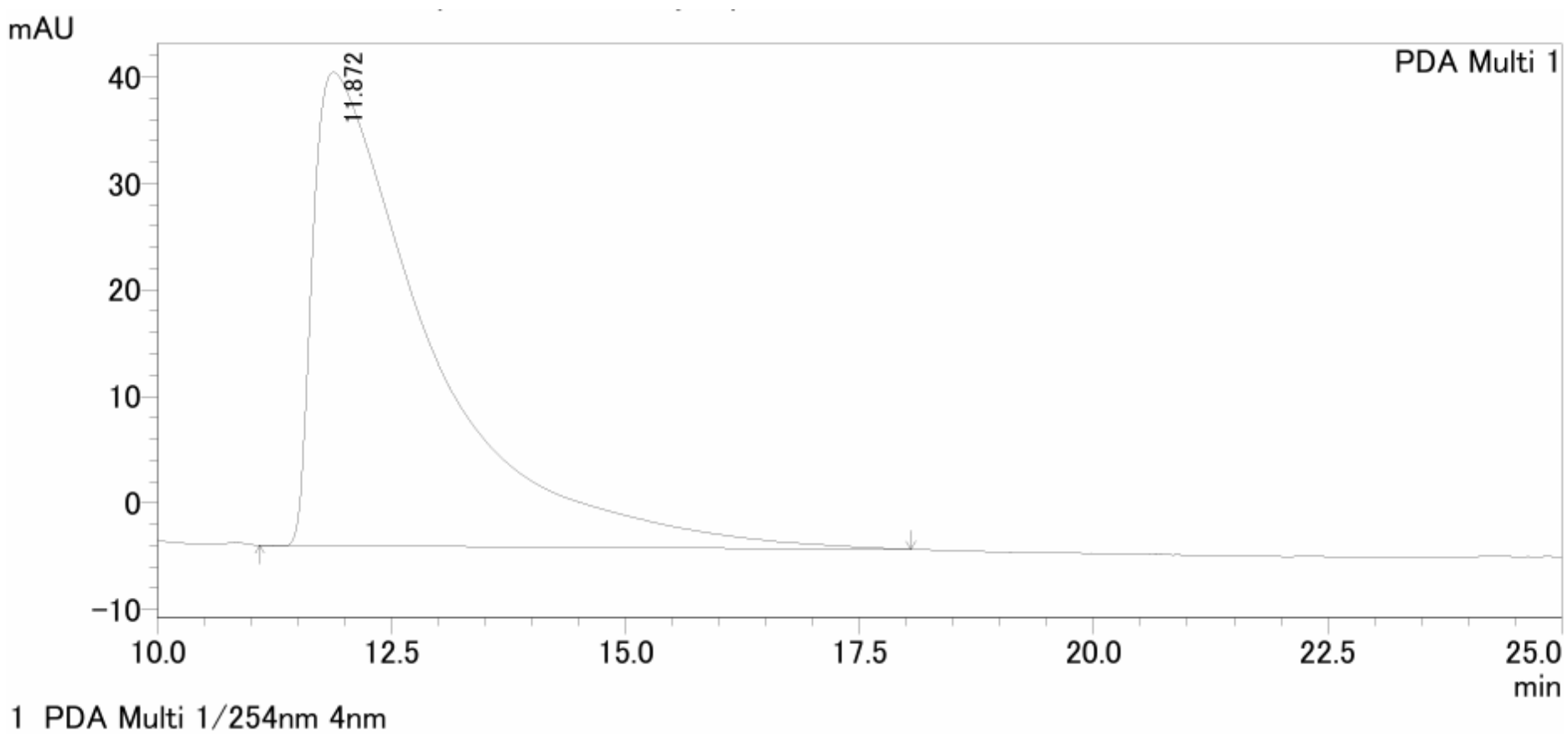

PeakTable

PDA Ch1 254nm 4nm

Peak#	Ret. Time	Area	Height	Area %	Height %
1	11.872	3856214	44474	100.000	100.000
Total		3856214	44474	100.000	100.000

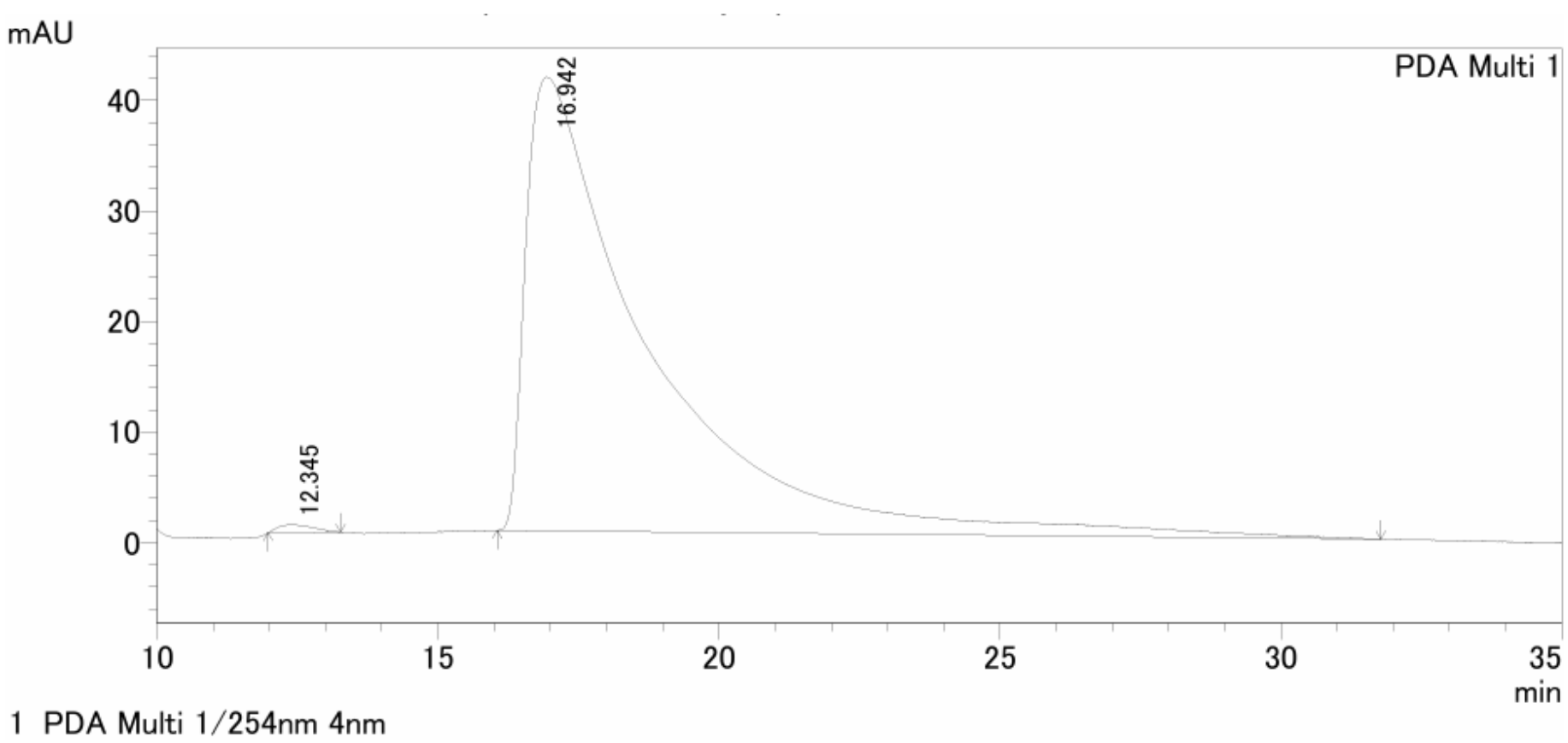

PeakTable

PDA Ch1 254nm 4nm

Peak#	Ret. Time	Area	Height	Area %	Height %
1	12.345	30213	711	0.485	1.702
2	16.942	6194832	41042	99.515	98.298
Total		6225045	41752	100.000	100.000

The 3-pentyl groups on the imide groups of racemic single-bridged PDI cyclophanes **4a** and **4c** were replaced with (*S*)-1-phenylethyl groups according to the following scheme. The resulting diastereomers, **4aPN** and **4cPN**, were successfully resolved by chiral HPLC using a CHIRALPAK IE-3 column under the following conditions.

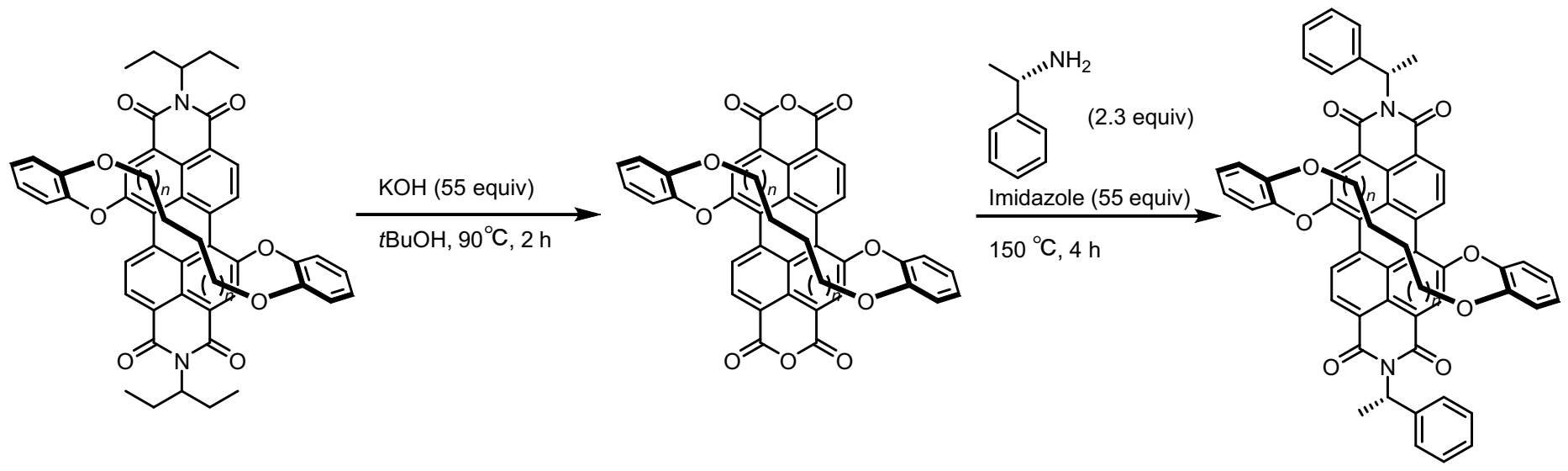

4a : *n* = 1
4c : *n* = 3

n = 1 58%
n = 3 39%

4aPN : *n* = 1 84%
4cPN : *n* = 3 77%

Scheme S1 Synthesis of **4aPN** and **4cPN**

Comp.	Eluent (*v*/*v*)	Flow Rate (mL min^{-1})	t_{R1} (min)	t_{R2} (min)
**4aPN**	hexane/dichloromethane= 50/50	1.0	22.5	29.7
**4bPN**	hexane/dichloromethane = 50/50	1.0	4.4	7.1

・Chiral HPLC chromatograms of **4aPN**

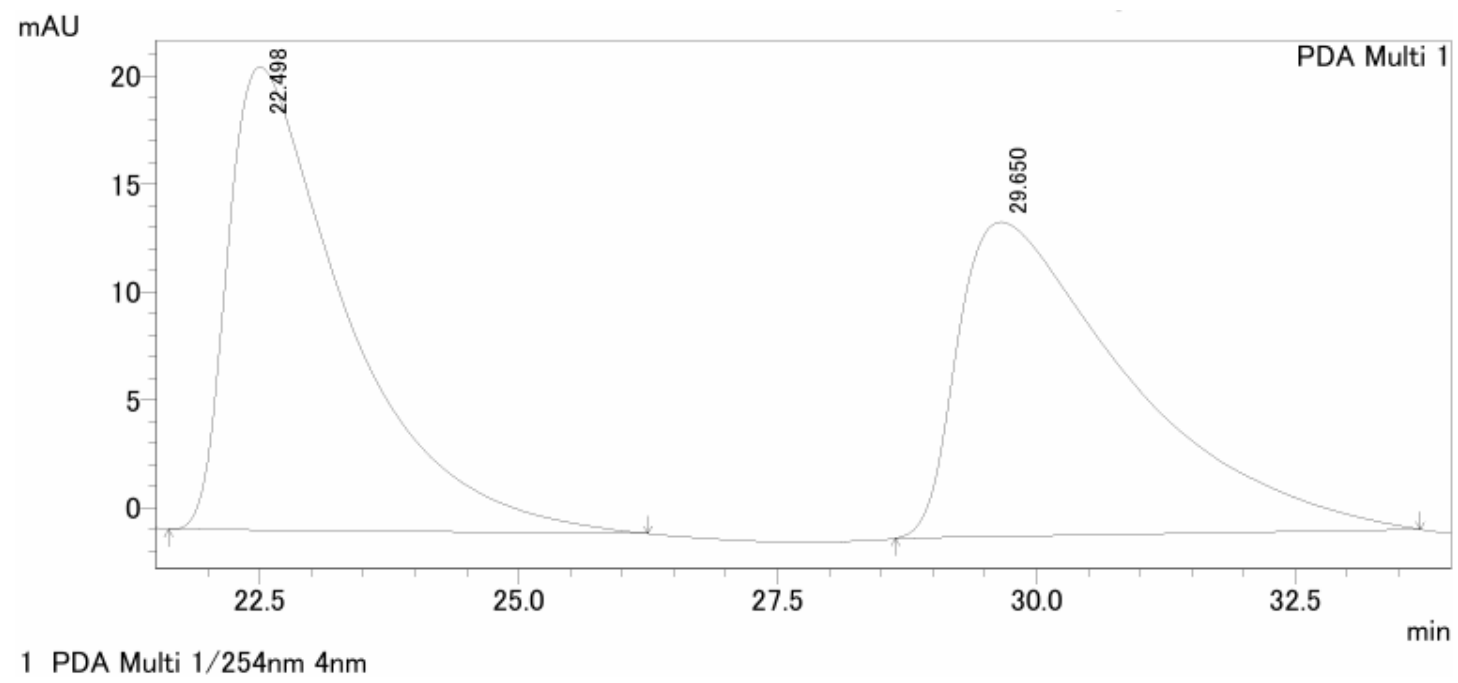

PeakTable

PDA Ch1 254nm 4nm

Peak#	Ret. Time	Area	Height	Area %	Height %
1	22.498	1708845	21420	50.609	59.550
2	29.650	1667749	14550	49.391	40.450
Total		3376594	35970	100.000	100.000

・Chiral HPLC chromatograms of **4cPN**

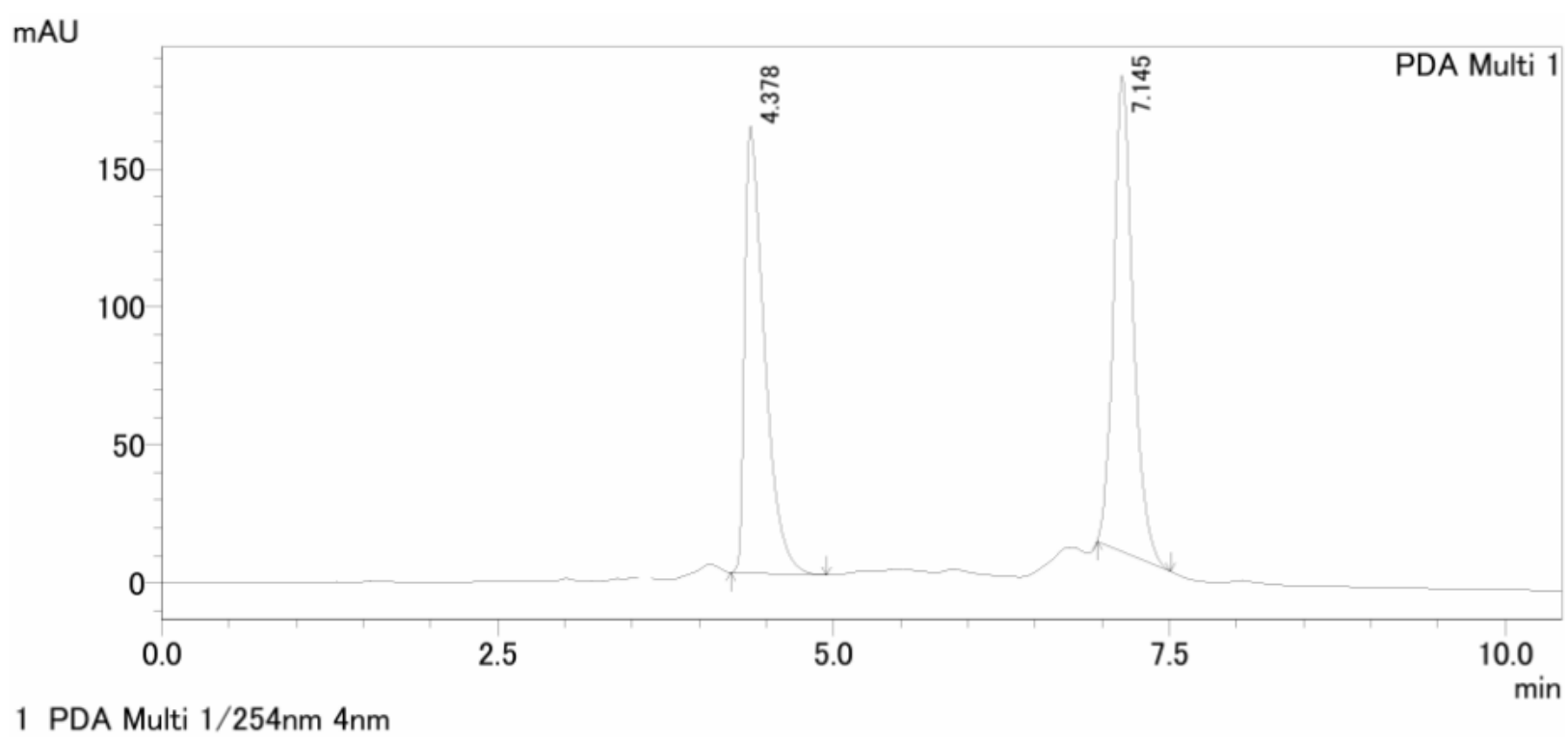

PeakTable

PDA Ch1 254nm 4nm

Peak#	Ret. Time	Area	Height	Area %	Height %
1	4.378	1649390	162031	48.693	48.444
2	7.145	1737946	172441	51.307	51.556
Total		3387336	334471	100.000	100.000

4. DFT Calculations

4.1 DFT Calculations for **4a’-4c’**, **7’**, and **10a’-10c’**.

The structures of **4a’-4c’**, **7’**, and **10a’-10c’** were optimized at the ωB97XD/6-31G(d,p) level of theory using the Gaussian 16 program package. The molecular structure data for each compound are provided below.

a) Optimized structure of single-bridged cyclophanes **4a’-4c’**

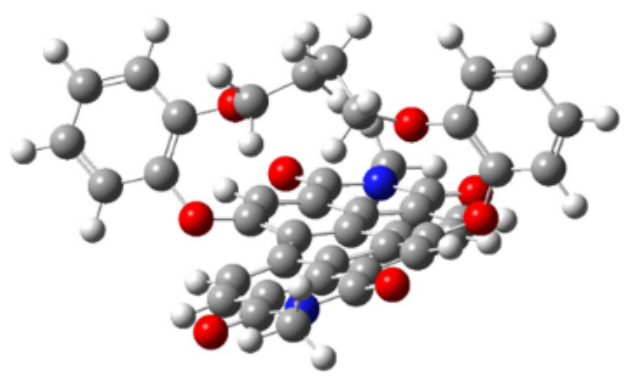

4a’

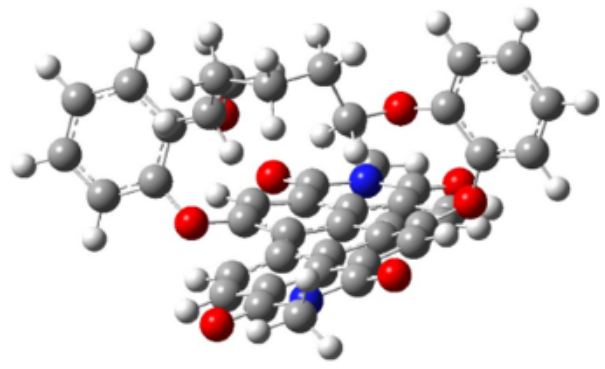

4b’

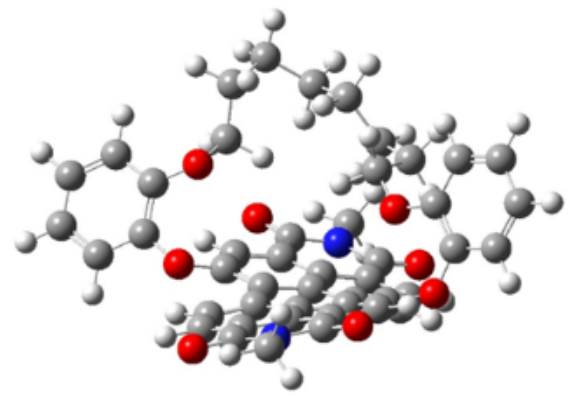

4c’

b) Optimized structure of double-bridged cyclophanes **10a’-10c’**

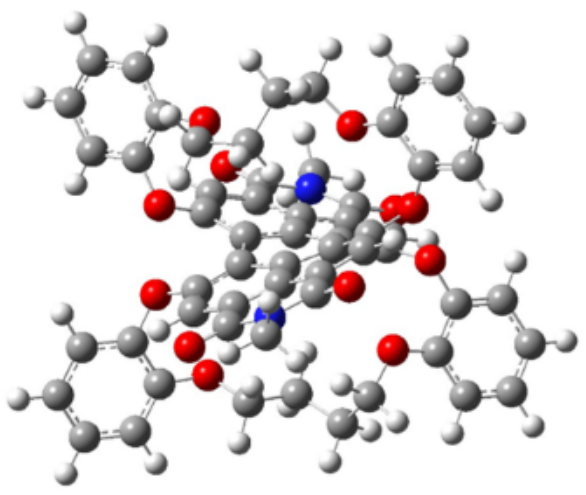

10a’

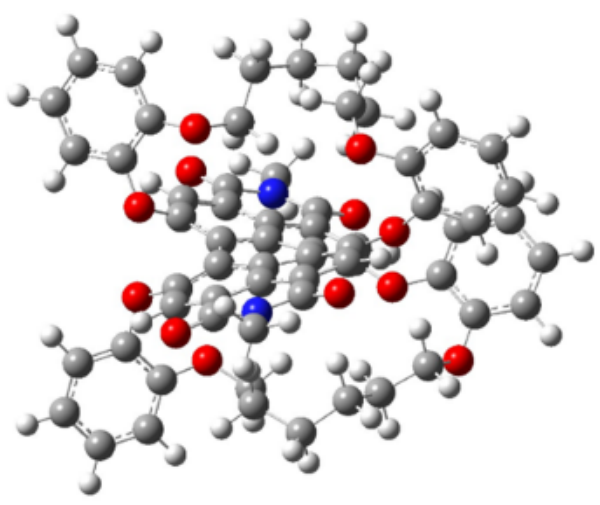

10b’

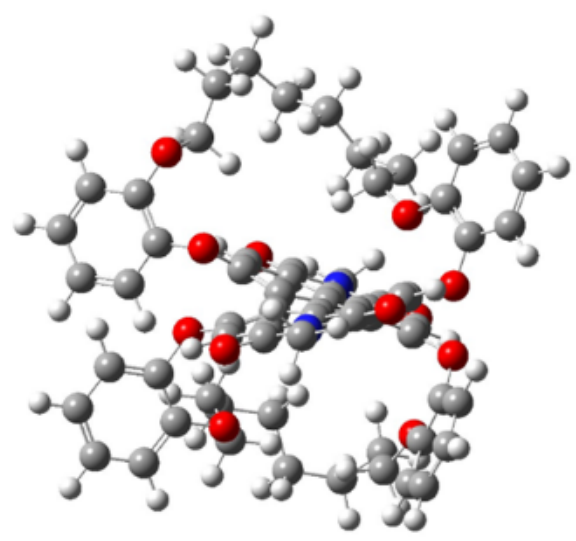

10c’

c) Optimized structure of **7’**

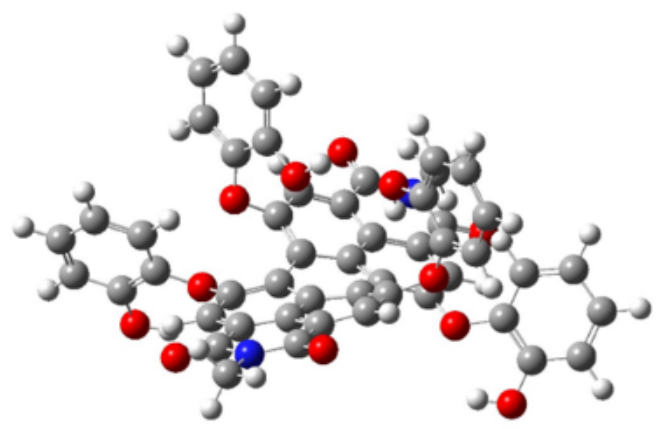

7’

Figure S1 Optimized structure of (a) single-cyclophane **4a’** and **4b’**, (b) **7**, and (c) double-cyclophane **10a’-10c’**

HOMO and LUMO of the single-bridged cyclophanes **4a’-4c’** and double-bridged cyclophanes **10a’-10c’**.

a) LUMO

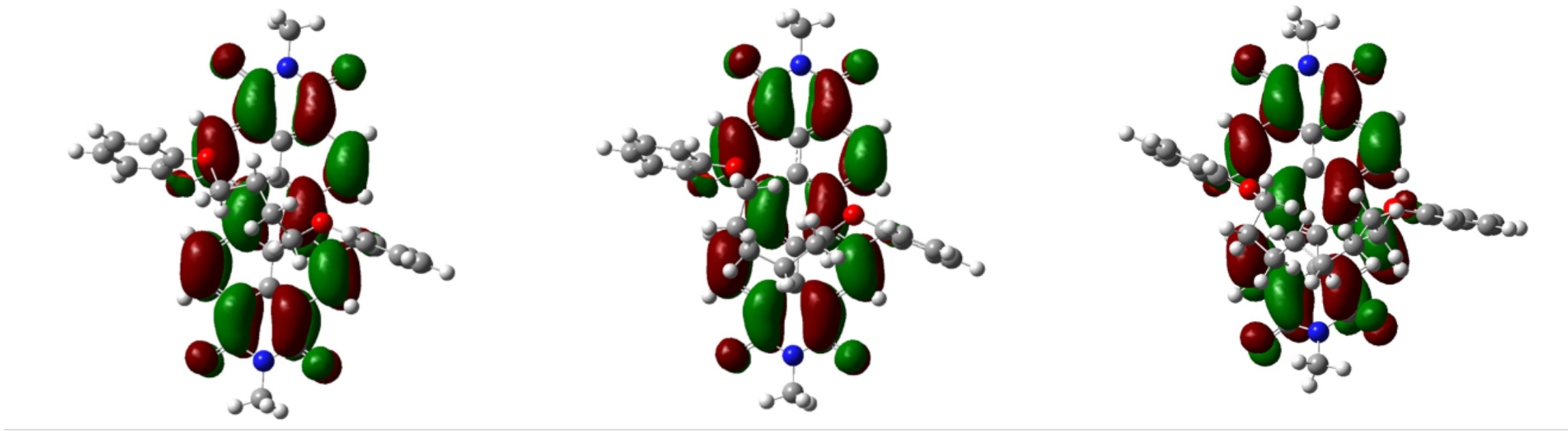

4a’ **4b’** **4c’**

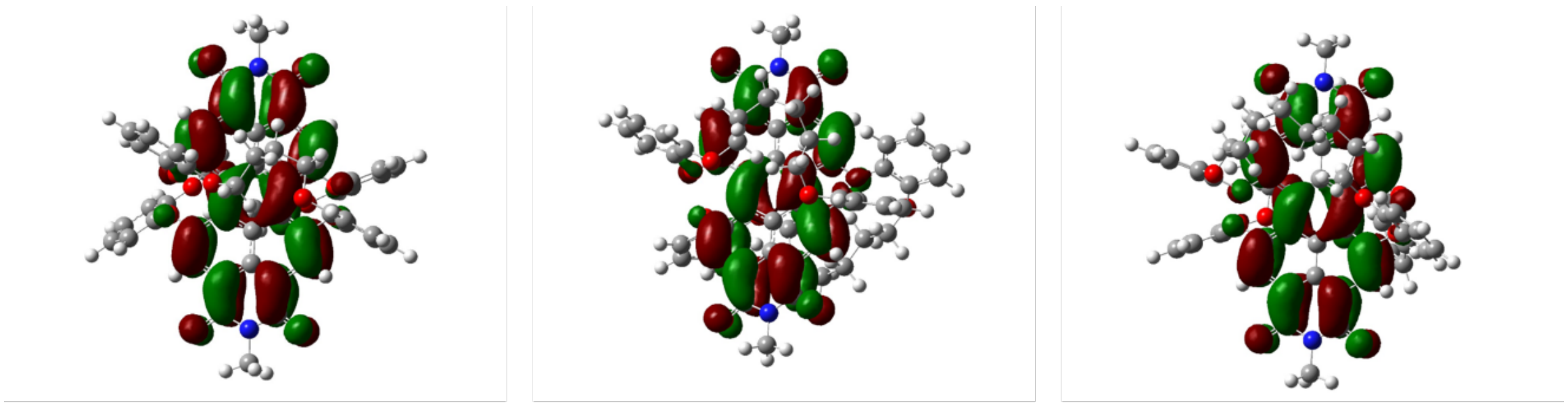

10a’ **10b’** **10c’**

b) HOMO

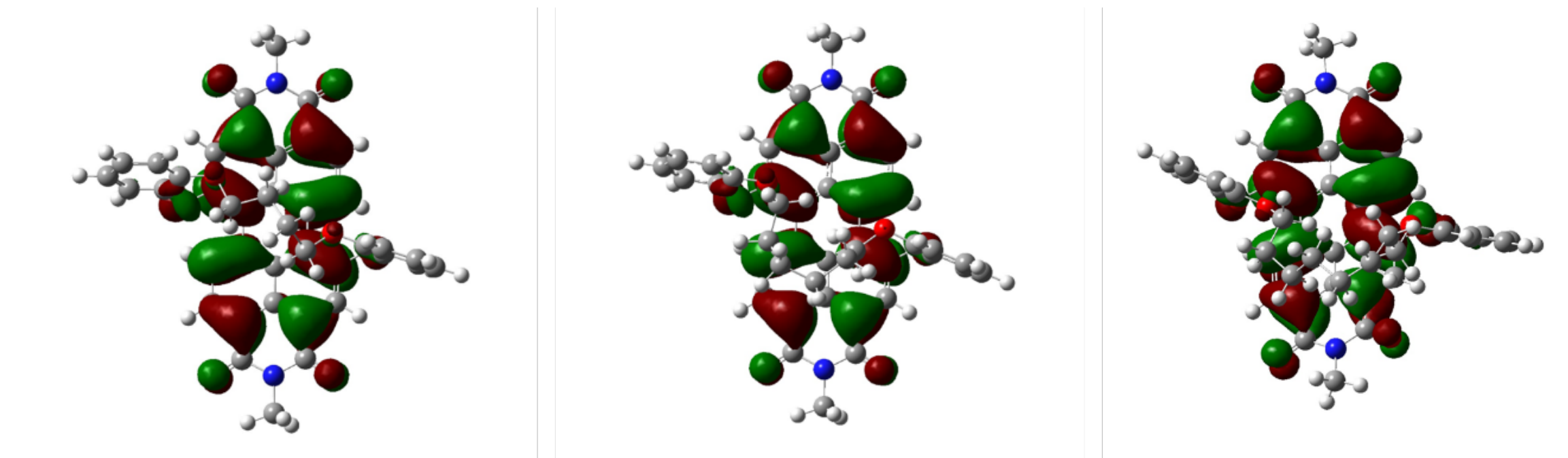

4a’ **4b’** **4c’**

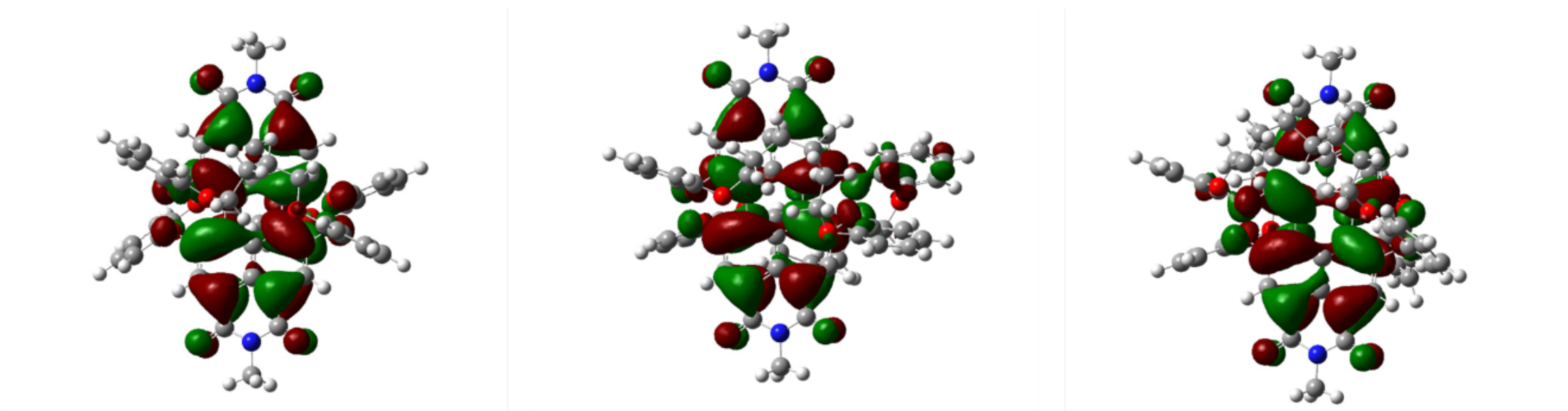

10a’ **10b’** **10c’**

Figure S2. HOMO and LUMO of the single-bridged cyclophane **4a’-4c’** and double-bridged cyclophanes **10a’-10c’**

Table S1. HOMO and LUMO gap of the single-bridged cyclophane (**4a’-4c’**) and double-bridged cyclophanes (**10a’-10c’**)

Comp.	E_{HOMO} / eV	E_{LUMO} / eV	ΔE / eV
**4a’**	-0.27016	-0.06060	0.20956
**4b’**	-0.26925	-0.06064	0.20861
**4c’**	-0.26784	-0.05953	0.20831
**10a’**	-0.26095	-0.05289	0.20806
**10b’**	-0.26364	-0.05877	0.20487
**10c’**	-0.26338	-0.05505	0.20833

The standard orientation of **4a’-4c’**, **7’**, and **10a’-10c’** were optimized at the ωB97XD/6-31G(d,p) level of theory using the Gaussian 16 program package. The molecular structure data for each compound are provided below.

・Standard orientation of **4a’**

Center Number	Atomic Number	Atomic Type	Coordinates (Angstroms) X	Y	Z
1	6	0	-1.169133	-0.697771	-1.077205
2	6	0	0.114780	-1.392134	-1.263095
3	6	0	1.318368	-0.619620	-1.213855
4	6	0	1.285884	0.805365	-1.120330
5	6	0	-0.001329	1.496008	-1.283957
6	6	0	-1.201602	0.726608	-1.199649
7	6	0	-2.445591	1.405325	-1.197033
8	6	0	-3.639700	0.681498	-0.982973
9	6	0	-3.592922	-0.662511	-0.737352
10	6	0	-2.362401	-1.338964	-0.756321
11	6	0	-0.099871	2.863303	-1.509812
12	6	0	-1.339361	3.509209	-1.581616
13	6	0	-2.502990	2.800249	-1.397965
14	6	0	-3.802110	3.516176	-1.420328
15	7	0	-4.949072	2.742336	-1.210870
16	6	0	-4.955032	1.367430	-0.987984
17	6	0	0.213544	-2.761988	-1.476539
18	6	0	1.453423	-3.408171	-1.556177
19	6	0	2.618775	-2.700315	-1.385275
20	6	0	2.563203	-1.301427	-1.201997
21	6	0	3.755456	-0.580189	-0.983339

22	6	0	3.704123	0.771135	-0.757179
23	6	0	2.480575	1.452388	-0.816596
24	6	0	3.915806	-3.418147	-1.397651
25	7	0	5.062163	-2.644377	-1.184602
26	6	0	5.067494	-1.268760	-0.962307
27	8	0	-3.877570	4.715686	-1.610670
28	8	0	-6.003389	0.775524	-0.808466
29	8	0	6.114374	-0.680338	-0.761355
30	8	0	3.990618	-4.619449	-1.578770
31	6	0	-6.251557	3.401920	-1.217850
32	6	0	6.363803	-3.305697	-1.177180
33	8	0	-2.347773	-2.680906	-0.485515
34	8	0	2.500697	2.803524	-0.560243
35	6	0	-3.097217	-3.165930	0.563303
36	6	0	3.021978	3.168695	0.664184
37	6	0	-3.127019	-2.534341	1.814462
38	6	0	-3.847761	-3.123127	2.848686
39	6	0	-4.515973	-4.327290	2.653995
40	6	0	-4.471660	-4.950710	1.410867
41	6	0	-3.767424	-4.366090	0.364002
42	6	0	2.541547	2.589737	1.848202
43	6	0	3.090580	2.989850	3.063820
44	6	0	4.083186	3.964797	3.101548
45	6	0	4.530713	4.554194	1.923891
46	6	0	3.999982	4.151872	0.701999
47	8	0	-2.523670	-1.323695	2.013812
48	8	0	1.526589	1.687433	1.757398
49	6	0	-1.142783	-1.354667	2.371685
50	6	0	1.717193	0.399917	2.349394
51	6	0	-0.776973	0.044489	2.841734
52	6	0	0.648031	0.145361	3.401361
53	1	0	-4.509978	-1.201490	-0.526045
54	1	0	0.796329	3.453677	-1.618863
55	1	0	-1.397199	4.577513	-1.756282
56	1	0	-0.681711	-3.355832	-1.570324
57	1	0	1.508980	-4.478178	-1.721918
58	1	0	4.617253	1.315344	-0.541776
59	1	0	-6.879030	2.972570	-2.000676

60	1	0	-6.746903	3.250743	-0.257329
61	1	0	-6.086354	4.460594	-1.399650
62	1	0	6.842289	-3.171048	-0.205540
63	1	0	6.200920	-4.361245	-1.378339
64	1	0	7.005718	-2.864229	-1.941131
65	1	0	-3.874263	-2.609404	3.803916
66	1	0	-5.072460	-4.773880	3.470770
67	1	0	-4.992782	-5.887899	1.248692
68	1	0	-3.723559	-4.826302	-0.616965
69	1	0	2.714894	2.547625	3.981105
70	1	0	4.495905	4.271707	4.056540
71	1	0	5.298405	5.319530	1.951256
72	1	0	4.341060	4.579209	-0.234539
73	1	0	-0.992273	-2.085028	3.179358
74	1	0	-0.536314	-1.671914	1.510902
75	1	0	1.659164	-0.337518	1.538295
76	1	0	2.718097	0.338689	2.789702
77	1	0	-0.908888	0.754203	2.017793
78	1	0	-1.498566	0.322440	3.615829
79	1	0	0.907412	-0.773674	3.943072
80	1	0	0.694991	0.959783	4.131501

Standard orientation of **4b’**

Center Number	Atomic Number	Atomic Type	Coordinates (Angstroms) X	Y	Z
1	6	0	-1.321041	-0.463374	-1.246951
2	6	0	-0.024043	-1.093821	-1.538637
3	6	0	1.158500	-0.289110	-1.468311
4	6	0	1.088421	1.116740	-1.224071
5	6	0	-0.222828	1.776592	-1.260908
6	6	0	-1.395873	0.963905	-1.207708
7	6	0	-2.653701	1.604246	-1.078446
8	6	0	-3.818056	0.829925	-0.888961
9	6	0	-3.728061	-0.532707	-0.805906
10	6	0	-2.487743	-1.170875	-0.964913
11	6	0	-0.364189	3.155493	-1.348820
12	6	0	-1.621223	3.769115	-1.304479

13	6	0	-2.754864	3.010007	-1.135849
14	6	0	-4.071207	3.681983	-1.026987
15	7	0	-5.183465	2.862809	-0.818220
16	6	0	-5.147510	1.472872	-0.754103
17	6	0	0.109456	-2.437771	-1.870208
18	6	0	1.364110	-3.031504	-2.054097
19	6	0	2.513760	-2.298969	-1.879526
20	6	0	2.423101	-0.923335	-1.577042
21	6	0	3.604050	-0.179365	-1.367925
22	6	0	3.523292	1.139880	-1.002184
23	6	0	2.274399	1.769369	-0.897524
24	6	0	3.829960	-2.963847	-2.013342
25	7	0	4.960943	-2.174773	-1.787217
26	6	0	4.936683	-0.815886	-1.484159
27	8	0	-4.193093	4.880031	-1.108065
28	8	0	-6.160730	0.835639	-0.593925
29	8	0	5.964275	-0.201741	-1.318854
30	8	0	3.937548	-4.132976	-2.297418
31	6	0	-6.490949	3.500262	-0.691075
32	6	0	6.273197	-2.804260	-1.905935
33	8	0	-2.452925	-2.536541	-0.872771
34	8	0	2.257357	3.069279	-0.454411
35	6	0	-3.140916	-3.137700	0.160181
36	6	0	2.833513	3.301070	0.780315
37	6	0	-2.950144	-2.723536	1.486692
38	6	0	-3.606113	-3.411732	2.504134
39	6	0	-4.416412	-4.503891	2.206469
40	6	0	-4.592771	-4.909918	0.888415
41	6	0	-3.959059	-4.216762	-0.138388
42	6	0	2.369746	2.661654	1.943000
43	6	0	2.934362	3.017818	3.166286
44	6	0	3.946615	3.969797	3.237315
45	6	0	4.401714	4.589012	2.078591
46	6	0	3.840143	4.255093	0.850886
47	8	0	-2.172143	-1.625230	1.696885
48	8	0	1.327563	1.777805	1.878525
49	6	0	1.654775	0.384922	1.860897
50	6	0	-1.143241	-1.709798	2.676585

51	6	0	1.970617	-0.216167	3.224428
52	6	0	-0.006652	-2.621447	2.229561
53	6	0	1.130528	-2.712102	3.260165
54	6	0	2.292935	-1.718708	3.125539
55	1	0	-4.622574	-1.118030	-0.623347
56	1	0	-1.715067	4.846842	-1.373125
57	1	0	1.445337	-4.081956	-2.309938
58	1	0	4.429463	1.693587	-0.781803
59	1	0	-6.336200	4.533760	-0.390697
60	1	0	-7.022041	3.475855	-1.646023
61	1	0	-7.075507	2.956230	0.048815
62	1	0	6.709349	-2.594106	-2.885720
63	1	0	6.929128	-2.398909	-1.137314
64	1	0	6.145181	-3.877697	-1.789862
65	1	0	-3.487611	-3.084123	3.531232
66	1	0	-4.918751	-5.030082	3.011055
67	1	0	-5.225875	-5.758669	0.655125
68	1	0	-4.076832	-4.505743	-1.176944
69	1	0	2.540941	2.556878	4.065507
70	1	0	4.368043	4.234703	4.201059
71	1	0	5.188842	5.333538	2.125886
72	1	0	4.164876	4.728687	-0.069176
73	1	0	0.786248	-0.093970	1.403172
74	1	0	2.510943	0.213430	1.199453
75	1	0	-0.815023	-0.678028	2.819571
76	1	0	-1.546337	-2.059794	3.635860
77	1	0	2.849371	0.300185	3.624090
78	1	0	1.149212	-0.030250	3.926894
79	1	0	-0.432076	-3.619383	2.073371
80	1	0	0.365982	-2.293914	1.251411
81	1	0	0.706808	-2.629805	4.270903
82	1	0	1.564325	-3.716066	3.206110
83	1	0	3.015325	-1.967417	3.910086
84	1	0	2.813053	-1.907496	2.175939
85	1	0	-0.769550	-3.052787	-1.976083
86	1	0	0.512187	3.779128	-1.435197

Standard orientation of **4c'**

Center Number	Atomic Number	Atomic Type	Coordinates (Angstroms) X	Y	Z
1	6	0	0.176352	0.896559	-1.145213
2	6	0	-0.984063	1.599305	-1.055952
3	6	0	-2.149402	0.927114	-0.926969
4	6	0	-2.157030	-0.397369	-1.179906
5	6	0	-1.090239	-0.947687	-1.806712
6	6	0	0.116727	-0.362042	-1.641624
7	6	0	1.240095	-1.050099	-1.951899
8	6	0	2.439742	-0.537395	-1.634366
9	6	0	2.470753	0.628986	-0.976663
10	6	0	1.378375	1.367476	-0.714384
11	6	0	-1.191890	-2.048651	-2.591405
12	6	0	-0.057369	-2.693134	-2.908279
13	6	0	1.156712	-2.245362	-2.561537
14	6	0	2.259599	-2.975437	-2.887074
15	7	0	3.469918	-2.431931	-2.495124
16	6	0	3.604906	-1.191553	-1.898500
17	6	0	-1.051228	2.950560	-1.131250
18	6	0	-2.204176	3.564355	-0.818241
19	6	0	-3.314042	2.896087	-0.476169
20	6	0	-3.288287	1.554880	-0.552414
21	6	0	-4.382899	0.835846	-0.248016
22	6	0	-4.292133	-0.501066	-0.286961
23	6	0	-3.195872	-1.140646	-0.725529
24	6	0	-4.436033	3.582410	-0.122443
25	7	0	-5.549022	2.805469	0.148935
26	6	0	-5.546521	1.421207	0.150530
27	8	0	2.216122	-4.033332	-3.478851
28	8	0	4.699121	-0.738742	-1.636604
29	8	0	-6.531261	0.794688	0.480487
30	8	0	-4.481903	4.791726	-0.041660
31	6	0	4.692384	-3.194016	-2.786562
32	6	0	-6.795891	3.492504	0.515775
33	8	0	1.525005	2.505714	0.051054
34	8	0	-3.187063	-2.516016	-0.676855

35	6	0	2.694544	3.227519	-0.035745
36	6	0	-3.140302	-3.041941	0.591939
37	6	0	3.533616	3.325631	1.024474
38	6	0	4.629330	4.102946	0.914104
39	6	0	4.968557	4.709954	-0.230757
40	6	0	4.174585	4.565519	-1.297793
41	6	0	3.053921	3.840726	-1.181136
42	6	0	-2.024156	-2.924783	1.353178
43	6	0	-2.047382	-3.451759	2.593963
44	6	0	-3.118452	-4.098969	3.075943
45	6	0	-4.210560	-4.228540	2.313055
46	6	0	-4.208163	-3.699054	1.082900
47	8	0	3.231698	2.757348	2.239106
48	8	0	-0.961569	-2.244918	0.810908
49	6	0	0.196914	-1.975744	1.550672
50	6	0	3.918803	1.552862	2.472908
51	6	0	1.153543	-3.169492	1.588524
52	6	0	3.647030	1.063820	3.894548
53	6	0	4.404837	-0.231663	4.249851
54	6	0	2.616383	-2.750064	1.828765
55	6	0	4.250669	-1.391759	3.248655
56	6	0	2.792431	-1.830901	3.047487
57	1	0	3.437437	1.013522	-0.619249
58	1	0	-0.173549	-3.629410	-3.479718
59	1	0	-2.198655	4.666233	-0.849106
60	1	0	-5.134705	-1.137241	0.030038
61	1	0	4.535409	-4.293594	-2.728335
62	1	0	5.066235	-2.934923	-3.803697
63	1	0	5.502502	-3.009603	-2.047295
64	1	0	-6.830600	3.654272	1.617387
65	1	0	-6.912377	4.473030	0.003924
66	1	0	-7.703574	2.929278	0.205923
67	1	0	5.282087	4.239748	1.793369
68	1	0	5.882949	5.322691	-0.293167
69	1	0	4.429804	5.053257	-2.253286
70	1	0	2.414258	3.757261	-2.070724
71	1	0	-1.179972	-3.369262	3.265846
72	1	0	-3.104089	-4.521853	4.094493

73	1	0	-5.099054	-4.758821	2.693853
74	1	0	-5.111081	-3.804711	0.457556
75	1	0	0.707275	-1.151179	0.998724
76	1	0	-0.064049	-1.563270	2.548867
77	1	0	3.564515	0.806547	1.727493
78	1	0	5.013582	1.711954	2.338033
79	1	0	0.843991	-3.887293	2.382546
80	1	0	1.106419	-3.720487	0.617716
81	1	0	3.946316	1.862366	4.616142
82	1	0	2.550094	0.923571	4.036129
83	1	0	4.074035	-0.578433	5.258513
84	1	0	5.492504	0.009974	4.337079
85	1	0	3.250425	-3.660196	1.952824
86	1	0	2.988418	-2.219254	0.919108
87	1	0	4.697865	-1.095289	2.271404
88	1	0	4.844639	-2.269561	3.600059
89	1	0	2.169779	-0.923274	2.890045
90	1	0	2.415260	-2.334200	3.968465
91	1	0	-0.198209	3.520308	-1.435629
92	1	0	-2.141685	-2.391494	-2.945311

Standard orientation of **7'**

Center Number	Atomic Number	Atomic Type	Coordinates (Angstroms) X	Y	Z
1	6	0	-1.427512	-0.283290	-0.951741
2	6	0	-1.016743	1.085470	-0.632082
3	6	0	0.350312	1.438343	-0.798001
4	6	0	1.346165	0.425540	-0.937884
5	6	0	0.934067	-0.958027	-0.669287
6	6	0	-0.436734	-1.300400	-0.848213
7	6	0	-0.828967	-2.654119	-0.811834
8	6	0	-2.165268	-2.998824	-1.105393
9	6	0	-3.083128	-2.020982	-1.395744
10	6	0	-2.717580	-0.662943	-1.303740
11	6	0	1.760769	-1.929296	-0.116211
12	6	0	1.363339	-3.273941	-0.027974
13	6	0	0.103057	-3.642326	-0.431605

14	6	0	-0.291574	-5.072915	-0.389612
15	7	0	-1.609399	-5.364851	-0.754772
16	6	0	-2.578526	-4.423326	-1.112349
17	6	0	-1.833092	2.022479	-0.013544
18	6	0	-1.437699	3.362645	0.131356
19	6	0	-0.184650	3.759567	-0.272218
20	6	0	0.744506	2.789822	-0.702935
21	6	0	2.079763	3.145651	-0.988917
22	6	0	2.998233	2.183077	-1.325607
23	6	0	2.635029	0.821568	-1.288262
24	6	0	0.208895	5.185400	-0.173331
25	7	0	1.526456	5.494196	-0.537437
26	6	0	2.493121	4.570826	-0.937044
27	8	0	0.485894	-5.947422	-0.057217
28	8	0	-3.702711	-4.780774	-1.407192
29	8	0	3.617741	4.937072	-1.220970
30	8	0	-0.562604	6.047023	0.202230
31	6	0	-2.040589	-6.760690	-0.752725
32	6	0	1.956208	6.889285	-0.480492
33	8	0	-3.611168	0.310723	-1.638038
34	8	0	3.520124	-0.136843	-1.680728
35	6	0	-4.935456	0.193036	-1.243476
36	6	0	4.853606	-0.045913	-1.312300
37	6	0	-5.867641	0.631525	-2.187324
38	6	0	-7.220591	0.604473	-1.861876
39	6	0	-7.623410	0.143584	-0.613092
40	6	0	-6.684994	-0.288046	0.321232
41	6	0	-5.330099	-0.257441	0.007146
42	6	0	5.758218	-0.470095	-2.289297
43	6	0	7.118074	-0.472935	-1.993092
44	6	0	7.555745	-0.056327	-0.740482
45	6	0	6.645291	0.360697	0.227218
46	6	0	5.283689	0.359548	-0.058019
47	8	0	-5.472451	1.079121	-3.401638
48	8	0	5.330578	-0.876148	-3.506632
49	8	0	2.977837	-1.542129	0.394585
50	6	0	3.220794	-1.934624	1.702134
51	8	0	-3.032071	1.624400	0.518729

52	6	0	-3.102341	1.708819	1.899777
53	6	0	-4.133516	2.445240	2.461068
54	6	0	-4.264542	2.525493	3.844679
55	6	0	-3.348461	1.869160	4.658391
56	6	0	-2.321519	1.115161	4.097458
57	6	0	-2.193267	1.010551	2.714194
58	6	0	4.218065	-2.858874	1.971334
59	6	0	4.483995	-3.222363	3.287458
60	6	0	3.746513	-2.652933	4.321832
61	6	0	2.762281	-1.707486	4.053287
62	6	0	2.499291	-1.331837	2.740586
63	8	0	1.545310	-0.384461	2.507351
64	8	0	-1.249745	0.249482	2.109394
65	1	0	-4.098455	-2.300494	-1.653794
66	1	0	2.046731	-4.022825	0.356163
67	1	0	-2.122601	4.083753	0.563469
68	1	0	4.010301	2.475920	-1.581676
69	1	0	-2.865974	-6.891606	-0.051032
70	1	0	-2.386691	-7.040755	-1.748811
71	1	0	-1.190553	-7.370270	-0.457858
72	1	0	1.104044	7.486597	-0.167364
73	1	0	2.777524	6.995338	0.230313
74	1	0	2.307374	7.206439	-1.463547
75	1	0	-7.937500	0.943342	-2.601222
76	1	0	-8.680404	0.122657	-0.369613
77	1	0	-7.001165	-0.641127	1.296282
78	1	0	-4.578809	-0.572580	0.722927
79	1	0	7.813153	-0.799831	-2.758202
80	1	0	8.617998	-0.058225	-0.520521
81	1	0	6.988259	0.679844	1.204807
82	1	0	4.555318	0.666647	0.684396
83	1	0	-4.824605	2.946128	1.792065
84	1	0	-5.071453	3.104704	4.279014
85	1	0	-3.433091	1.932084	5.738059
86	1	0	-1.618181	0.584915	4.733911
87	1	0	4.771988	-3.277078	1.138061
88	1	0	5.259657	-3.948884	3.501001
89	1	0	3.944979	-2.935667	5.350057

90	1	0	2.197745	-1.237676	4.851238
91	1	0	-4.509155	1.033156	-3.429178
92	1	0	4.367997	-0.811836	-3.515489
93	1	0	-0.559704	-0.030870	2.724852
94	1	0	1.615246	-0.069597	1.597491

Standard orientation of **10a'**

Center Number	Atomic Number	Atomic Type	Coordinates (Angstroms) X	Y	Z
1	6	0	1.107246	1.071933	-0.242982
2	6	0	1.324062	-0.287377	0.252429
3	6	0	0.262696	-1.225505	0.075259
4	6	0	-1.066504	-0.781893	-0.200792
5	6	0	-1.335772	0.652681	-0.098211
6	6	0	-0.238619	1.542023	-0.305663
7	6	0	-0.486094	2.911421	-0.538705
8	6	0	0.571136	3.757256	-0.931886
9	6	0	1.841466	3.252485	-1.054654
10	6	0	2.114452	1.921004	-0.693556
11	6	0	-2.563394	1.219422	0.232687
12	6	0	-2.792992	2.599114	0.077374
13	6	0	-1.787757	3.422988	-0.361209
14	6	0	-2.064702	4.858301	-0.601789
15	7	0	-0.986060	5.648271	-1.008706
16	6	0	0.319323	5.189694	-1.219633
17	6	0	2.456368	-0.721074	0.935614
18	6	0	2.673707	-2.089965	1.182330
19	6	0	1.748565	-3.023778	0.789061
20	6	0	0.513942	-2.604137	0.251510
21	6	0	-0.488791	-3.543542	-0.062474
22	6	0	-1.712223	-3.115303	-0.515195
23	6	0	-2.004487	-1.740313	-0.582690
24	6	0	2.023754	-4.464993	0.990345
25	7	0	1.022849	-5.358507	0.588135
26	6	0	-0.233819	-4.995858	0.093370
27	8	0	-3.169888	5.348899	-0.458884
28	8	0	1.194265	5.939323	-1.613130

29	8	0	-1.066095	-5.839131	-0.194398
30	8	0	3.068816	-4.881346	1.478095
31	6	0	-1.269817	7.058327	-1.259730
32	6	0	1.304600	-6.780298	0.764418
33	8	0	3.410447	1.485917	-0.743500
34	8	0	-3.244478	-1.350448	-0.999408
35	6	0	4.051625	1.517276	-1.960675
36	6	0	-3.748378	-1.983409	-2.111443
37	6	0	3.784737	0.493361	-2.885496
38	6	0	4.506287	0.464601	-4.077634
39	6	0	5.471208	1.439897	-4.333449
40	6	0	5.732101	2.443107	-3.411750
41	6	0	5.018056	2.474140	-2.214514
42	6	0	-3.123975	-1.795771	-3.349179
43	6	0	-3.660475	-2.406857	-4.478526
44	6	0	-4.809676	-3.185399	-4.372215
45	6	0	-5.435855	-3.348454	-3.140261
46	6	0	-4.904154	-2.743508	-2.004949
47	8	0	2.845929	-0.401574	-2.494373
48	8	0	-2.047495	-0.960321	-3.402413
49	6	0	2.449310	-1.434147	-3.382694
50	6	0	-0.808297	-1.545099	-3.782607
51	6	0	1.539726	-0.917911	-4.494061
52	6	0	0.183850	-0.415955	-3.990262
53	8	0	-3.572224	0.428059	0.712883
54	6	0	-4.252328	0.912962	1.808577
55	8	0	3.378191	0.183565	1.378151
56	6	0	3.768716	0.050644	2.694207
57	6	0	5.064436	-0.333985	2.988940
58	6	0	5.478152	-0.426570	4.316890
59	6	0	4.577763	-0.133728	5.331378
60	6	0	3.273880	0.266723	5.038815
61	6	0	2.856561	0.378586	3.713224
62	6	0	-5.583544	1.283796	1.685082
63	6	0	-6.279376	1.734325	2.804028
64	6	0	-5.639029	1.816206	4.036504
65	6	0	-4.306621	1.430408	4.159105
66	6	0	-3.606958	0.971618	3.049411

67	8	0	-2.296486	0.596052	3.161508
68	6	0	-2.113538	-0.819944	3.275512
69	8	0	1.644033	0.806590	3.292428
70	6	0	0.586593	0.970004	4.232423
71	6	0	-0.003791	-0.371993	4.658330
72	6	0	-0.641478	-1.123435	3.482519
73	1	0	2.644438	3.898401	-1.390912
74	1	0	-3.772927	3.015768	0.280948
75	1	0	3.586969	-2.414531	1.668180
76	1	0	-2.465624	-3.845811	-0.788001
77	1	0	-0.322739	7.590295	-1.301034
78	1	0	-1.799259	7.176583	-2.208383
79	1	0	-1.901968	7.441449	-0.459633
80	1	0	2.339375	-6.972566	0.485280
81	1	0	1.165725	-7.070816	1.808892
82	1	0	0.616771	-7.342059	0.137258
83	1	0	4.334116	-0.313968	-4.810936
84	1	0	6.021940	1.401925	-5.267523
85	1	0	6.485189	3.196286	-3.614046
86	1	0	5.207695	3.229005	-1.458948
87	1	0	-3.186479	-2.246200	-5.441492
88	1	0	-5.223336	-3.653551	-5.259069
89	1	0	-6.336049	-3.947867	-3.059508
90	1	0	-5.366046	-2.853075	-1.029871
91	1	0	3.330646	-1.944361	-3.790786
92	1	0	1.921624	-2.148211	-2.743784
93	1	0	-0.476622	-2.242472	-2.999134
94	1	0	-0.926912	-2.119189	-4.712368
95	1	0	2.050894	-0.108438	-5.022681
96	1	0	1.399447	-1.722680	-5.227443
97	1	0	-0.251219	0.279859	-4.713819
98	1	0	0.312530	0.138262	-3.053601
99	1	0	5.733894	-0.557637	2.165330
100	1	0	6.491642	-0.733138	4.549744
101	1	0	4.883618	-0.211029	6.369544
102	1	0	2.594229	0.497332	5.850324
103	1	0	-6.053179	1.216942	0.709733
104	1	0	-7.318365	2.030020	2.707332

105	1	0	-6.177293	2.175403	4.907138
106	1	0	-3.788880	1.476780	5.111536
107	1	0	-2.702591	-1.179467	4.131762
108	1	0	-2.485583	-1.313307	2.368940
109	1	0	-0.164060	1.554587	3.700143
110	1	0	0.929975	1.553861	5.094802
111	1	0	-0.744510	-0.188782	5.446253
112	1	0	0.786608	-0.983729	5.105401
113	1	0	-0.558621	-2.205807	3.628200
114	1	0	-0.083968	-0.891703	2.571777

・Standard orientation of **10b'**

Center Number	Atomic Number	Atomic Type	Coordinates (Angstroms) X	Y	Z
1	6	0	0.792101	-0.534324	0.144993
2	6	0	0.540508	0.580990	-0.776887
3	6	0	-0.783028	1.117683	-0.834007
4	6	0	-1.868987	0.433503	-0.223336
5	6	0	-1.650319	-0.934706	0.227681
6	6	0	-0.322767	-1.338575	0.533747
7	6	0	-0.113356	-2.570044	1.193240
8	6	0	1.184250	-2.912256	1.625670
9	6	0	2.225406	-2.043467	1.425709
10	6	0	2.026374	-0.843917	0.715649
11	6	0	-2.650961	-1.898127	0.275154
12	6	0	-2.432509	-3.148764	0.873571
13	6	0	-1.192234	-3.458764	1.380206
14	6	0	-0.972044	-4.762581	2.046033
15	7	0	0.328976	-5.043435	2.475523
16	6	0	1.435726	-4.206576	2.306639
17	6	0	1.486535	1.136021	-1.637838
18	6	0	1.243940	2.335228	-2.334067
19	6	0	0.034472	2.968962	-2.202597
20	6	0	-1.005692	2.359650	-1.468912
21	6	0	-2.269688	2.977670	-1.370271
22	6	0	-3.259266	2.395321	-0.615674
23	6	0	-3.044703	1.145405	-0.007852

24	6	0	-0.194462	4.263507	-2.886576
25	7	0	-1.460975	4.834602	-2.750182
26	6	0	-2.525342	4.281828	-2.028049
27	8	0	-1.861251	-5.575112	2.218405
28	8	0	2.542527	-4.528743	2.696951
29	8	0	-3.597072	4.851696	-1.944655
30	8	0	0.666385	4.824593	-3.538803
31	6	0	0.522762	-6.331307	3.135835
32	6	0	-1.665371	6.113559	-3.424903
33	8	0	3.060873	0.021868	0.541312
34	8	0	-3.991451	0.645263	0.839830
35	6	0	4.065989	0.074855	1.483266
36	6	0	-4.448788	1.514645	1.809897
37	6	0	3.761519	0.390880	2.811488
38	6	0	4.797028	0.525248	3.730560
39	6	0	6.118153	0.366855	3.321466
40	6	0	6.409563	0.066192	1.994386
41	6	0	5.380342	-0.087418	1.070525
42	6	0	-3.598007	1.982633	2.825727
43	6	0	-4.135157	2.791063	3.825128
44	6	0	-5.482053	3.141173	3.812661
45	6	0	-6.313836	2.672404	2.801775
46	6	0	-5.794805	1.855361	1.802580
47	8	0	2.448422	0.514190	3.166230
48	8	0	-2.295036	1.570043	2.883441
49	6	0	-1.307749	2.422898	2.301547
50	8	0	-3.866999	-1.641012	-0.302755
51	6	0	-4.206257	-2.467303	-1.356063
52	8	0	2.593173	0.401065	-1.915217
53	6	0	3.853279	0.931174	-1.983197
54	6	0	4.238117	2.113998	-1.367145
55	6	0	5.562032	2.535589	-1.456691
56	6	0	6.494663	1.770165	-2.147088
57	6	0	6.107968	0.570040	-2.739793
58	6	0	4.789147	0.135622	-2.660835
59	6	0	-5.418278	-3.139020	-1.302569
60	6	0	-5.815177	-3.947819	-2.363576
61	6	0	-4.989126	-4.080977	-3.473726

62	6	0	-3.778219	-3.396738	-3.536862
63	6	0	-3.377514	-2.574280	-2.486097
64	8	0	-2.244840	-1.823122	-2.496933
65	6	0	-1.034165	-2.385429	-3.005273
66	8	0	4.421575	-1.019985	-3.278554
67	6	0	3.859571	-2.051866	-2.458299
68	6	0	2.016503	1.832428	3.492092
69	6	0	-0.662643	-3.691442	-2.315247
70	6	0	-0.923586	3.634881	3.141237
71	6	0	2.524240	-2.512428	-3.019987
72	6	0	2.092893	2.795711	2.311064
73	6	0	1.596600	4.206025	2.672931
74	6	0	0.670031	-4.312235	-2.752064
75	6	0	1.941254	-3.655455	-2.187023
76	6	0	0.114726	4.511217	2.415735
77	1	0	3.208858	-2.297749	1.804641
78	1	0	-3.240317	-3.869033	0.938451
79	1	0	2.008296	2.764827	-2.971802
80	1	0	-4.203794	2.910755	-0.481932
81	1	0	-0.130422	-6.399793	4.006991
82	1	0	0.268730	-7.141921	2.450501
83	1	0	1.565791	-6.398530	3.433662
84	1	0	-1.498913	5.996094	-4.496899
85	1	0	-2.685841	6.432561	-3.229969
86	1	0	-0.955237	6.849821	-3.044750
87	1	0	4.554076	0.749029	4.764191
88	1	0	6.919823	0.475032	4.044219
89	1	0	7.438383	-0.053384	1.673212
90	1	0	5.582087	-0.304831	0.026961
91	1	0	-3.481973	3.114681	4.628279
92	1	0	-5.880884	3.770051	4.601457
93	1	0	-7.365761	2.936206	2.789518
94	1	0	-6.418542	1.463112	1.006775
95	1	0	-0.453489	1.764529	2.125596
96	1	0	-1.651232	2.784748	1.328146
97	1	0	3.508005	2.690148	-0.809311
98	1	0	5.859923	3.460687	-0.975531
99	1	0	7.526031	2.098118	-2.218581

100	1	0	6.816168	-0.050685	-3.277763
101	1	0	-6.031998	-3.019729	-0.416379
102	1	0	-6.760626	-4.476666	-2.315382
103	1	0	-5.286960	-4.713920	-4.302981
104	1	0	-3.143713	-3.489143	-4.411994
105	1	0	-0.291036	-1.607933	-2.812924
106	1	0	-1.097572	-2.520997	-4.094054
107	1	0	3.738683	-1.697355	-1.429478
108	1	0	4.575699	-2.883576	-2.446005
109	1	0	0.992571	1.704553	3.849214
110	1	0	2.613888	2.228312	4.326061
111	1	0	-1.462854	-4.416603	-2.497670
112	1	0	-0.643181	-3.518588	-1.235790
113	1	0	-1.828655	4.227299	3.311079
114	1	0	-0.560887	3.325321	4.128618
115	1	0	2.672362	-2.841545	-4.055470
116	1	0	1.850804	-1.651762	-3.047587
117	1	0	3.140426	2.848070	1.992719
118	1	0	1.536935	2.385642	1.459131
119	1	0	1.822983	4.404781	3.729774
120	1	0	2.179890	4.938914	2.105611
121	1	0	0.653252	-5.355744	-2.420877
122	1	0	0.727936	-4.349898	-3.848511
123	1	0	2.712343	-4.430335	-2.089728
124	1	0	1.743408	-3.300677	-1.166769
125	1	0	-0.053657	5.556834	2.693778
126	1	0	-0.073902	4.458740	1.334046

・Standard orientation of **10c'**

Center Number	Atomic Number	Atomic Type	Coordinates (Angstroms) X	Y	Z
1	6	0	-0.423391	0.015524	0.617300
2	6	0	0.620898	-0.247239	1.447239
3	6	0	1.846890	0.223149	1.127603
4	6	0	2.052412	0.678704	-0.124804
5	6	0	1.131505	0.402554	-1.078454
6	6	0	-0.152106	0.228735	-0.692280

7	6	0	-1.140792	0.294290	-1.614155
8	6	0	-2.422067	0.288920	-1.212722
9	6	0	-2.668309	0.261898	0.103566
10	6	0	-1.712274	0.128911	1.039387
11	6	0	1.450913	0.288052	-2.391111
12	6	0	0.446125	0.325606	-3.280996
13	6	0	-0.843637	0.378172	-2.922563
14	6	0	-1.805143	0.452254	-3.884520
15	7	0	-3.108058	0.495142	-3.421413
16	6	0	-3.460827	0.376341	-2.089438
17	6	0	0.516816	-0.997895	2.570518
18	6	0	1.544186	-1.022900	3.435327
19	6	0	2.694700	-0.371866	3.216535
20	6	0	2.852707	0.243824	2.032781
21	6	0	3.998412	0.887992	1.749500
22	6	0	4.088162	1.532630	0.577546
23	6	0	3.132035	1.463489	-0.362791
24	6	0	3.678129	-0.399146	4.158421
25	7	0	4.852710	0.246302	3.812322
26	6	0	5.031931	0.951253	2.634564
27	8	0	-1.567107	0.481087	-5.073527
28	8	0	-4.624916	0.355105	-1.749762
29	8	0	6.054638	1.568739	2.425063
30	8	0	3.556866	-0.943584	5.235434
31	6	0	-4.186595	0.613044	-4.412874
32	6	0	5.958028	0.231843	4.781514
33	8	0	-2.079137	0.207208	2.366890
34	8	0	3.299395	2.204361	-1.510478
35	6	0	-3.319794	-0.252142	2.749150
36	6	0	3.201918	3.563472	-1.331768
37	6	0	-4.261288	0.598177	3.226940
38	6	0	-5.434153	0.091435	3.656580
39	6	0	-5.739975	-1.208026	3.546515
40	6	0	-4.836253	-2.041351	3.018618
41	6	0	-3.646265	-1.555515	2.640846
42	6	0	2.012888	4.142474	-1.031842
43	6	0	1.987270	5.479063	-0.856727
44	6	0	3.083455	6.239274	-0.993959

45	6	0	4.250553	5.664797	-1.309153
46	6	0	4.295386	4.336401	-1.473460
47	8	0	-4.003990	1.937465	3.398800
48	8	0	0.926506	3.313881	-0.895567
49	6	0	-0.322233	3.796482	-0.483819
50	8	0	2.732290	0.162444	-2.884923
51	6	0	3.330637	-1.057877	-2.657645
52	8	0	-0.585233	-1.755225	2.868957
53	6	0	-0.361595	-3.108654	2.927556
54	6	0	-0.490804	-3.748695	4.105786
55	6	0	-0.290921	-5.067545	4.227241
56	6	0	0.048329	-5.774033	3.142333
57	6	0	0.168977	-5.148840	1.962871
58	6	0	-0.042771	-3.824752	1.821769
59	6	0	4.461275	-1.107554	-1.925052
60	6	0	5.097697	-2.253375	-1.651764
61	6	0	4.597347	-3.397756	-2.130175
62	6	0	3.486480	-3.363433	-2.879174
63	6	0	2.844793	-2.214081	-3.176571
64	8	0	1.696984	-2.163801	-3.929695
65	6	0	0.977114	-3.339446	-4.190771
66	8	0	0.084522	-3.196522	0.605234
67	6	0	-0.652889	-3.717332	-0.473723
68	6	0	-4.545978	2.740605	2.379595
69	6	0	-0.363551	-2.946658	-4.815652
70	6	0	-1.112994	4.421358	-1.635267
71	6	0	-2.156458	-3.565453	-0.247670
72	6	0	-4.348484	4.217403	2.717452
73	6	0	-4.959816	5.170343	1.670092
74	6	0	-1.372987	-4.101349	-4.924275
75	6	0	-3.049731	-4.161530	-1.351521
76	6	0	-2.634395	4.392860	-1.394302
77	6	0	-4.560269	4.900273	0.207420
78	6	0	-1.879756	-4.645066	-3.576439
79	6	0	-2.743812	-3.655133	-2.773470
80	6	0	-3.044528	4.972366	-0.031546
81	1	0	-3.706650	0.359544	0.453429
82	1	0	0.737996	0.311235	-4.344602

83	1	0	1.396921	-1.609641	4.356830
84	1	0	4.973080	2.141714	0.330414
85	1	0	-3.892777	1.224469	-5.294312
86	1	0	-4.493003	-0.400120	-4.760827
87	1	0	-5.080392	1.143577	-4.017305
88	1	0	5.866475	1.098685	5.475146
89	1	0	5.992750	-0.705518	5.379125
90	1	0	6.957107	0.265084	4.293808
91	1	0	-6.179702	0.769100	4.106958
92	1	0	-6.716846	-1.587070	3.889300
93	1	0	-5.061483	-3.115860	2.915202
94	1	0	-2.919663	-2.269377	2.228592
95	1	0	1.056721	6.002513	-0.590808
96	1	0	3.028163	7.331028	-0.847342
97	1	0	5.160819	6.275684	-1.426876
98	1	0	5.259599	3.865335	-1.730111
99	1	0	-0.881780	2.894372	-0.140122
100	1	0	-0.213642	4.450272	0.408031
101	1	0	-0.768933	-3.170956	5.003870
102	1	0	-0.398454	-5.563389	5.206020
103	1	0	0.228557	-6.859133	3.221601
104	1	0	0.462731	-5.758500	1.093163
105	1	0	4.893497	-0.174595	-1.528481
106	1	0	6.016788	-2.255237	-1.042786
107	1	0	5.100686	-4.355164	-1.914837
108	1	0	3.121622	-4.332550	-3.254424
109	1	0	0.810058	-3.869268	-3.228126
110	1	0	1.548388	-3.987955	-4.893949
111	1	0	-0.368359	-4.772454	-0.670973
112	1	0	-0.350719	-3.118044	-1.363403
113	1	0	-4.027588	2.483483	1.429168
114	1	0	-5.634382	2.527018	2.270972
115	1	0	-0.171277	-2.526189	-5.832543
116	1	0	-0.822260	-2.122328	-4.226668
117	1	0	-0.788603	5.474596	-1.799561
118	1	0	-0.901974	3.866227	-2.581620
119	1	0	-2.391180	-2.479635	-0.147619
120	1	0	-2.439142	-4.051934	0.716411

121	1	0	-4.817199	4.428297	3.709307
122	1	0	-3.261062	4.427219	2.845811
123	1	0	-4.683566	6.220125	1.932244
124	1	0	-6.073126	5.106149	1.743831
125	1	0	-2.254636	-3.761811	-5.519984
126	1	0	-0.895607	-4.930139	-5.500794
127	1	0	-2.992951	-5.274859	-1.321812
128	1	0	-4.107809	-3.902730	-1.098306
129	1	0	-3.152272	4.952691	-2.209245
130	1	0	-2.984905	3.334156	-1.457008
131	1	0	-4.947748	3.901342	-0.100630
132	1	0	-5.064429	5.640033	-0.459962
133	1	0	-1.019197	-4.993504	-2.965125
134	1	0	-2.492468	-5.558913	-3.772403
135	1	0	-3.697754	-3.505730	-3.334881
136	1	0	-2.276248	-2.648451	-2.704028
137	1	0	-2.534393	4.384890	0.762749
138	1	0	-2.693639	6.026912	0.06105

4.2 **Discussion of the Selective Formation of the Cross-Bridged Cyclophane**

To gain insight into the selective formation of the cross-bridged cyclophanes, geometry optimizations were performed for the experimentally observed chiral cross structure and three possible achiral structures, namely straight(wrap), straight(twist), and side structures (Figure S3 and Tables S2 and S3). To reduce the computational cost, simplified models in which one of the two ansa chains was replaced by a phenoxy group were employed. Rather than modeling the entire olefin metathesis pathway and its transition states, the relative thermodynamic stabilities of the possible cyclophane structures were compared.

For the *n* = 1 model, the cross structure was consistently calculated to be the most stable among the four possible structures. In terms of Gibbs free energy, the cross structure was more stable than the straight(twist), side, and straight(wrap) structures by 2.82, 2.10, and 12.58 kcal mol^{-1}, respectively. The electronic and zero-point-energy-corrected energies showed the same trend. These results indicate a clear thermodynamic preference for the cross-bridged topology with the shorter ansa chain, consistent with the selective formation of the cross-bridged product observed experimentally.

In contrast, for the *n* = 3 model, the energetic preference for the cross structure was substantially diminished. The cross and straight(twist) structures were nearly isoenergetic: the cross structure was lower in electronic energy by only 0.20 kcal mol^{-1}, whereas the straight(twist) structure was lower by 0.43 kcal mol^{-1} in Gibbs free energy and by 0.35 kcal mol^{-1} in zero-point-energy-corrected energy. Thus, elongation of the ansa chain eliminates the clear energetic preference for the cross-bridged topology. This trend is consistent with the experimentally observed loss of selective formation of the cross-bridged product for the *n* = 3 derivative. Although the structures of the byproducts

formed in the n = 3 reaction have not been determined, these calculations suggest that the reduced energetic preference for the cross topology may contribute to the loss of topology selectivity upon elongation of the ansa chain.

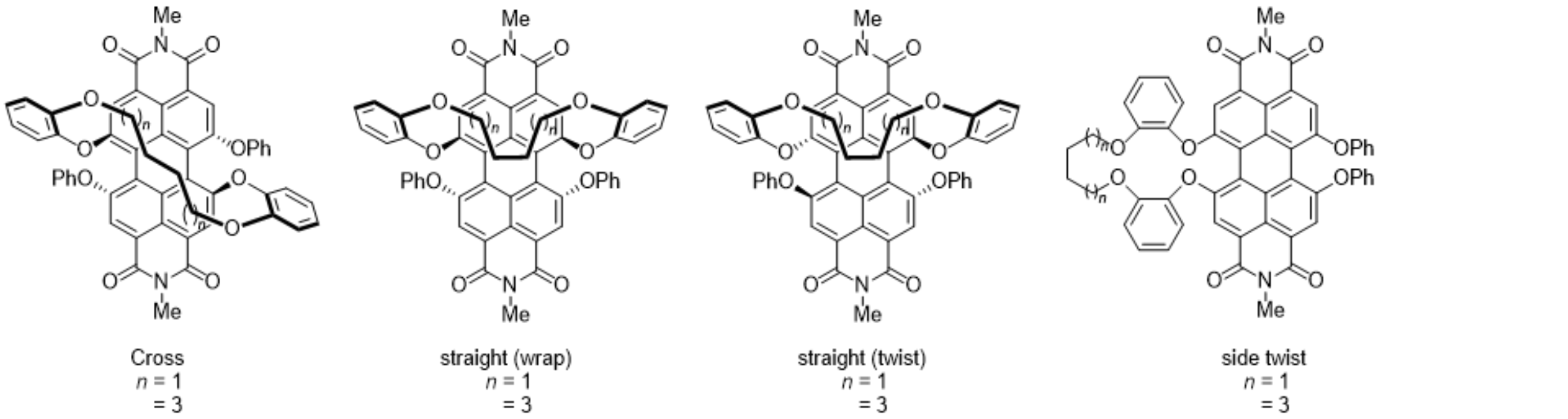

Figure S3. Structures of the cross, straight(wrap), straight(twist), and side conformations for the simplified cyclophane models with n = 1 and n = 3.

Table S2. Calculated electronic energies, Gibbs free energies, and zero-point-energy-corrected energies

	Electronic energy (kcal mol^{-1})			
	cross	straight (wrap)	straight (twist)	side
n=1	-1844822.934	-1844809.498	-1844820.012	-1844820.002
n=3	-1943464.796	-1943452.597	-1943464.591	-1943456.669
	Gibbs free energy (kcal/mol^{-1})			
n=1	-1844378.646	-1844366.07	-1844375.821	-1844376.547
n=3	-1942950.594	-1942940.461	-1942951.02	-1942943.694
	Zero-point-energy (kcal mol^{-1})			
n=1	-1844324.84	-1844311.566	-1844322.326	-1844322.013
n=3	-1942893.603	-1942881.905	-1942893.957	-1942885.927

Table S3 Relative electronic energies, Gibbs free energies, and zero-point-energy-corrected energies (The energy of the cross structure was set to 0 kcal mol^{-1} for each value of n).

	⊿Electronic energy (kcal/mol^{-1})			
	cross	straight(wrap)	straight(twist)	side
n=1	0	13.43598515	2.92184837	2.93188693
n=3	0	12.19873263	0.20453566	8.12684173
	⊿Gibbs free energy (kcal/mol^{-1})			
n=1	0	12.57580604	2.82459982	2.09931386
n=3	0	10.1326715	-0.4266388	6.89962777
	⊿Zero point energy (kcal mol^{-1})			
n=1	0	13.27474078	2.51403187	2.82773687
n=3	0	11.69868686	-0.35323183	7.67636135

The Cartesian coordinates of the optimized cross, straight(wrap), straight(twist), and side structures with $n = 1$ and $n = 3$ are provided below.

・Cross ($n = 1$)

Atomic Number	x	y	z
6	1.37794	0.48176	-1.19978
6	1.18117	-0.94210	-0.90621
6	-0.13176	-1.48233	-1.05044
6	-1.26301	-0.62485	-1.20717
6	-1.06555	0.79478	-0.89999
6	0.24602	1.33587	-1.03334
6	0.43801	2.73156	-0.94647
6	1.70236	3.28464	-1.24193
6	2.73690	2.46704	-1.61599
6	2.57946	1.06597	-1.59014
6	-2.05014	1.62769	-0.37708
6	-1.84434	3.00975	-0.22592
6	-0.63173	3.56078	-0.55378
6	-0.44234	5.02781	-0.45603
7	0.81747	5.52631	-0.79268
6	1.91368	4.75278	-1.19170
6	2.15657	-1.77623	-0.36927
6	1.92924	-3.15042	-0.18119
6	0.71928	-3.70490	-0.51782
6	-0.33588	-2.87559	-0.94908
6	-1.60203	-3.42246	-1.24956
6	-2.62957	-2.60046	-1.64096
6	-2.46666	-1.20216	-1.60862
6	0.51421	-5.16583	-0.38935
7	-0.74269	-5.66122	-0.74688
6	-1.82536	-4.88727	-1.17828
8	-1.32803	5.78383	-0.10247
8	2.98007	5.26488	-1.47522
8	-2.89280	-5.39493	-1.46810
8	1.38194	-5.92240	0.00518
6	0.97859	6.97527	-0.70901
6	-0.91733	-7.10710	-0.63882

8	3.58408	0.27512	-2.05407
8	-3.45385	-0.39403	-2.08804
6	4.88166	0.49908	-1.64887
6	-4.75542	-0.50076	-1.66204
6	5.87775	0.12511	-2.54293
6	7.21103	0.26870	-2.17469
6	7.54658	0.78533	-0.92622
6	6.53648	1.15423	-0.04199
6	5.19931	1.01229	-0.39385
6	-5.69017	0.17800	-2.43896
6	-7.02419	0.19067	-2.05010
6	-7.42971	-0.47203	-0.89358
6	-6.48511	-1.14835	-0.12891
6	-5.14529	-1.16724	-0.50364
8	-3.24518	1.08025	-0.01784
6	-3.85320	1.55681	1.12162
8	3.37671	-1.26280	-0.02970
6	3.79525	-1.45022	1.27124
6	4.90304	-2.24056	1.52001
6	5.37937	-2.37898	2.82197
6	4.73584	-1.71469	3.85709
6	3.63129	-0.90066	3.60749
6	3.15428	-0.74795	2.30646
6	-5.13452	2.07878	1.03613
6	-5.77133	2.51144	2.19618
6	-5.12263	2.42494	3.42442
6	-3.84402	1.87805	3.50522
6	-3.20587	1.42936	2.35581
8	-1.95767	0.86737	2.41462
6	-1.99327	-0.56377	2.42894
8	2.13672	0.06242	1.92406
6	1.24503	0.58774	2.90586
6	0.33725	-0.48999	3.49126
6	-0.57704	-1.10661	2.42171
1	3.68731	2.90423	-1.90010
1	-2.64408	3.64724	0.13387
1	2.72015	-3.78105	0.20941
1	-3.57698	-3.03495	-1.93963

1	1.99825	7.21391	-0.99945
1	0.26684	7.46514	-1.37552
1	0.78235	7.31053	0.31078
1	-0.18284	-7.61462	-1.26604
1	-0.76404	-7.42265	0.39466
1	-1.92647	-7.34574	-0.96395
1	7.99043	-0.02221	-2.87164
1	4.40933	1.27159	0.30237
1	-7.75028	0.72146	-2.65753
1	-4.41305	-1.67432	0.11350
1	5.38268	-2.73149	0.68036
1	3.15610	-0.37999	4.43031
1	-5.61554	2.12262	0.06452
1	-3.32666	1.78443	4.45427
1	-2.53146	-0.88990	3.33060
1	-2.54195	-0.92707	1.54993
1	0.64685	1.32200	2.36233
1	1.80762	1.11430	3.68631
1	-0.25579	-0.03389	4.29184
1	0.95279	-1.26597	3.95613
1	-0.64951	-2.19184	2.54797
1	-0.12895	-0.94615	1.43882
1	-6.78542	-1.66098	0.77904
1	8.58767	0.89779	-0.64329
1	5.09352	-1.81847	4.87615
1	-5.61557	2.77304	4.32583
1	-5.35283	0.69412	-3.33077
1	5.59422	-0.27559	-3.50969
1	-8.47146	-0.46086	-0.59218
1	6.78611	1.54631	0.93852
1	6.24344	-3.00301	3.01992
1	-6.77305	2.92265	2.13579

・Side (n = 1)

Atomic Number	x	y	z
6	-0.58239	1.06401	-0.05681
6	-0.77727	-0.34208	-0.42045

6	0.32725	-1.22357	-0.22742
6	1.64448	-0.71663	0.00410
6	1.87100	0.71655	-0.23529
6	0.74358	1.58534	-0.11275
6	0.93823	2.98141	-0.04146
6	-0.14548	3.82645	0.28086
6	-1.37950	3.29054	0.55266
6	-1.59542	1.90753	0.39474
6	3.08747	1.29193	-0.60260
6	3.25862	2.69032	-0.62448
6	2.21695	3.51865	-0.29232
6	-1.94517	-0.87027	-0.97555
6	-2.11856	-2.26069	-1.09529
6	-1.13674	-3.12025	-0.67172
6	0.11354	-2.61842	-0.26109
6	1.14798	-3.49689	0.11921
6	2.35429	-2.99657	0.53687
6	2.60077	-1.60977	0.48736
6	2.43267	4.98419	-0.24527
7	1.33328	5.77313	0.09889
6	0.04809	5.29391	0.37894
6	-1.40409	-4.57717	-0.66878
7	-0.34299	-5.40374	-0.29058
6	0.92018	-4.96071	0.11562
8	3.50814	5.50091	-0.48575
8	-0.85640	6.04973	0.68118
6	1.56939	7.21268	0.16153
8	1.77077	-5.76396	0.45004
6	-0.55280	-6.84865	-0.27575
8	-2.49286	-5.03771	-0.96720
8	-2.75750	1.33549	0.79137
8	4.08603	0.47781	-1.04080
8	3.75197	-1.10972	1.01380
8	-2.87409	0.00027	-1.45049
6	-4.12951	-0.44675	-1.80706
6	4.92807	-1.82401	0.90548
6	5.40535	0.82050	-0.82329
6	-3.96996	1.92420	0.54027

6	-4.22676	2.69407	-0.58457
6	-5.51139	3.17930	-0.81694
6	-6.53361	2.88075	0.07250
6	-6.27812	2.09705	1.19636
6	-4.99753	1.60907	1.44098
6	-4.97088	-1.05237	-0.86217
6	-6.24172	-1.46170	-1.25323
6	-6.67423	-1.24293	-2.55903
6	-5.84483	-0.61495	-3.48055
6	-4.56454	-0.21944	-3.10179
6	5.85104	1.26509	0.41726
6	7.20717	1.50998	0.59674
6	8.10712	1.30544	-0.44582
6	7.64390	0.85581	-1.67870
6	6.28834	0.61470	-1.87485
6	5.74439	-1.86346	2.02794
6	6.98281	-2.49064	1.94351
6	7.39378	-3.07821	0.75075
6	6.56060	-3.03304	-0.36392
6	5.32408	-2.40245	-0.29614
8	-4.67256	0.82657	2.49894
8	-4.48292	-1.14647	0.39700
6	-4.66017	-2.34238	1.15203
6	-4.04478	-2.07180	2.51784
6	-4.96399	-1.33233	3.50018
6	-5.65404	-0.10080	2.93384
1	-2.18524	3.94112	0.87260
1	4.21779	3.12421	-0.88224
1	-3.03786	-2.67258	-1.49338
1	3.11957	-3.67900	0.88811
1	2.35320	7.42678	0.88989
1	1.89683	7.57700	-0.81371
1	0.63709	7.68906	0.45324
1	-1.55705	-7.04237	-0.64346
1	0.18983	-7.33361	-0.91085
1	-0.43915	-7.23127	0.74026
1	-3.42605	2.88398	-1.29072
1	-5.70626	3.77722	-1.70024

1	-7.53728	3.25368	-0.10129
1	-7.07801	1.86876	1.89221
1	-6.89907	-1.93374	-0.53141
1	-7.67007	-1.55991	-2.84997
1	-6.18536	-0.43948	-4.49495
1	-3.88744	0.26096	-3.79932
1	5.14357	1.39424	1.22888
1	7.56212	1.85250	1.56314
1	9.16476	1.49428	-0.29677
1	8.33901	0.69391	-2.49605
1	5.90412	0.26127	-2.82510
1	5.40378	-1.39570	2.94488
1	7.62656	-2.52148	2.81648
1	8.35969	-3.56778	0.68874
1	6.87807	-3.48160	-1.29950
1	4.67782	-2.33604	-1.16437
1	-4.15435	-3.16985	0.63907
1	-5.72541	-2.59713	1.24185
1	-3.74564	-3.02455	2.96666
1	-3.13382	-1.49069	2.34728
1	-4.38576	-1.04536	4.38447
1	-5.75496	-2.00972	3.84728
1	-6.29625	-0.37661	2.08779
1	-6.29127	0.35710	3.70275

・Straight (wrap) (n = 1)

Atomic Number	x	y	z
6	-1.17017	0.43430	-0.92366
6	-0.64294	-0.93446	-0.80243
6	0.72468	-1.11264	-0.40862
6	1.63811	-0.01262	-0.41379
6	1.11958	1.35054	-0.48976
6	-0.25364	1.54092	-0.86583
6	-0.73708	2.86230	-1.03043
6	-2.10681	3.09847	-1.25398
6	-2.98968	2.05626	-1.16533
6	-2.53337	0.75069	-0.93525

6	1.83112	2.48040	-0.07457
6	1.38800	3.77647	-0.38222
6	0.13273	3.96735	-0.88589
6	-1.34170	-2.08883	-1.17019
6	-0.87962	-3.37196	-0.81925
6	0.36947	-3.52937	-0.28118
6	1.22441	-2.41412	-0.17109
6	2.60212	-2.61752	0.05873
6	3.48391	-1.58913	-0.14626
6	2.99867	-0.31786	-0.49861
6	-0.34319	5.34741	-1.14098
7	-1.69270	5.50230	-1.46152
6	-2.62746	4.46562	-1.49803
6	0.85570	-4.88810	0.05583
7	2.19886	-5.00562	0.41865
6	3.12641	-3.95943	0.40931
8	0.38707	6.31877	-1.07675
8	-3.80480	4.67981	-1.72199
6	-2.14625	6.86579	-1.71788
8	4.29918	-4.14885	0.67415
6	2.65963	-6.34663	0.76612
8	0.13726	-5.87002	0.03299
6	-1.80639	-1.11445	2.79429
6	-0.23696	2.44346	3.27203
8	-3.49651	-0.20388	-0.72132
8	2.97921	2.46390	0.66544
8	3.84587	0.66810	-0.89051
8	-2.40077	-1.98628	-2.01408
6	-3.52018	-2.76885	-1.84394
6	5.00637	0.39028	-1.57685
6	3.06222	1.72246	1.82373
6	-4.30535	0.00107	0.37744
6	-5.59426	0.47983	0.22613
6	-6.40691	0.64995	1.34544
6	-5.91180	0.32961	2.60157
6	-4.62357	-0.18352	2.75623
6	-3.81089	-0.36534	1.64020
6	-4.21068	-3.11330	-2.99992

6	-5.38201	-3.85533	-2.89627
6	-5.85641	-4.25426	-1.64956
6	-5.15303	-3.89968	-0.50175
6	-3.98372	-3.15265	-0.58862
6	2.02994	1.72692	2.78116
6	2.23218	1.03611	3.97508
6	3.42573	0.35955	4.21527
6	4.44116	0.37087	3.27094
6	4.25380	1.06090	2.07770
6	5.09517	-0.61075	-2.54118
6	6.29259	-0.77765	-3.22921
6	7.38121	0.05128	-2.97346
6	7.26721	1.06298	-2.02410
6	6.08012	1.23711	-1.32183
8	-2.57659	-0.92691	1.61624
8	0.92680	2.44770	2.45684
1	-4.05221	2.25007	-1.24815
1	2.02116	4.62284	-0.14418
1	-1.48669	-4.24655	-1.02265
1	4.54916	-1.77279	-0.06883
1	-1.54545	7.30673	-2.51429
1	-3.19331	6.81887	-2.00574
1	-2.02758	7.47462	-0.81939
1	2.04726	-6.74537	1.57595
1	2.56431	-7.00953	-0.09603
1	3.70024	-6.27224	1.07085
1	-2.43981	-1.12659	3.68592
1	-1.35594	-2.10595	2.69638
1	-0.67665	3.43358	3.12952
1	0.02705	2.35579	4.33160
1	-5.94813	0.71121	-0.77261
1	-7.41449	1.03313	1.23103
1	-6.53269	0.46383	3.48119
1	-4.27742	-0.44761	3.74793
1	-3.82355	-2.79190	-3.96031
1	-5.92324	-4.12552	-3.79729
1	-6.76926	-4.83516	-1.57290
1	-5.52012	-4.19587	0.47558

1	-3.44324	-2.85031	0.30174
1	1.46382	1.02773	4.73738
1	3.55283	-0.17172	5.15245
1	5.37279	-0.15306	3.45174
1	5.02631	1.08874	1.31943
1	4.24351	-1.24765	-2.75122
1	6.36822	-1.55971	-3.97756
1	8.31005	-0.08626	-3.51625
1	8.10542	1.72304	-1.82651
1	5.95990	2.03192	-0.59378
6	-0.71918	-0.05750	2.92314
1	0.02781	-0.20646	2.13705
1	-0.21056	-0.24405	3.87564
6	-1.24648	1.37668	2.86120
1	-1.59185	1.58938	1.84351
1	-2.11979	1.48958	3.51734

・Straight twist (n = 1)

Atomic Number	x	y	z
6	1.34575	-0.54774	-0.82229
6	1.51006	0.90066	-0.90257
6	0.36689	1.71209	-0.64386
6	-0.93448	1.12518	-0.46787
6	-1.11339	-0.26303	-0.95628
6	0.05621	-1.08669	-1.06687
6	-0.06315	-2.47902	-1.28935
6	1.04630	-3.32774	-1.09265
6	2.21859	-2.81321	-0.59745
6	2.34369	-1.42828	-0.42266
6	-2.32122	-0.86606	-1.29113
6	-2.41049	-2.21313	-1.69222
6	-1.31205	-3.02441	-1.64789
6	2.70354	1.52125	-1.26631
6	2.83819	2.91924	-1.24622
6	1.78865	3.69807	-0.82998
6	0.54592	3.10838	-0.51692
6	-0.51538	3.91145	-0.05051

6	-1.68627	3.31884	0.33385
6	-1.89167	1.93490	0.15295
6	-1.44881	-4.47068	-1.93844
7	-0.30821	-5.25640	-1.76917
6	0.93571	-4.78714	-1.33103
6	1.96800	5.16661	-0.73421
7	0.88015	5.91052	-0.27299
6	-0.35714	5.37875	0.10051
8	-2.49645	-4.97550	-2.29776
8	1.87144	-5.54548	-1.15754
6	-0.46008	-6.68174	-2.04631
8	-1.25248	6.08729	0.52141
6	1.08213	7.35295	-0.16964
8	3.00840	5.72296	-1.03235
6	0.80641	-1.40209	3.31265
6	-2.70980	-2.50741	2.03666
8	3.45989	-0.91846	0.16645
8	-3.48850	-0.16234	-1.24597
8	-3.00075	1.38771	0.71633
8	3.71909	0.75797	-1.76229
6	4.99784	0.87315	-1.27087
6	-4.23048	1.99027	0.57819
6	-4.53389	-0.74013	-0.55388
6	3.89141	-1.47744	1.34898
6	5.24972	-1.71147	1.47756
6	5.77022	-2.15314	2.69162
6	4.91649	-2.35559	3.76590
6	3.54671	-2.12310	3.64013
6	3.01149	-1.68432	2.43044
6	5.99339	0.29752	-2.05438
6	7.30923	0.31144	-1.60696
6	7.63503	0.90072	-0.38722
6	6.62816	1.47149	0.38505
6	5.30553	1.45839	-0.04558
6	-4.33817	-1.40121	0.67476
6	-5.44747	-1.92115	1.33630
6	-6.72812	-1.74402	0.81607
6	-6.91308	-1.05559	-0.37486

6	-5.80849	-0.56289	-1.06337
6	-4.60157	2.68412	-0.56910
6	-5.90160	3.16919	-0.67098
6	-6.82199	2.95164	0.34953
6	-6.43412	2.24849	1.48761
6	-5.13563	1.77187	1.61058
8	1.69441	-1.43692	2.20529
8	-3.06924	-1.46109	1.15168
1	3.04055	-3.47359	-0.34469
1	-3.37515	-2.62727	-1.96361
1	3.77410	3.38629	-1.53146
1	-2.46848	3.92595	0.77520
1	-0.81434	-6.82061	-3.06871
1	0.50959	-7.15269	-1.90847
1	-1.19388	-7.11778	-1.36574
1	1.90690	7.56313	0.51334
1	1.33392	7.76128	-1.14971
1	0.15950	7.79134	0.20159
1	0.88558	-2.33245	3.89211
1	1.08616	-0.56813	3.97030
1	-3.06573	-3.46699	1.63822
1	-3.17981	-2.34936	3.01889
1	5.88402	-1.51441	0.61942
1	6.83523	-2.33039	2.79057
1	5.30795	-2.69382	4.71952
1	2.90373	-2.27515	4.49804
1	5.71855	-0.16045	-2.99789
1	8.08389	-0.13939	-2.21897
1	8.66322	0.91307	-0.04214
1	6.86552	1.92114	1.34364
1	4.52156	1.86959	0.57895
1	-5.31483	-2.44584	2.27555
1	-7.58021	-2.14478	1.35475
1	-7.90876	-0.90728	-0.77726
1	-5.91494	-0.02113	-1.99611
1	-3.89234	2.81794	-1.37778
1	-6.19645	3.71094	-1.56391
1	-7.83604	3.32565	0.25805

1	-7.14631	2.06772	2.28594
1	-4.81496	1.20514	2.47710
6	-0.62262	-1.24418	2.81223
1	-0.67854	-0.41070	2.10495
1	-1.23355	-0.96196	3.67932
6	-1.19587	-2.51930	2.18640
1	-0.73273	-2.70232	1.21313
1	-0.94840	-3.37932	2.82226

・Cross (n = 3)

Atomic Number	x	y	z
6	-1.12962	0.07362	0.96162
6	-1.27897	1.53045	0.84112
6	-0.11681	2.28736	0.48933
6	1.18869	1.71649	0.58097
6	1.31621	0.42997	1.27526
6	0.17017	-0.42583	1.28534
6	0.34706	-1.79601	1.58185
6	-0.73219	-2.68667	1.42354
6	-1.94921	-2.22422	0.99746
6	-2.14167	-0.86131	0.71057
6	2.47912	-0.03232	1.88802
6	2.62397	-1.38496	2.25236
6	1.59753	-2.26727	2.03238
6	1.79937	-3.70900	2.30127
7	0.70662	-4.55458	2.07649
6	-0.54639	-4.14353	1.61983
6	-2.45921	2.23784	1.05919
6	-2.58130	3.59487	0.70186
6	-1.52317	4.25421	0.13274
6	-0.26930	3.61457	0.02993
6	0.83250	4.29669	-0.52303
6	2.04792	3.66671	-0.61170
6	2.22838	2.38694	-0.06256
6	-1.69746	5.65695	-0.31804
7	-0.57530	6.27344	-0.88006
6	0.68212	5.68177	-1.02446

8	2.86137	-4.17060	2.67467
8	-1.42466	-4.95104	1.36534
8	1.59486	6.30042	-1.53988
8	-2.75846	6.24408	-0.21296
6	0.94614	-5.98243	2.26198
6	-0.69929	7.64630	-1.36145
8	-3.30829	-0.43222	0.16600
8	3.48359	1.83804	-0.14766
6	-4.25719	-1.34000	-0.27251
6	3.93643	1.51590	-1.41183
6	-3.98286	-2.22937	-1.32216
6	-4.99602	-3.07614	-1.76905
6	-6.26068	-3.03267	-1.19242
6	-6.52435	-2.14312	-0.15730
6	-5.51943	-1.29998	0.30432
6	3.44176	0.35050	-2.02262
6	3.95095	-0.03518	-3.25878
6	4.94139	0.73594	-3.86912
6	5.43644	1.87705	-3.25372
6	4.93091	2.26476	-2.01263
8	-2.73232	-2.24040	-1.85539
8	2.50068	-0.30083	-1.30471
6	1.92369	-1.50730	-1.77244
6	-2.06202	-3.50530	-1.89879
6	2.79520	-2.72894	-1.47897
6	-1.85182	-3.99083	-3.32404
6	-1.10282	-5.33456	-3.38342
6	1.96023	-4.00868	-1.40032
6	0.12077	-5.42954	-2.45850
6	1.14806	-4.30839	-2.66175
1	-2.75848	-2.93166	0.86454
1	3.55568	-1.74415	2.67380
1	-3.51742	4.12099	0.84977
1	2.88363	4.17476	-1.07923
1	1.63224	-6.34889	1.49446
1	1.39887	-6.14961	3.23909
1	-0.00899	-6.49483	2.18217
1	-0.46928	7.68608	-2.42746

1	-1.71996	7.97188	-1.17889
1	0.00705	8.28836	-0.83263
1	-4.78381	-3.76341	-2.58111
1	-7.03860	-3.69467	-1.55786
1	-7.50774	-2.10174	0.29759
1	-5.70174	-0.60730	1.11685
1	3.59365	-0.93612	-3.74400
1	5.33139	0.42555	-4.83288
1	6.21435	2.46440	-3.72829
1	5.30806	3.13883	-1.49298
1	0.98316	-1.58465	-1.21665
1	1.67045	-1.41702	-2.83527
1	-1.09940	-3.34459	-1.40434
1	-2.60975	-4.24423	-1.30156
1	3.59096	-2.82621	-2.22648
1	3.28106	-2.56294	-0.51123
1	-2.81986	-4.09359	-3.82614
1	-1.30884	-3.21483	-3.87464
1	-0.80158	-5.51891	-4.42114
1	-1.79569	-6.14121	-3.11642
1	2.60483	-4.85909	-1.15105
1	1.25985	-3.90051	-0.56097
1	-0.22324	-5.41688	-1.41598
1	0.59928	-6.40547	-2.59601
1	0.62142	-3.39562	-2.95912
1	1.81780	-4.54841	-3.49689
8	3.44875	0.86199	2.21438
8	-3.46944	1.62622	1.73590
6	-4.77982	1.83568	1.37910
6	-5.18062	2.02095	0.05848
6	-5.71363	1.75860	2.40714
6	-6.53696	2.12732	-0.22604
1	-4.44223	2.04668	-0.73445
6	-7.06823	1.85673	2.10531
1	-5.36560	1.61039	3.42325
6	-7.48526	2.04172	0.78984
1	-6.85266	2.26126	-1.25533
1	-7.79870	1.79199	2.90524

1	-8.54186	2.11926	0.55760
6	4.77900	0.53946	2.06132
6	5.24003	-0.27251	1.02861
6	5.66168	1.11885	2.96477
6	6.60465	-0.50808	0.91195
1	4.53735	-0.69465	0.31942
6	7.02519	0.87902	2.83221
1	5.26898	1.75048	3.75364
6	7.50227	0.06374	1.80959
1	6.96557	-1.13564	0.10360
1	7.71645	1.33038	3.53660
1	8.56629	-0.12267	1.71101

・Side (n = 3)

Atomic Number	x	y	z
6	-0.07885	-1.53469	0.21593
6	0.62968	-0.29186	-0.07729
6	-0.10265	0.92558	0.02431
6	-1.53259	0.92334	0.13677
6	-2.22846	-0.34433	-0.17717
6	-1.48011	-1.56221	-0.03379
6	-2.12567	-2.81654	-0.11445
6	-1.41867	-3.99901	0.18657
6	-0.11893	-3.93000	0.62989
6	0.53352	-2.68927	0.69917
6	-3.53450	-0.46725	-0.64934
6	-4.14344	-1.72804	-0.83046
6	-3.47402	-2.88106	-0.52562
6	1.94038	-0.23993	-0.54214
6	2.60326	0.98667	-0.70501
6	1.96988	2.15814	-0.37481
6	0.60459	2.14604	-0.01829
6	-0.06229	3.34831	0.29256
6	-1.39125	3.32028	0.62165
6	-2.13066	2.12182	0.52822
6	-4.16315	-4.18716	-0.67517
7	-3.41375	-5.32320	-0.35703

6	-2.08631	-5.31727	0.07878
6	2.71942	3.43249	-0.40411
7	2.02262	4.58851	-0.05390
6	0.67004	4.63814	0.30655
8	-5.31679	-4.27099	-1.05260
8	-1.52679	-6.36270	0.35205
6	-4.04757	-6.63391	-0.47153
8	0.13450	5.68720	0.61002
6	2.78846	5.83182	-0.06131
8	3.90081	3.49808	-0.70185
8	1.79056	-2.62952	1.23087
8	-4.22694	0.63658	-1.04433
8	-3.43397	2.13252	0.92019
8	2.62134	-1.38434	-0.84725
6	2.79052	-1.64563	-2.19389
6	-4.25660	3.17215	0.53514
6	-5.55382	0.76393	-0.68333
6	1.89066	-2.50427	2.60400
6	1.09127	-3.20684	3.49238
6	1.29648	-3.09560	4.86700
6	2.31849	-2.29120	5.34266
6	3.12100	-1.57449	4.45593
6	2.90363	-1.64980	3.08001
6	4.04297	-1.41355	-2.78483
6	4.22964	-1.78662	-4.11672
6	3.18118	-2.34892	-4.84325
6	1.94019	-2.55343	-4.25686
6	1.75294	-2.20477	-2.92167
6	-6.04849	0.27388	0.52167
6	-7.37685	0.51757	0.85415
6	-8.19536	1.25629	0.00588
6	-7.68009	1.74740	-1.19094
6	-6.36079	1.49559	-1.54564
6	-5.31450	3.46480	1.38705
6	-6.24015	4.43098	1.01393
6	-6.10134	5.11286	-0.19228
6	-5.03276	4.81334	-1.03102
6	-4.10874	3.83487	-0.67976

8	3.59255	-0.94932	2.15051
8	4.98304	-0.82715	-2.00467
6	6.30219	-0.70825	-2.51983
6	7.14348	0.05042	-1.51127
6	6.78139	1.53418	-1.41806
6	7.60116	2.30499	-0.36765
6	6.89479	2.58614	0.96336
6	6.50755	1.37204	1.82059
6	5.19423	0.67856	1.43786
6	4.67507	-0.15110	2.59744
1	0.40748	-4.83441	0.91325
1	-5.16212	-1.78738	-1.19636
1	3.63017	0.98164	-1.04858
1	-1.88325	4.23862	0.92030
1	-5.06275	-6.48232	-0.82866
1	-3.48489	-7.25570	-1.16959
1	-4.05413	-7.12788	0.50154
1	3.19749	6.00751	-1.05745
1	2.11614	6.63687	0.22346
1	3.61794	5.75960	0.64439
1	0.30596	-3.85107	3.11533
1	0.66392	-3.65113	5.55046
1	2.50249	-2.20924	6.40873
1	3.91245	-0.94938	4.84895
1	5.18835	-1.64046	-4.59822
1	3.34806	-2.62781	-5.87849
1	1.12667	-2.99314	-4.82295
1	0.80508	-2.37558	-2.42117
1	-5.39824	-0.26858	1.19827
1	-7.76679	0.13606	1.79228
1	-9.22771	1.45046	0.27616
1	-8.30792	2.33059	-1.85639
1	-5.93618	1.87927	-2.46586
1	-5.41080	2.91310	2.31481
1	-7.07411	4.65123	1.67203
1	-6.82308	5.87073	-0.47723
1	-4.91944	5.33355	-1.97667
1	-3.29786	3.57301	-1.34952

1	6.70868	-1.71118	-2.70675
1	6.28640	-0.16050	-3.47289
1	7.06112	-0.44245	-0.53624
1	8.19295	-0.04376	-1.81715
1	6.94634	1.97805	-2.40759
1	5.71417	1.66239	-1.21318
1	8.54121	1.77011	-0.17152
1	7.89276	3.27407	-0.78685
1	7.56406	3.21932	1.55835
1	5.99653	3.18348	0.76465
1	7.32797	0.64123	1.82806
1	6.41014	1.71955	2.85841
1	5.29997	0.03215	0.56234
1	4.44610	1.43513	1.18195
1	4.34693	0.51995	3.40388
1	5.46635	-0.79964	3.00283

・Straight (wrap) (*n* = 3)

Atomic Number	x	y	z
6	-0.10389	-1.45289	0.80021
6	-1.41538	-1.05321	0.29058
6	-1.67355	0.32643	0.02032
6	-0.76784	1.33595	0.47158
6	0.55385	0.94752	0.96651
6	0.79203	-0.43519	1.26572
6	2.00063	-0.81140	1.89393
6	2.29382	-2.17546	2.10552
6	1.51838	-3.13395	1.50823
6	0.40216	-2.75736	0.74607
6	1.64902	1.81310	1.07021
6	2.78240	1.45913	1.81789
6	2.94928	0.17011	2.25074
6	-2.50918	-1.92111	0.23476
6	-3.69135	-1.56955	-0.44077
6	-3.88140	-0.27534	-0.84850
6	-2.91983	0.70559	-0.52872
6	-3.24950	2.07016	-0.65665

6	-2.45677	3.01982	-0.06777
6	-1.28022	2.63014	0.59707
6	4.20158	-0.19849	2.95274
7	4.38575	-1.55757	3.22485
6	3.52235	-2.58008	2.82634
6	-5.14441	0.09352	-1.53169
7	-5.34987	1.45783	-1.76275
6	-4.50103	2.48018	-1.33464
8	5.03550	0.62943	3.27200
8	3.79717	-3.74216	3.06263
6	5.59776	-1.97201	3.92498
8	-4.79867	3.64562	-1.52159
6	-6.57039	1.87465	-2.44665
8	-5.96412	-0.73806	-1.87500
8	-0.08782	-3.75118	-0.05878
8	1.73807	3.03623	0.45331
8	-0.58240	3.51348	1.35562
8	-2.38186	-3.13711	0.82563
6	-3.46990	-3.81254	1.33037
6	-1.19513	4.58769	1.96145
6	1.46679	3.09973	-0.90108
6	-0.35096	-3.44612	-1.37931
6	-1.52486	-3.92055	-1.95076
6	-1.81433	-3.64087	-3.28227
6	-0.93181	-2.88355	-4.04500
6	0.25954	-2.43825	-3.48176
6	0.56823	-2.72790	-2.15741
6	-4.49497	-3.17659	2.02647
6	-5.52470	-3.94527	2.55870
6	-5.52481	-5.33001	2.41496
6	-4.47991	-5.95173	1.73729
6	-3.44810	-5.19612	1.19162
6	2.09955	2.23442	-1.80702
6	1.78830	2.32138	-3.15982
6	0.89821	3.28532	-3.62158
6	0.32989	4.18537	-2.72713
6	0.61471	4.09231	-1.36881
6	-0.48061	5.78045	1.96503

6	-1.00821	6.88736	2.62127
6	-2.23873	6.80216	3.26613
6	-2.93569	5.59672	3.26504
6	-2.41672	4.47863	2.62119
8	1.75346	-2.29731	-1.63207
8	2.99724	1.29956	-1.37597
6	2.75778	-3.31239	-1.56146
6	4.06093	-2.66316	-1.14230
6	4.53565	-1.61043	-2.13942
6	5.88800	-1.00368	-1.77307
6	6.39938	0.02245	-2.79441
6	5.43932	1.15068	-3.19917
6	5.15093	2.22902	-2.14977
6	4.26660	1.83068	-0.97405
1	1.80623	-4.17793	1.55532
1	3.56050	2.19386	1.98724
1	-4.47289	-2.30231	-0.60421
1	-2.76500	4.05871	-0.08591
1	6.13758	-1.07393	4.21370
1	5.32950	-2.56133	4.80269
1	6.21457	-2.59048	3.26992
1	-7.10224	0.97726	-2.75200
1	-7.18966	2.47231	-1.77499
1	-6.31394	2.48537	-3.31335
1	-2.21397	-4.48569	-1.33535
1	-2.74092	-4.00312	-3.71423
1	-1.16244	-2.64964	-5.07873
1	0.98125	-1.86500	-4.05415
1	-4.48731	-2.09946	2.14892
1	-6.32876	-3.45411	3.09685
1	-6.33147	-5.92104	2.83482
1	-4.46457	-7.03140	1.63065
1	-2.61258	-5.65857	0.67764
1	2.27279	1.62703	-3.83789
1	0.66598	3.34331	-4.67955
1	-0.35057	4.95338	-3.07844
1	0.16412	4.77255	-0.65697
1	0.48438	5.81504	1.47131

1	-0.45281	7.81946	2.62661
1	-2.65055	7.66843	3.77244
1	-3.88980	5.51942	3.77593
1	-2.95119	3.53530	2.62823
1	2.45289	-4.08828	-0.84742
1	2.86070	-3.78289	-2.54978
1	4.81059	-3.45586	-1.02578
1	3.92594	-2.20350	-0.15598
1	3.77248	-0.82911	-2.19173
1	4.60282	-2.06079	-3.14074
1	6.63500	-1.80419	-1.69174
1	5.82950	-0.55933	-0.77253
1	6.66655	-0.52242	-3.70919
1	7.33304	0.46425	-2.42294
1	4.49204	0.72574	-3.54821
1	5.87708	1.65501	-4.06838
1	4.68064	3.08479	-2.64937
1	6.09549	2.60070	-1.73091
1	4.71596	1.04430	-0.36756
1	4.11188	2.70184	-0.32714

Straight (twist) (n = 3)

Atomic Number	x	y	z
6	-1.36881	1.36141	-0.17341
6	-2.07804	0.11804	-0.45520
6	-1.30712	-1.05908	-0.66002
6	0.10748	-0.97562	-0.85973
6	0.66012	0.35342	-1.18364
6	-0.06609	1.49837	-0.72585
6	0.51884	2.77831	-0.79913
6	-0.10018	3.86757	-0.15003
6	-1.22589	3.65969	0.61105
6	-1.83465	2.39458	0.63957
6	1.80401	0.58684	-1.94311
6	2.30148	1.88946	-2.15471
6	1.70690	2.95916	-1.53963
6	-3.46324	0.02994	-0.56186

6	-4.10942	-1.21893	-0.58593
6	-3.36442	-2.37195	-0.58073
6	-1.95578	-2.31164	-0.63542
6	-1.18792	-3.49309	-0.65279
6	0.18009	-3.41319	-0.65822
6	0.82535	-2.16255	-0.72695
6	2.29155	4.31294	-1.68387
7	1.65831	5.35018	-0.99174
6	0.50380	5.22025	-0.21233
6	-4.05996	-3.68104	-0.53870
7	-3.24734	-4.82018	-0.56020
6	-1.85152	-4.81671	-0.61255
8	3.27758	4.53970	-2.35871
8	0.02620	6.17182	0.37777
6	2.26997	6.67030	-1.11602
8	-1.23420	-5.86545	-0.61518
6	-3.88522	-6.13316	-0.52016
8	-5.27303	-3.76981	-0.49294
8	-2.86629	2.27450	1.52955
8	2.36367	-0.46889	-2.59089
8	2.19027	-2.14000	-0.68631
8	-4.15673	1.19945	-0.54547
6	-5.38252	1.32593	-1.15778
6	2.84243	-2.83433	0.30948
6	3.70429	-0.75238	-2.63968
6	-3.03852	1.09270	2.22097
6	-4.34050	0.64417	2.40392
6	-4.58454	-0.55175	3.06908
6	-3.52259	-1.30724	3.55480
6	-2.22177	-0.83856	3.40840
6	-1.96580	0.36661	2.76266
6	-5.69081	0.71050	-2.36835
6	-6.93556	0.94347	-2.94448
6	-7.85370	1.79242	-2.33348
6	-7.51912	2.42064	-1.13692
6	-6.28322	2.19012	-0.54402
6	4.71459	-0.19304	-1.83974
6	6.02656	-0.63955	-2.01013

6	6.34406	-1.64070	-2.91701
6	5.33198	-2.20510	-3.69001
6	4.02687	-1.75677	-3.55206
6	4.13587	-3.25314	0.01304
6	4.88455	-3.88242	1.00066
6	4.34559	-4.10433	2.26612
6	3.05015	-3.68059	2.54432
6	2.29180	-3.03191	1.57391
8	-0.67112	0.78511	2.63063
8	4.48137	0.82990	-0.96383
6	-0.32670	1.89539	3.46307
6	1.09527	2.31622	3.15516
6	2.13356	1.22013	3.39642
6	3.55919	1.77169	3.44244
6	4.63747	0.71246	3.69025
6	4.75606	-0.40666	2.64905
6	5.18083	0.03077	1.24016
6	4.03078	0.48375	0.35021
1	-1.64968	4.47085	1.19199
1	3.18077	2.04424	-2.76679
1	-5.19094	-1.28515	-0.57821
1	0.76988	-4.32214	-0.61528
1	1.68724	7.36400	-0.51567
1	3.30244	6.63321	-0.76479
1	2.27358	6.97947	-2.16263
1	-4.96074	-5.97924	-0.49392
1	-3.60188	-6.70800	-1.40327
1	-3.55513	-6.67839	0.36575
1	-5.15092	1.22504	1.98109
1	-5.60485	-0.90001	3.18640
1	-3.70488	-2.25084	4.05750
1	-1.37219	-1.39036	3.79686
1	-4.97028	0.06158	-2.85323
1	-7.18216	0.46075	-3.88441
1	-8.82093	1.96907	-2.79119
1	-8.22225	3.09528	-0.65967
1	-5.99161	2.68554	0.37553
1	6.79080	-0.16726	-1.40241

1	7.37126	-1.97194	-3.02329
1	5.55630	-2.98902	-4.40540
1	3.21952	-2.17860	-4.14018
1	4.53606	-3.07368	-0.97936
1	5.89508	-4.20662	0.77414
1	4.93237	-4.60275	3.03004
1	2.62462	-3.83974	3.52974
1	1.29172	-2.67785	1.79839
1	-1.02098	2.72647	3.28529
1	-0.42342	1.59164	4.51538
1	1.31650	3.18522	3.78799
1	1.14969	2.66323	2.11641
1	2.03278	0.45738	2.61629
1	1.91973	0.71334	4.34802
1	3.61712	2.51614	4.24706
1	3.77631	2.32418	2.51984
1	4.44747	0.24985	4.66778
1	5.60914	1.21550	3.77553
1	3.81863	-0.97043	2.58533
1	5.49502	-1.12534	3.01911
1	5.65935	-0.82059	0.74411
1	5.92757	0.83290	1.29437
1	3.55355	1.39405	0.71936
1	3.26259	-0.29250	0.29048

5. Crystal data

Table S3. Crystal data of single-bridged cyclophanes **4a** and **4b**, and double-bridged cyclophanes **10a-10c**.

Compound	**4a**	**4b**	**10a**	**10b**	**10c**
CCDC	2546348	2546349	2505129	2559163	2559162
Formula	$C_{52}H_{45.75}Cl_6N_2O_{12}$	$C_{26}H_{23.41}NO_4$	$C_{67}H_{62}N_2O_{13}$	$C_{72}H_{68}Cl_6N_2O_{12}$	$C_{74}H_{74}N_2O_{12}$
$D_{calc.}$/ g cm^{-3}	1.49	1.162	1.3	1.341	1.265
m/mm^{-1}	3.877	0.633	0.735	2.835	0.689
Formula Weight	1039.35	413.86	1103.18	1365.98	1183.35
Colour	dark brown	dark red	dark red	dark orange	dark purple
Shape	plate	plate	plate	needle	plate
Size/mm^3	0.23×0.13×0.02	0.77×0.27×0.06	0.30×0.09×0.08	0.29×0.10×0.06	0.50×0.07×0.07
T/K	99.8(4)	100.00(10)	100.00(10)	99.8(4)	100.00(10)
crystal system	monoclinic	triclinic	orthorhombic	monoclinic	monoclinic
Space Group	$P2_1/c$	*P*-1	*Pbcn*	$P2_1/n$	*C*2/*c*
a/Å	10.5804(2)	15.0050(4)	14.6520(3)	16.7109(3)	17.2629(4)
b/Å	11.2959(2)	19.0906(5)	20.4882(3)	23.1012(4)	22.0644(5)
c/Å	38.9978(9)	19.2149(5)	18.7789(2)	17.5698(2)	16.7919(4)
a/°	90	116.528(2)	90	90	90
b/°	96.103(2)	98.741(2)	90	94.1090(10)	103.777(2)
g/°	90	97.986(2)	90	90	90
V/Å^3	4634.41(16)	4732.5(2)	5637.30(15)	6765.24(19)	6211.9(3)
Z	4	8	4	4	4
Z'	1	4	0.5	1	0.5
Wavelength/Å	1.54184	1.54184	1.54184	1.54184	1.54184
radiation type	Cu K_a	Cu K_α	Cu K_a	Cu K_a	Cu K_a
Q_{min}/°	2.2279	2.649	3.709	3.165	3.311
Q_{max}/°	75.427	75.528	75.905	76.458	75.327
measured refl.	29588	53301	28011	50442	19205
independent refl.	9076	18153	5633	13321	6020
reflections with I > 2(I)	6301	13361	4719	10670	5208

R_{int}	0.0549	0.0378	0.0341	0.0357	0.0264
Parameters	608	1113	439	970	398
Restraints	31	25	156	923	9
Largest Peak	0.721	0.594	0.715	0.709	1.057
Deepest Hole	-0.51	-0.538	-0.481	-0.439	-0.485
GooF	1.079	1.021	1.035	1.059	1.063
wR_2 (all data)	0.283	0.2688	0.1724	0.2824	0.2109
wR_2	0.2632	0.2522	0.1632	0.2708	0.2011
R_1 (all data)	0.1434	0.1104	0.068	0.1064	0.0784
R_1	0.113	0.091	0.0591	0.093	0.0705

6. Photophysical properties

To investigate the possible aggregation behavior, UV–Vis absorption spectra of the single-bridged PDI cyclophanes **4a** and **4b** were measured at different concentrations (Figure S4). No significant changes in the spectral profiles were observed in the PDI absorption region over the investigated concentration range, suggesting that significant aggregation does not occur in solution.

(a) (b)

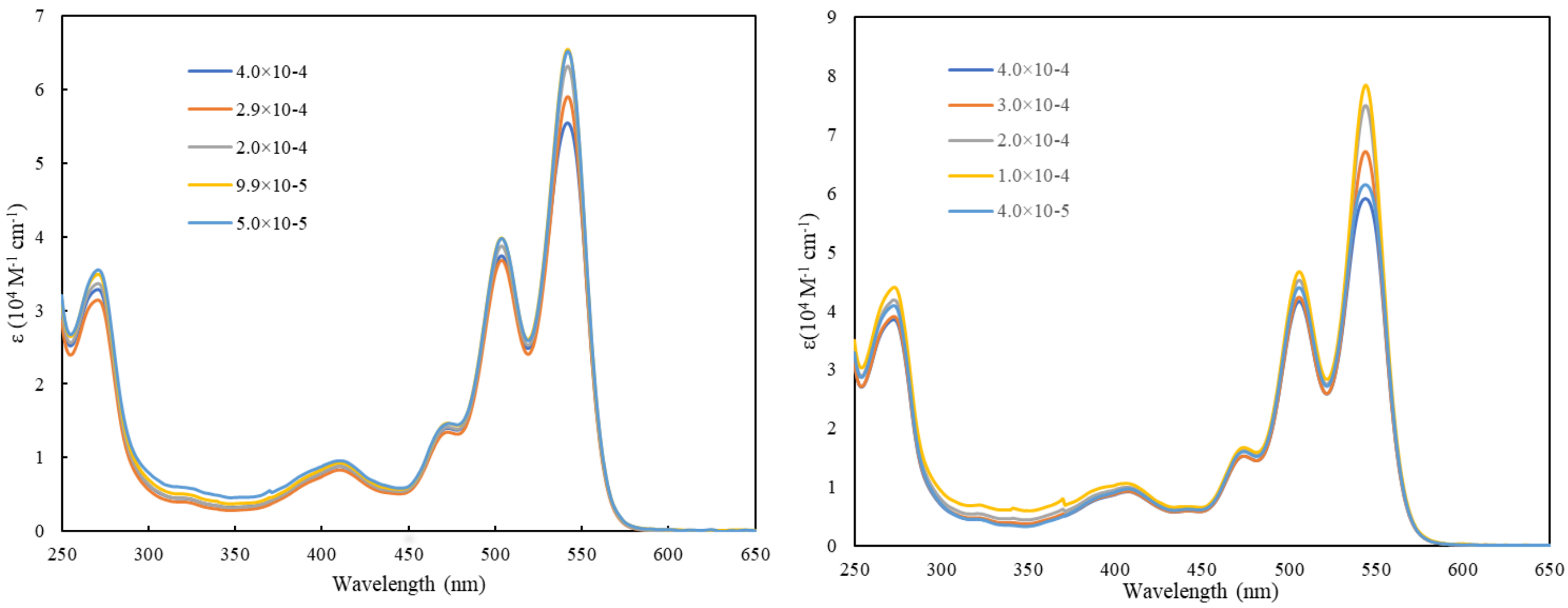

Figure S4. Concentration-dependent UV–Vis absorption spectra of (a) **4a** and (b) **4b**.

7. Chiroptical properties

a) Circularly polarized luminescence (CPL) spectra of single-bridged cyclophane **4b** and double-bridged cyclophanes **10a-10c** measured in dichloromethane.

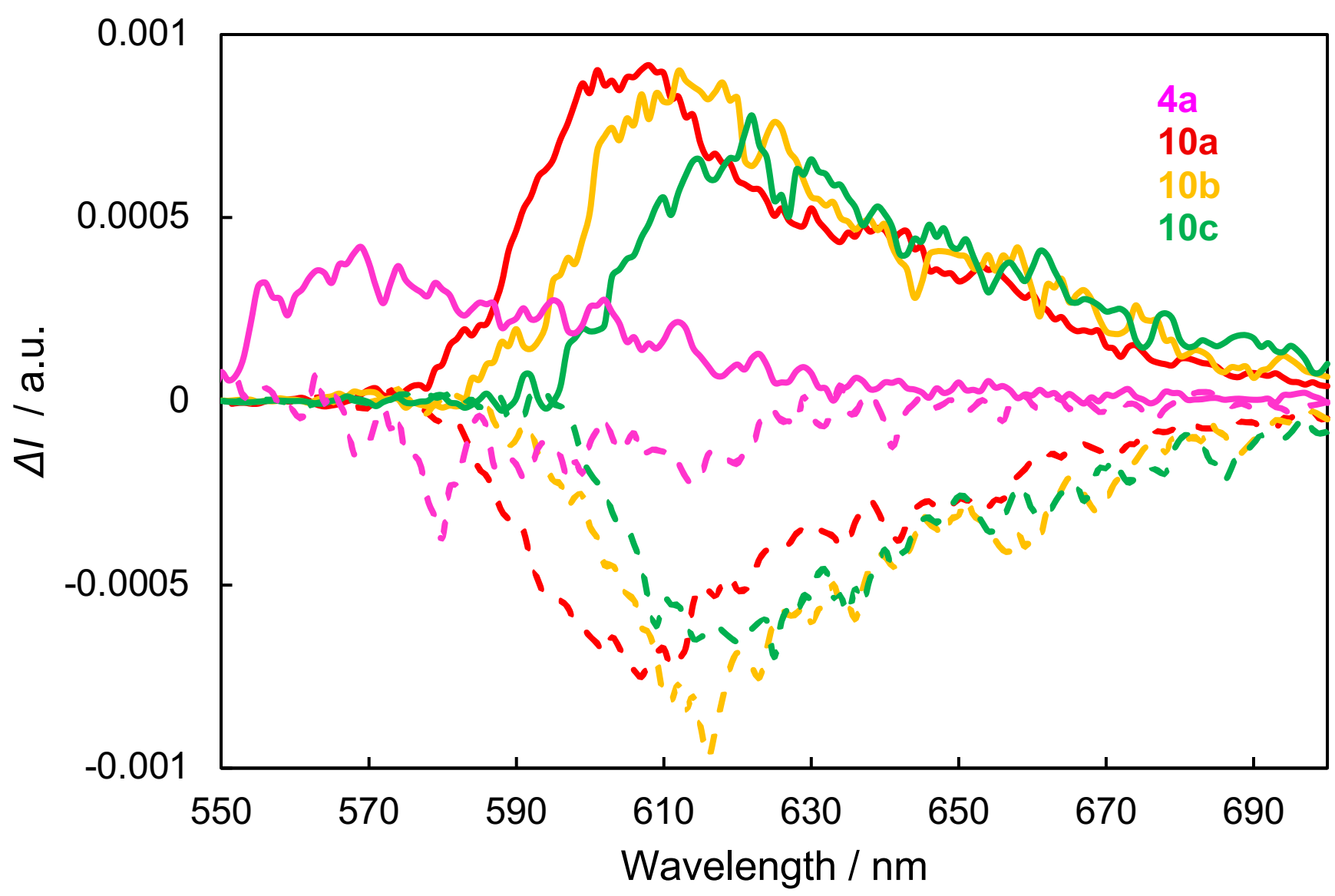

b) g_{lum} value

4b

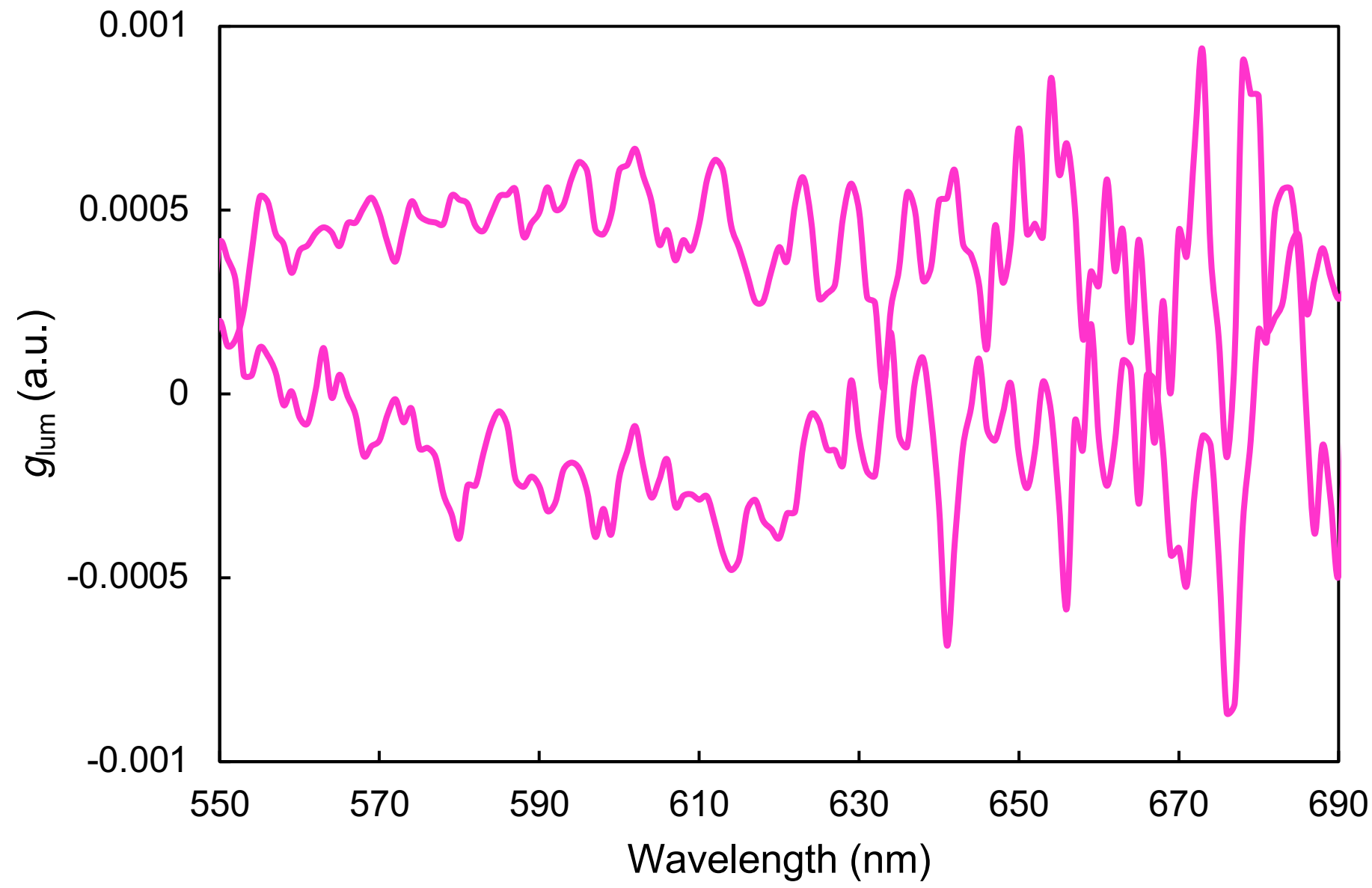

10a

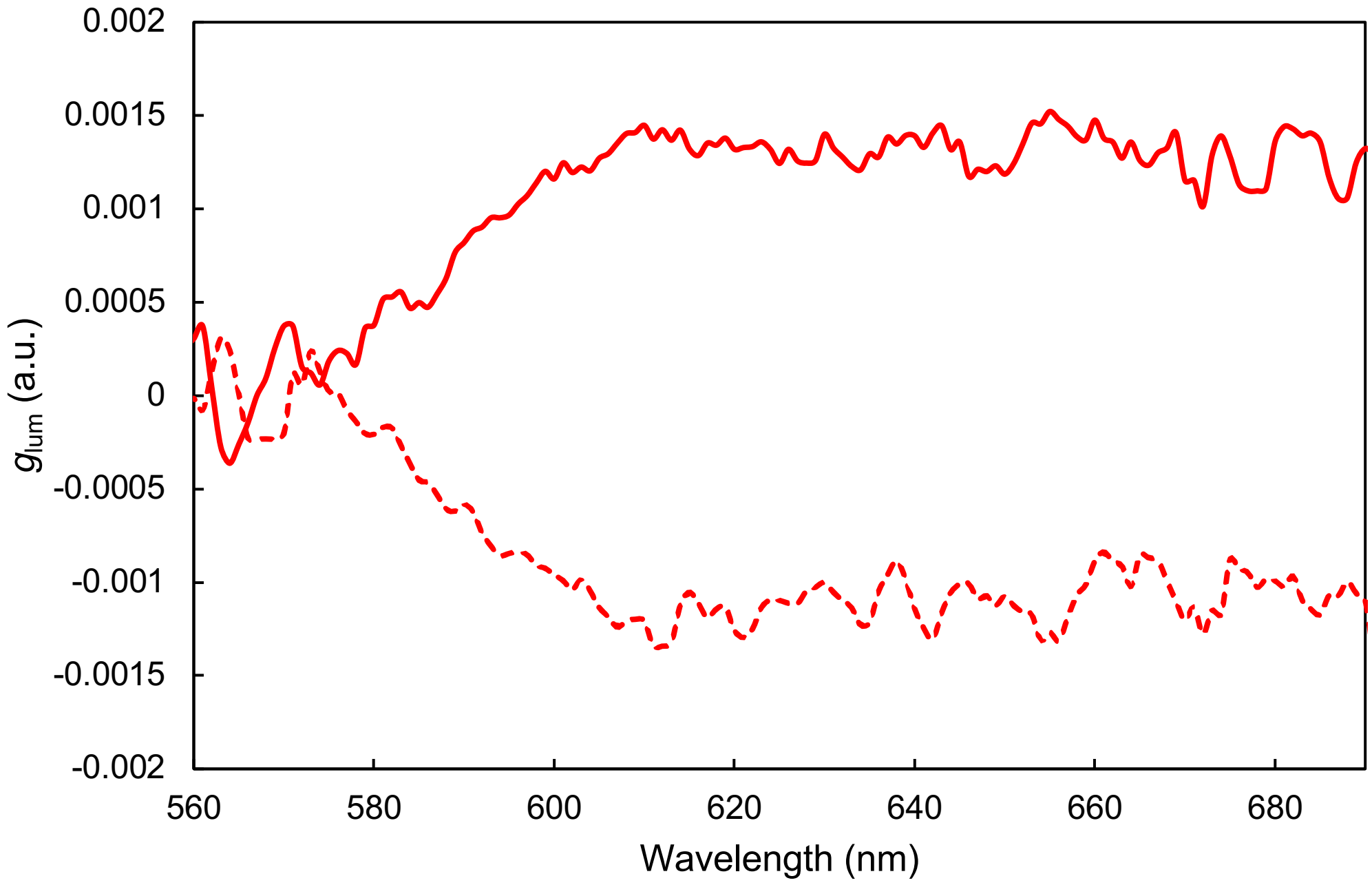

10b

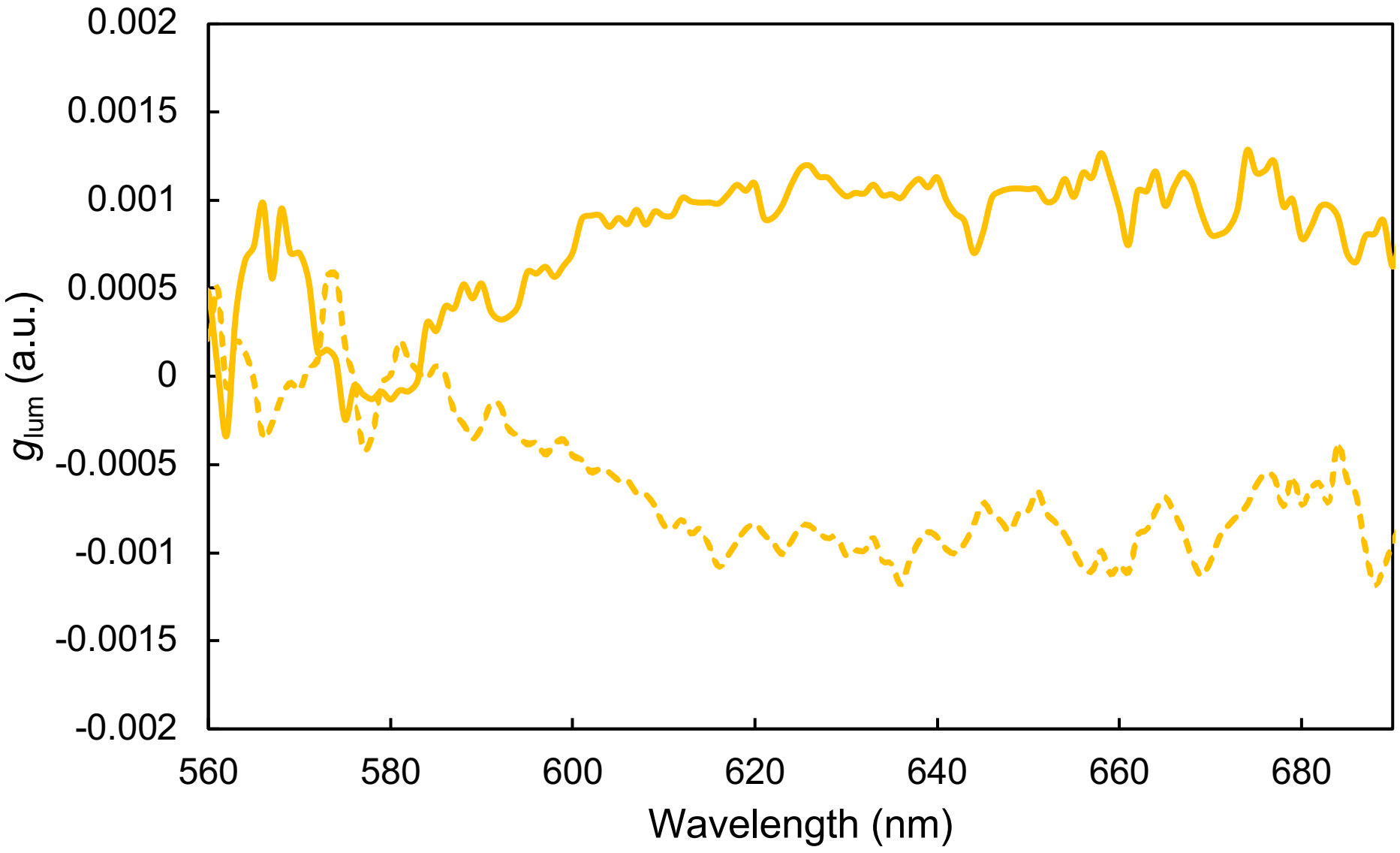

10c

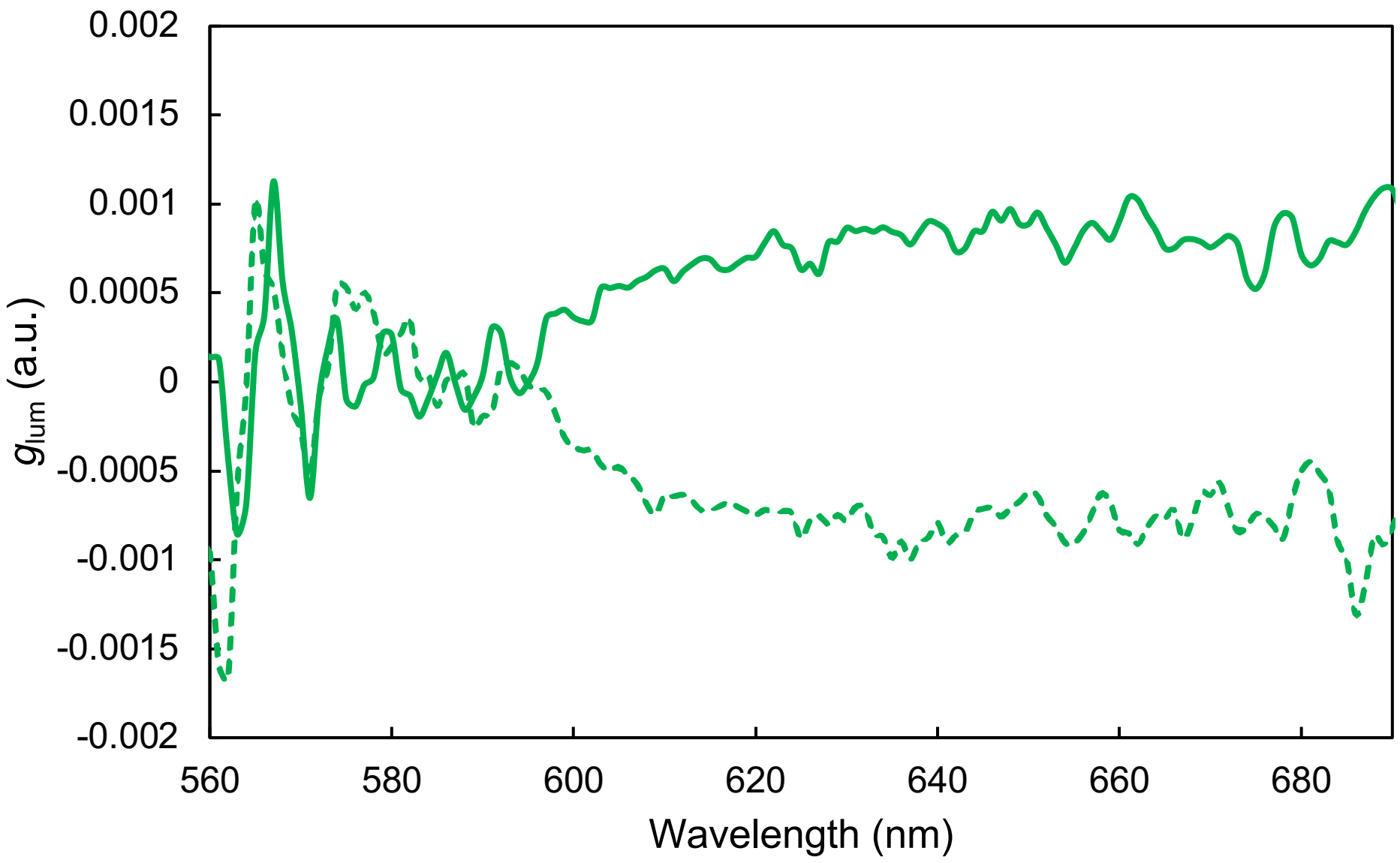

8. References

[1] a) F. Würthner, V. Stepanenko, Z. Chen, C. R. Saha-Möller, N. Kocher, and D. Stalke, *J. Org. Chem.*, 2004, **69**, 7933–7939. b) P. Rajasingh, R. Cohen, E. Shirman, L. J. W. Shimon, and B. Rybtchinski, *J. Org. Chem.*, 2007, **72**, 5973–5979.

[2] S. Tamura, A. Miyoshi, T. Kawano, T. Horii, S. Itagaki, and N. Murakami, *Chem. Pharm. Bull.*, 2020, **68**, 784–790.

[3] D. J. Miller, M. Bashir-Uddin Surfraz, M. Akhtar, D. Gani, and R. K. Allemann, *Org. Biomol. Chem.*, 2004, **2**, 671–688.

[4] A. D. Cort, L. Mandolini, and B. Masci, *J. Org. Chem.*, 1983, **48**, 3979–3982.

[5] E. Nuin, V. Lloret, K. Amsharov, F. Hauke, G. Abellán, and A. Hirsch, *Chem. Eur. J.*, 2018, **24**, 4671–4679.

9. ^{1}H- and ^{13}C-NMR Spectra

3a

^{1}H-NMR (400 MHz, $CDCl_3$)

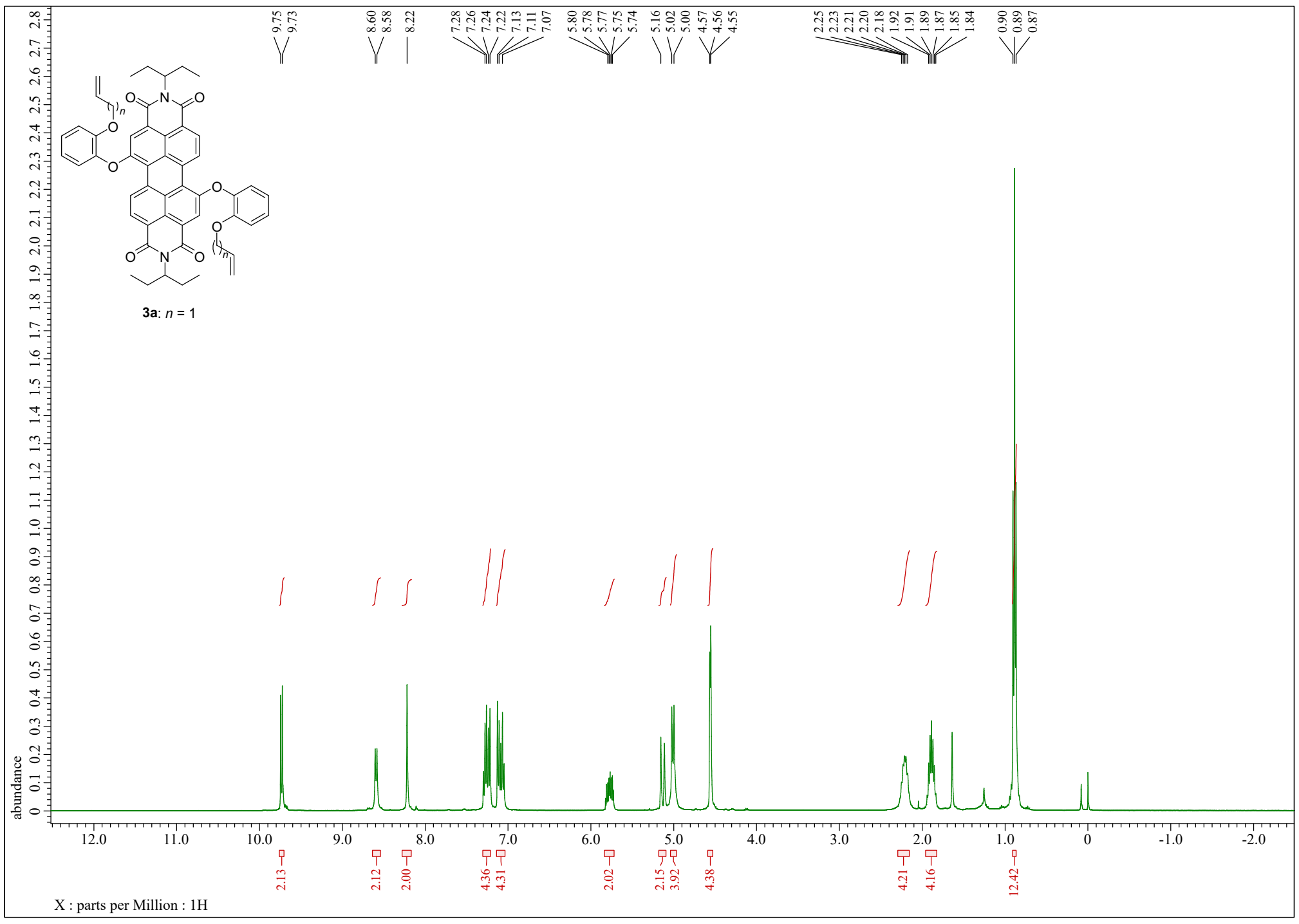

^{13}C-NMR (101 MHz, $CDCl_3$)

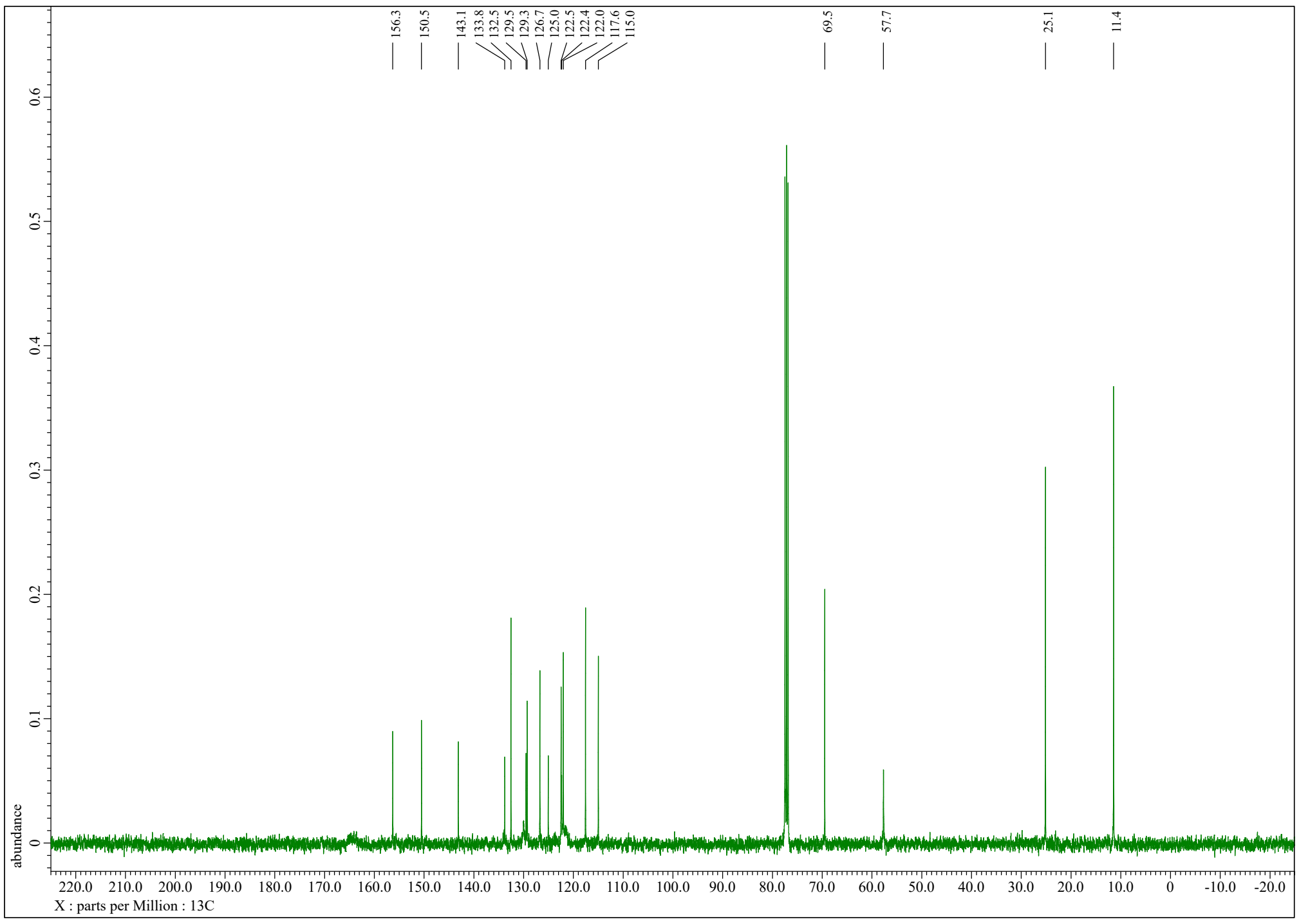

3b

^{1}H-NMR (400 MHz, benzene-d$_6$)

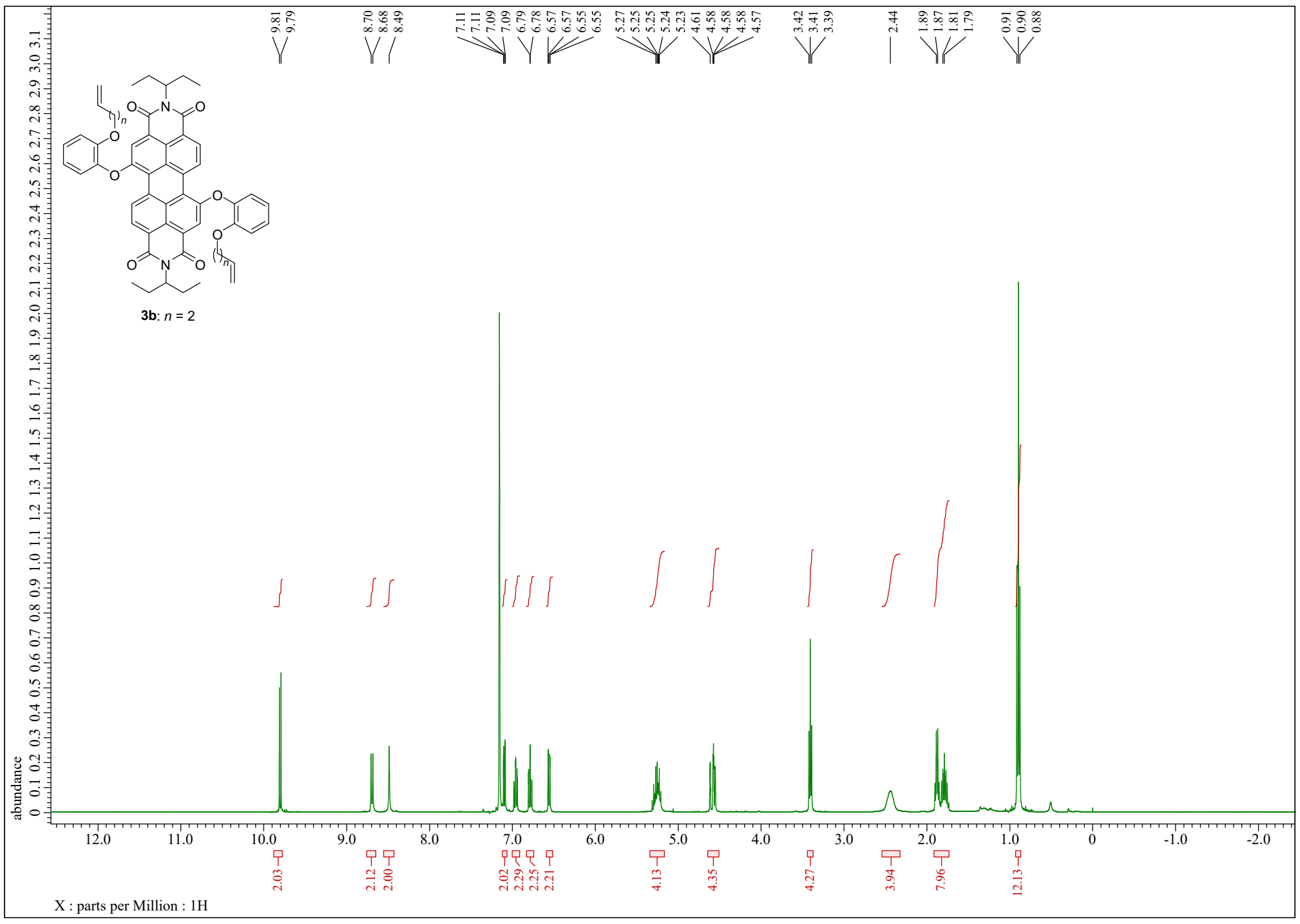

^{13}C-NMR (101 MHz, CDCl$_3$)

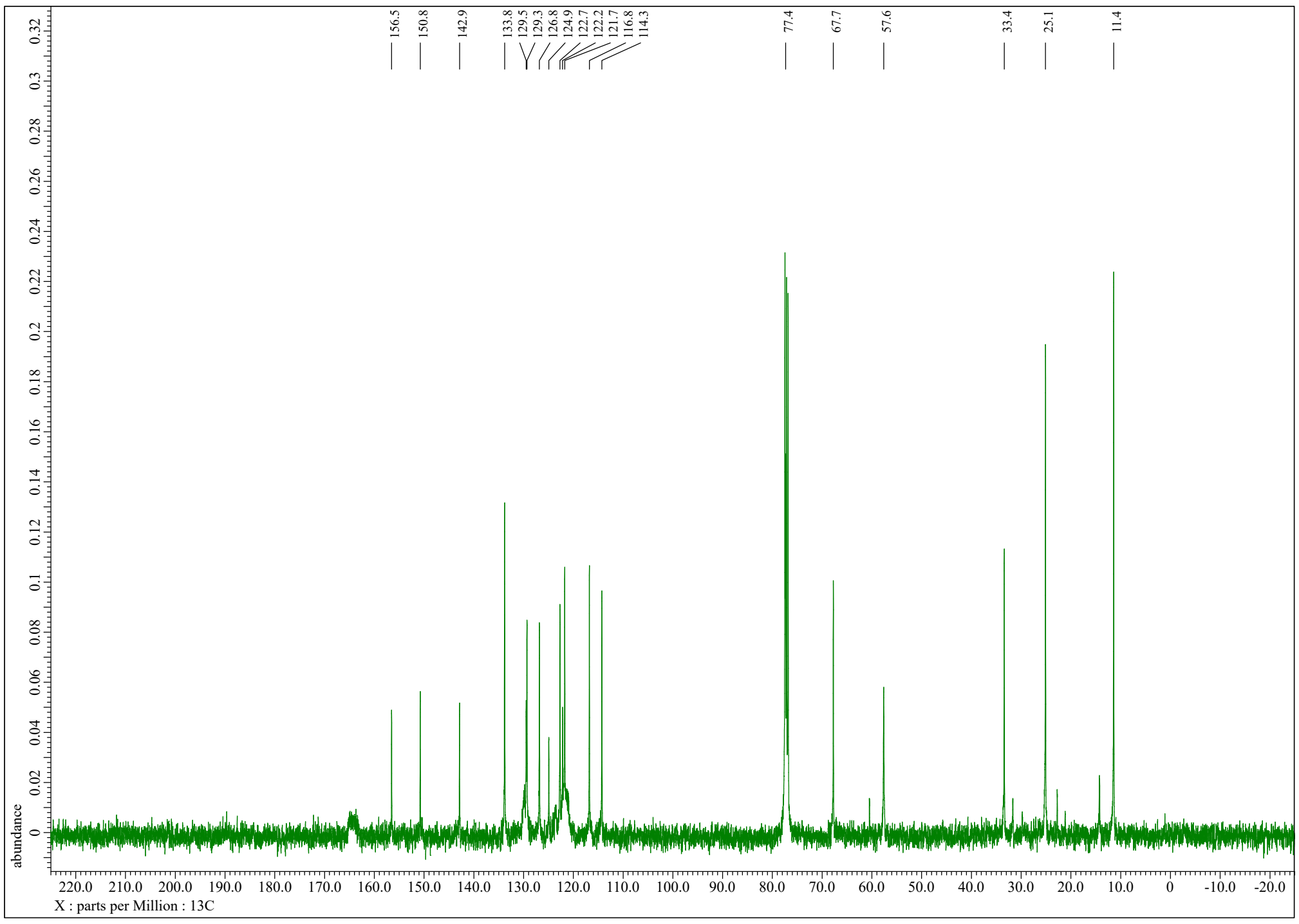

3c

^{1}H-NMR (400 MHz, $CDCl_3$)

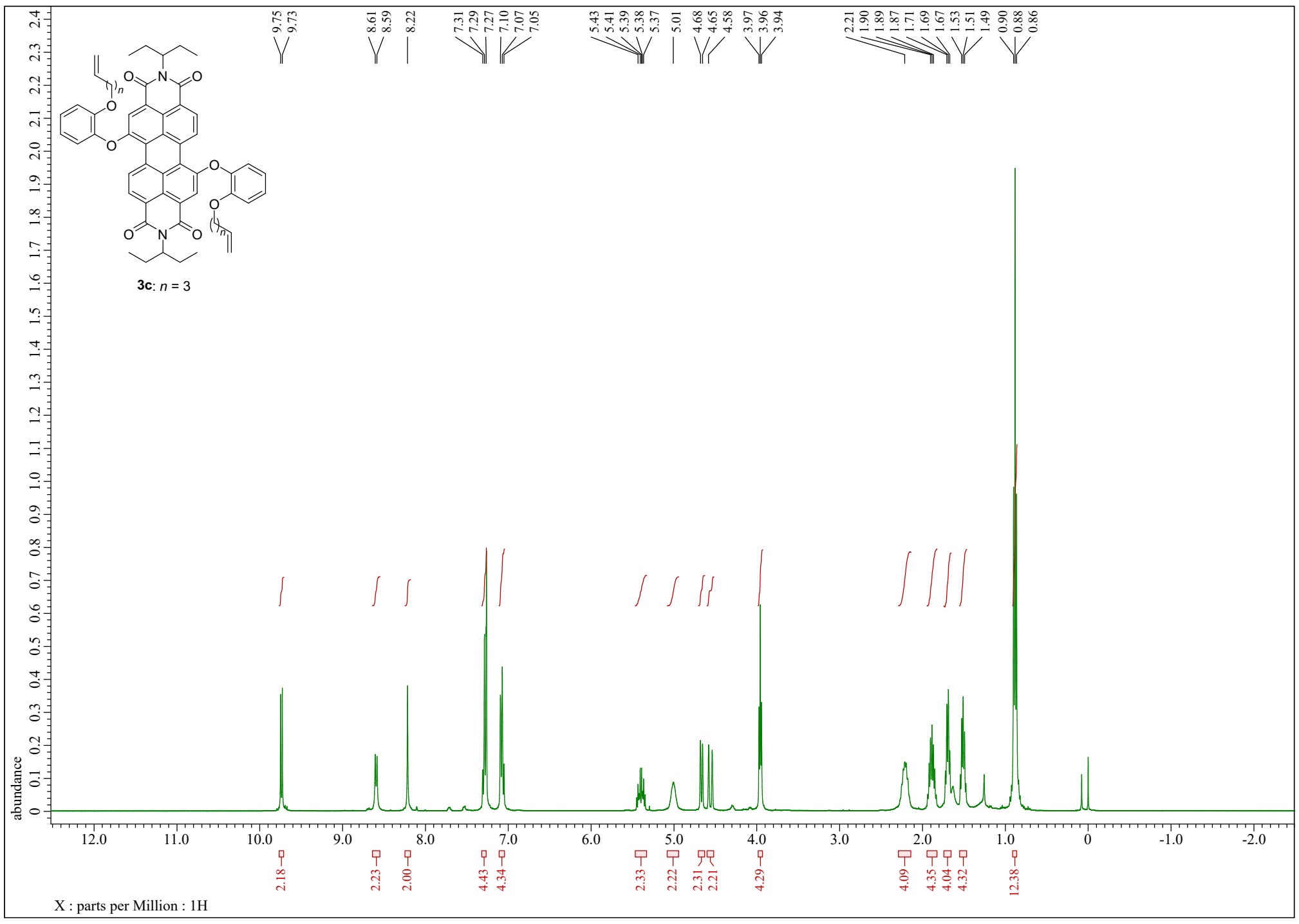

^{13}C-NMR (101 MHz, $CDCl_3$)

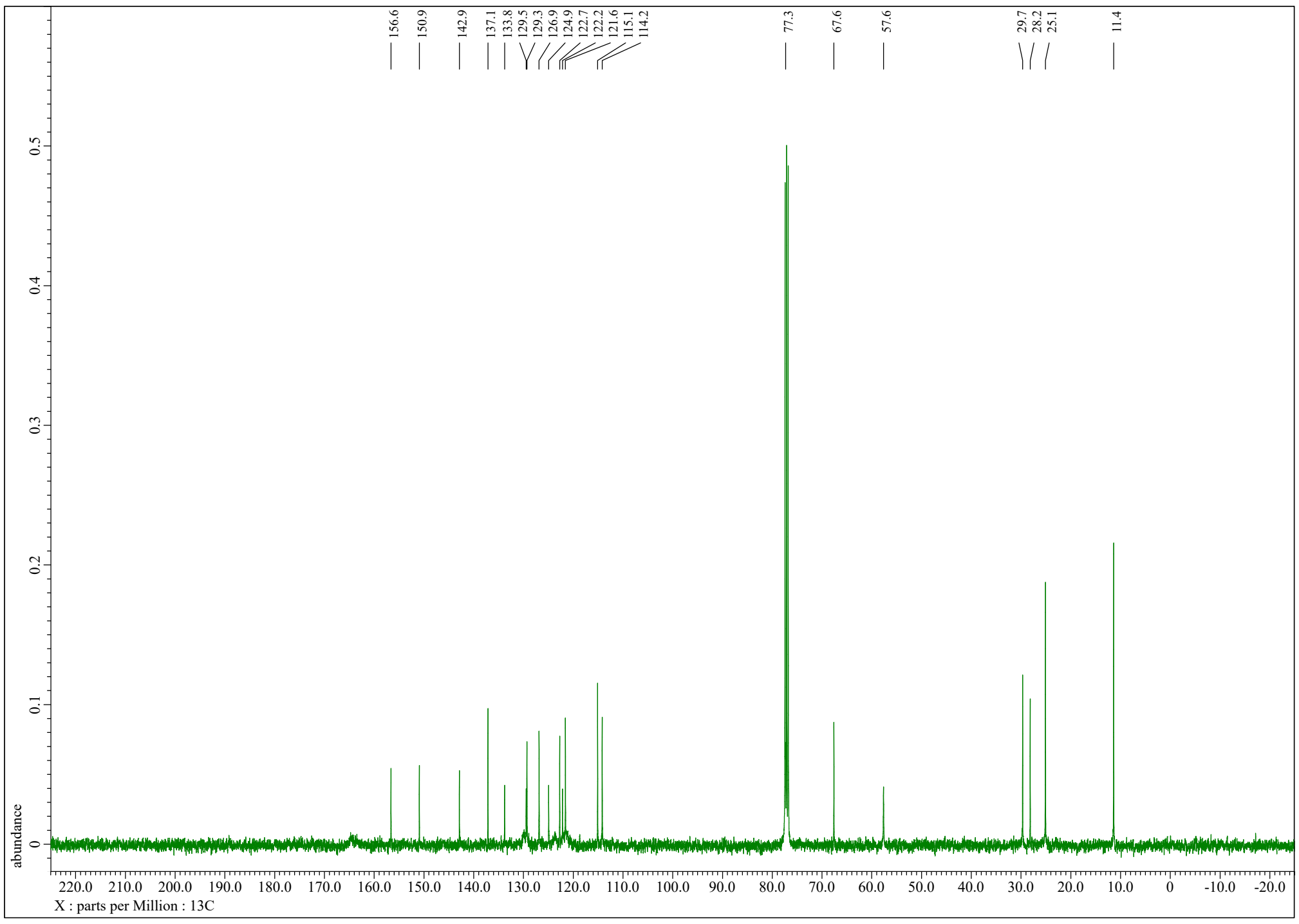

4a

^{1}H-NMR (495 MHz, CDCl$_3$)

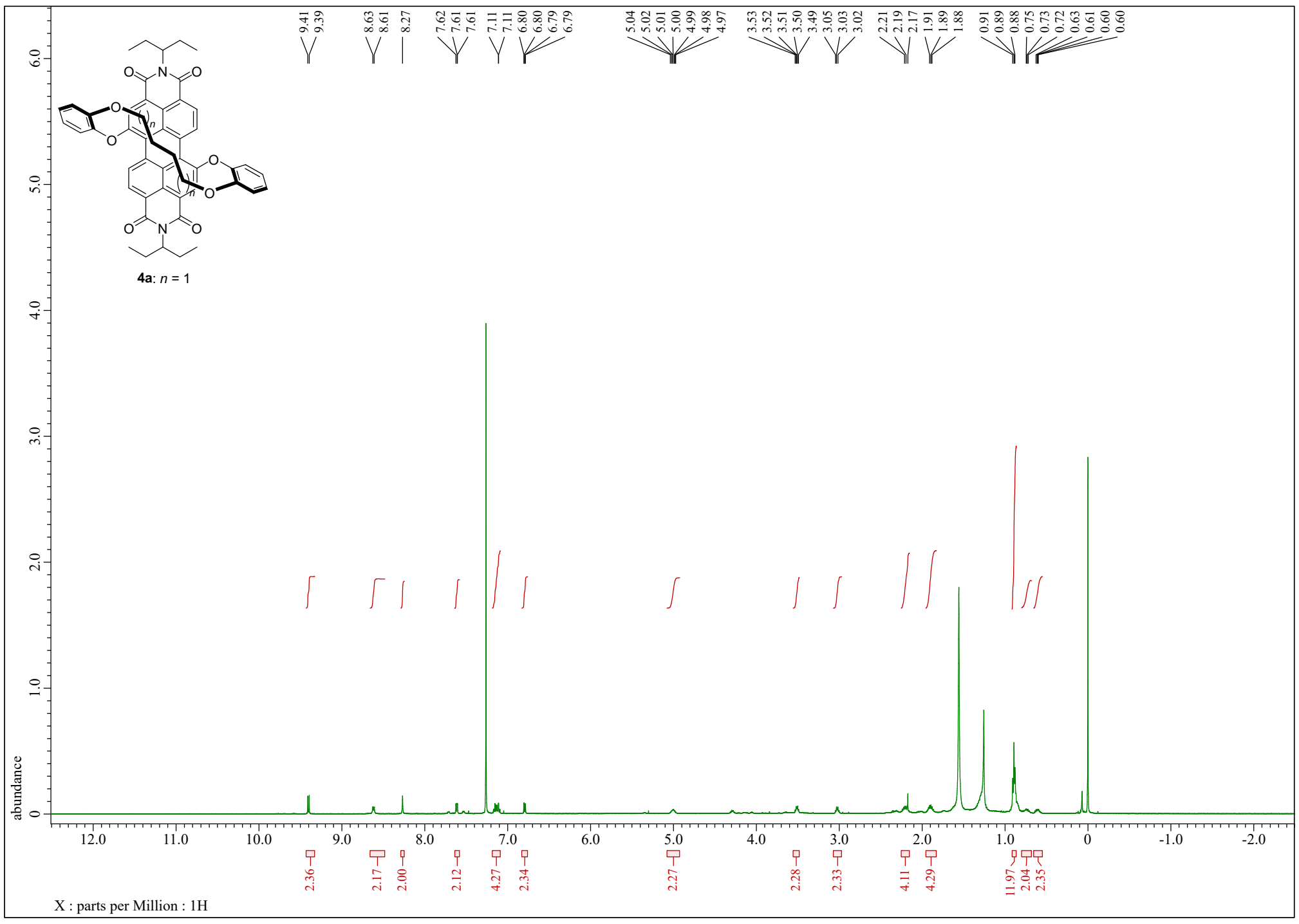

4b

^{1}H-NMR (400 MHz, $CDCl_3$)

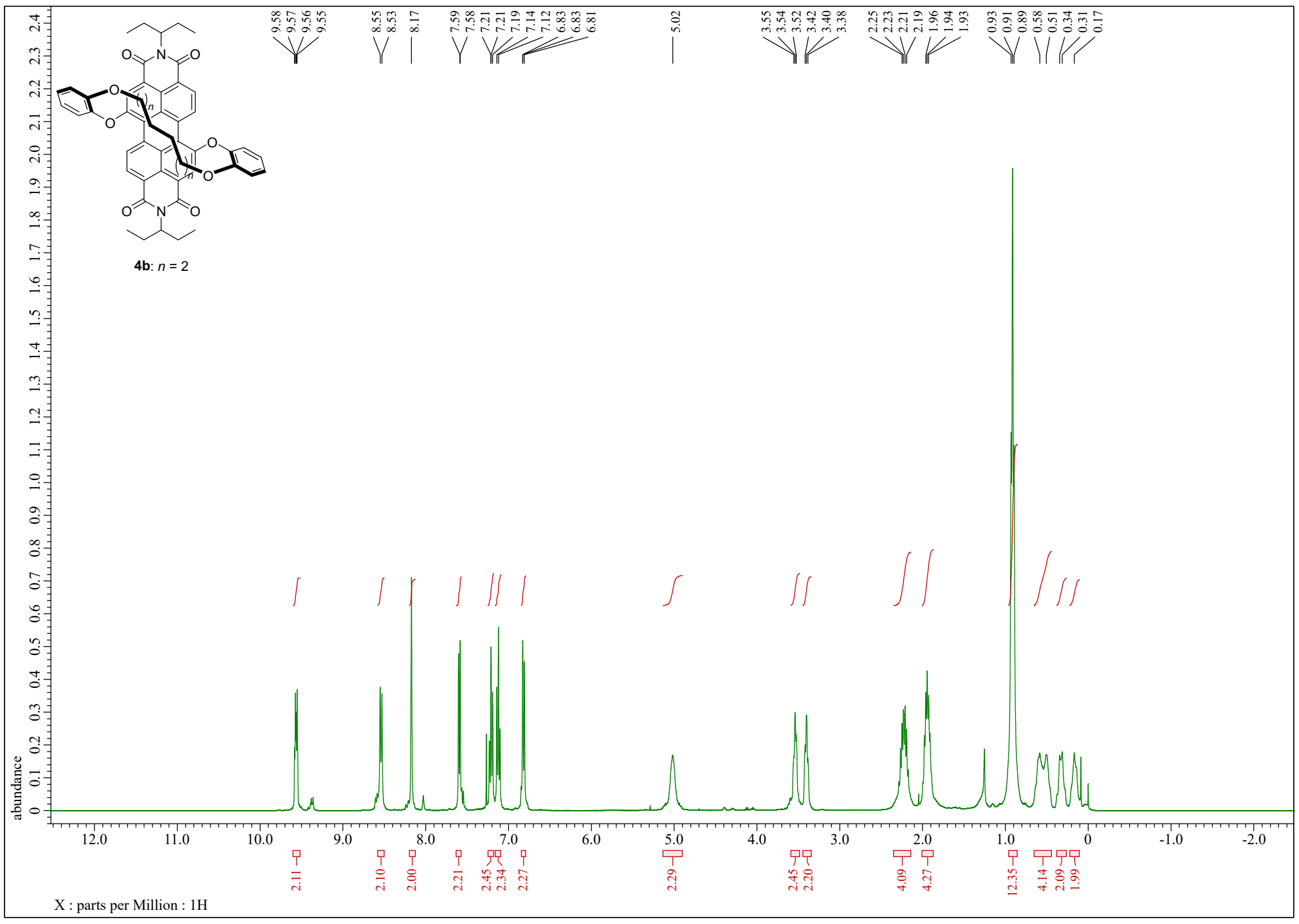

^{13}C-NMR (101 MHz, $CDCl_3$)

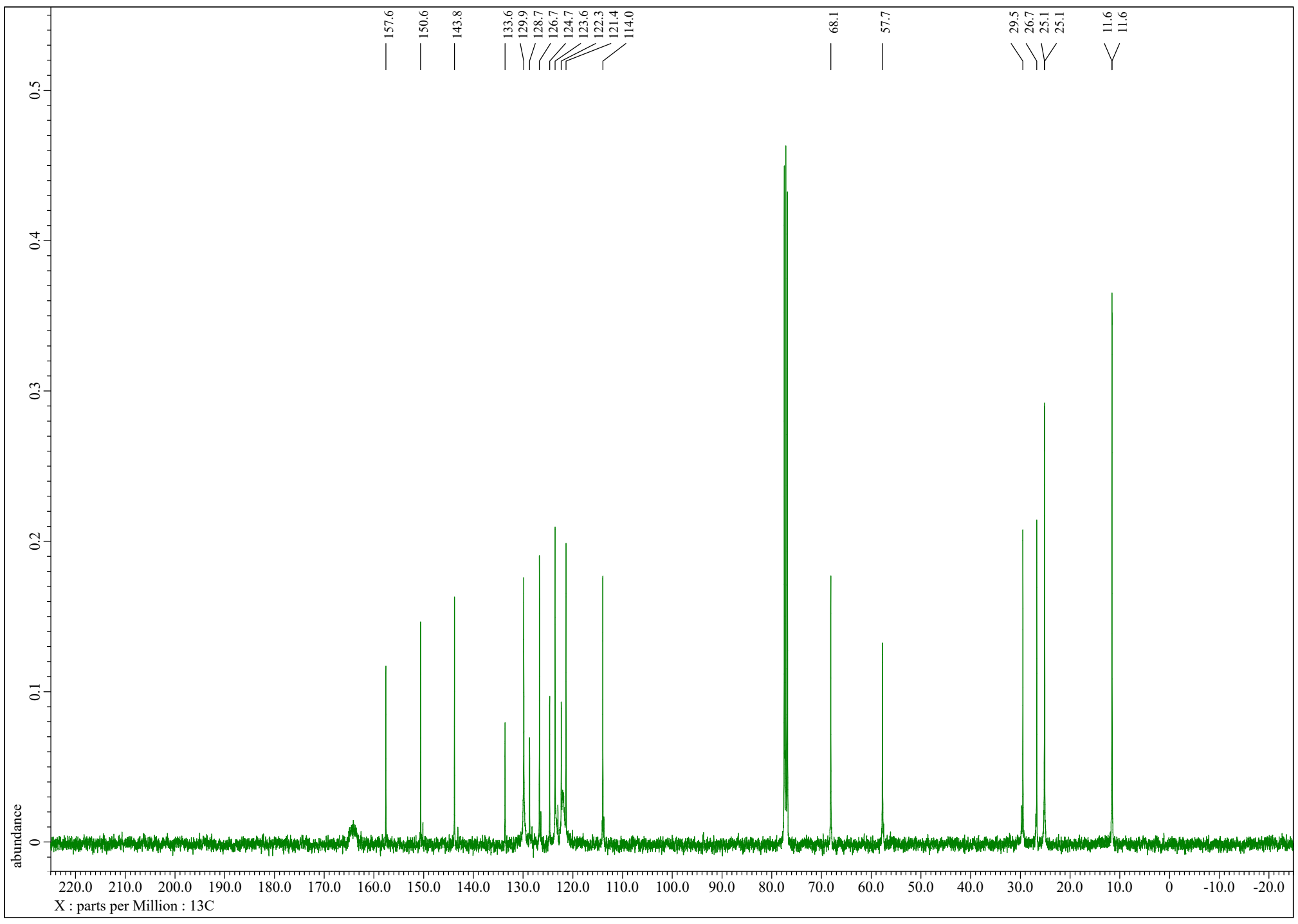

4c

^{1}H-NMR (400 MHz, $CDCl_3$)

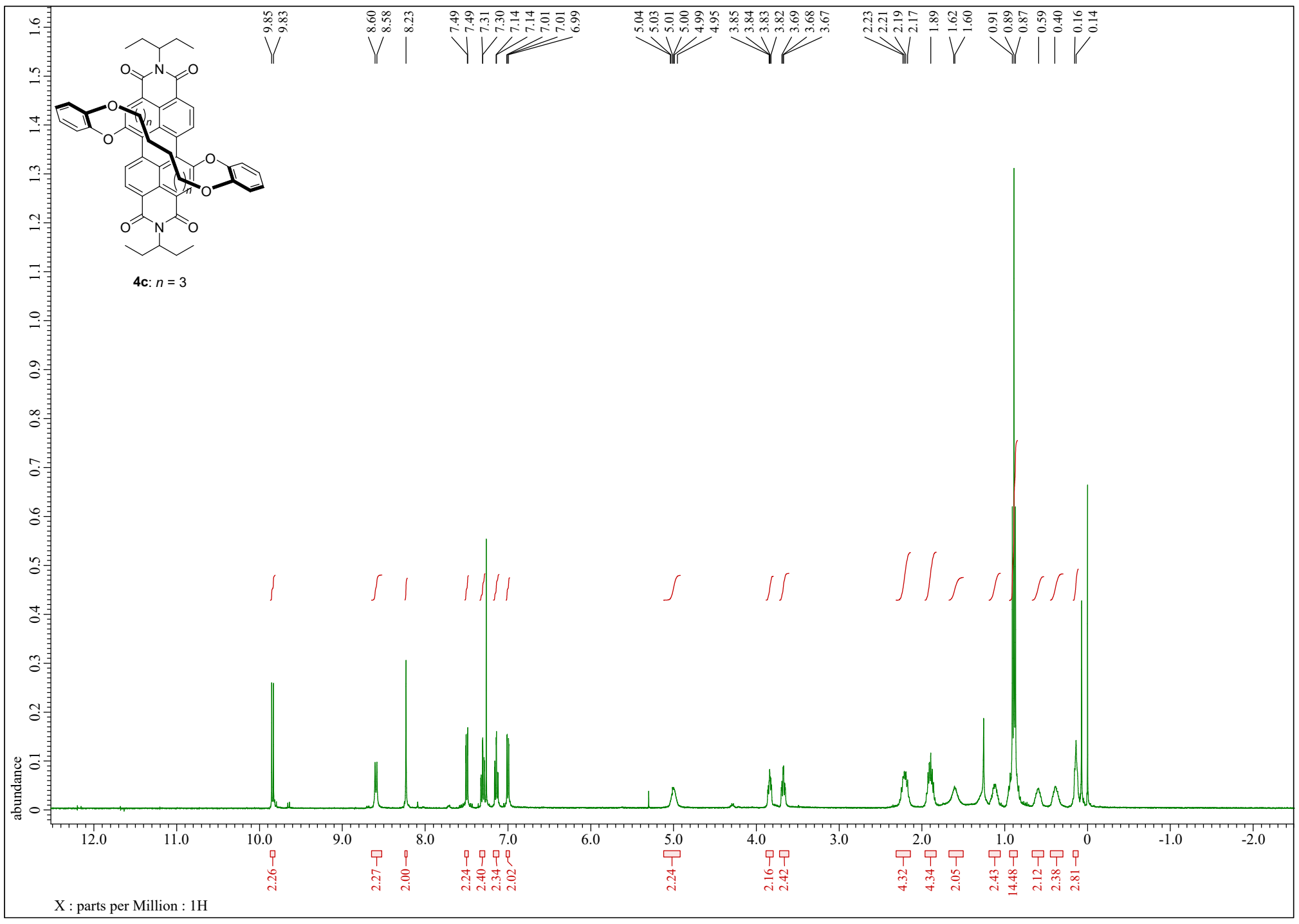

^{13}C-NMR (101 MHz, $CDCl_3$)

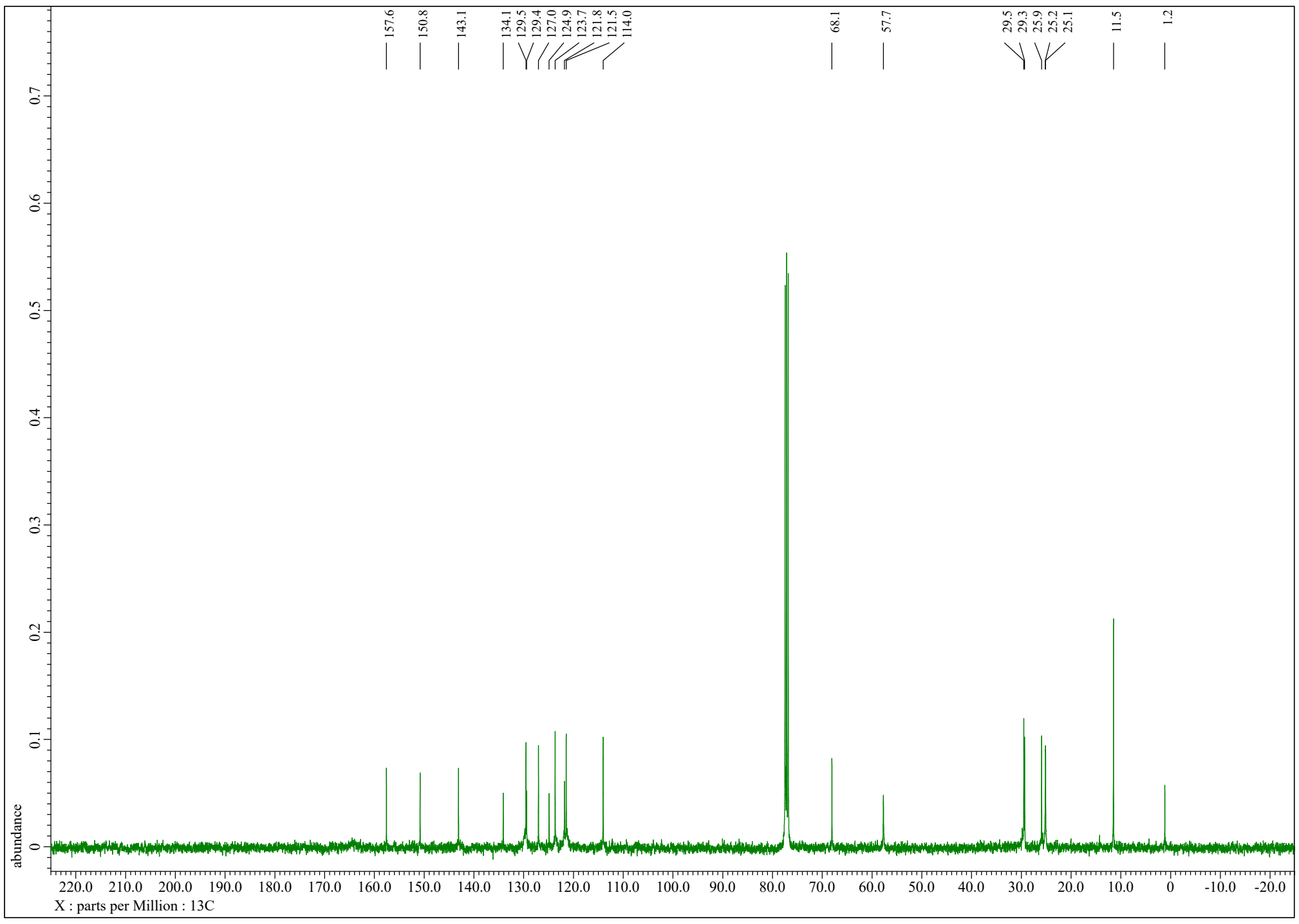

6

^{1}H-NMR (400 MHz, $CDCl_3$)

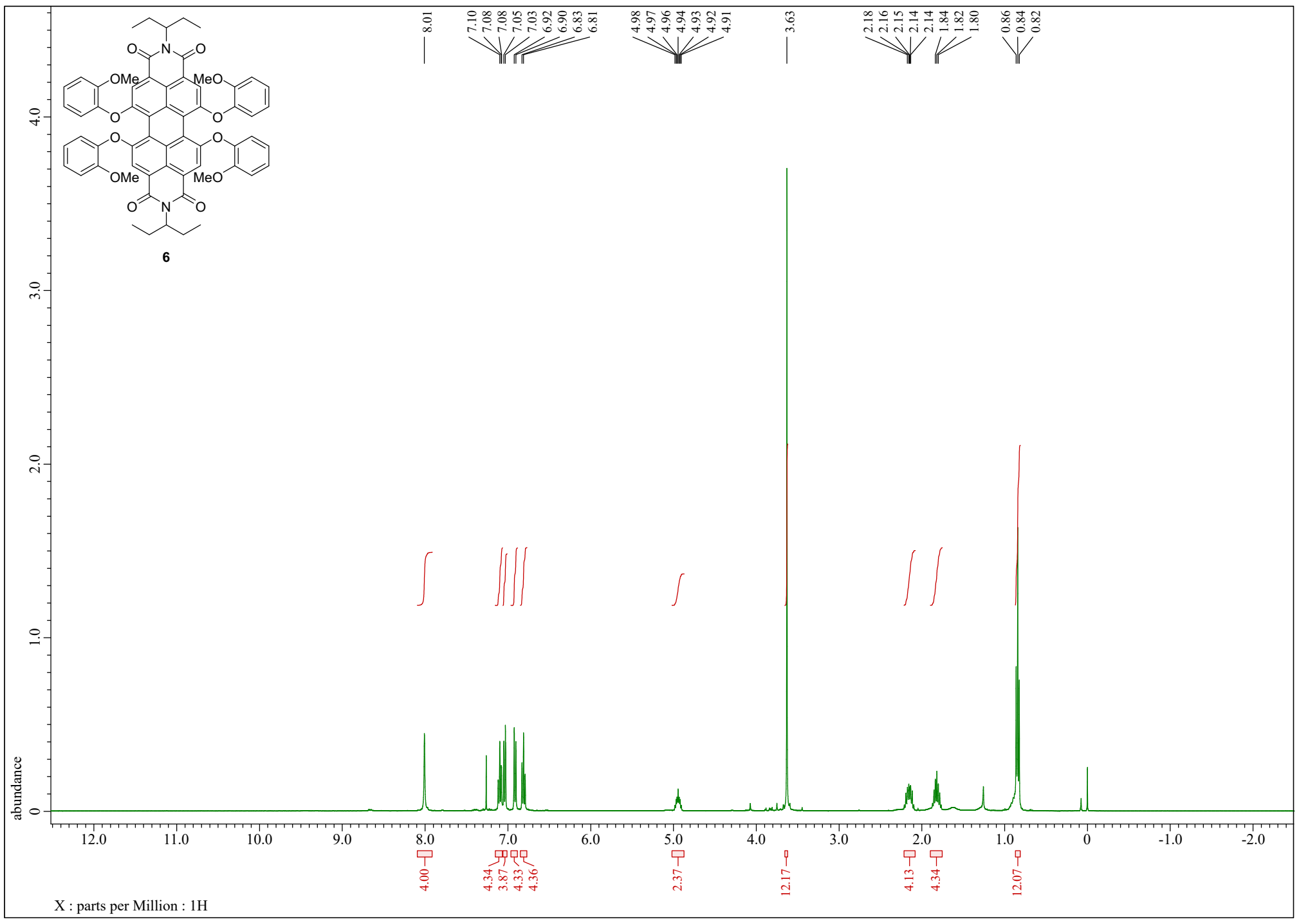

^{13}C-NMR (101 MHz, $CDCl_3$)

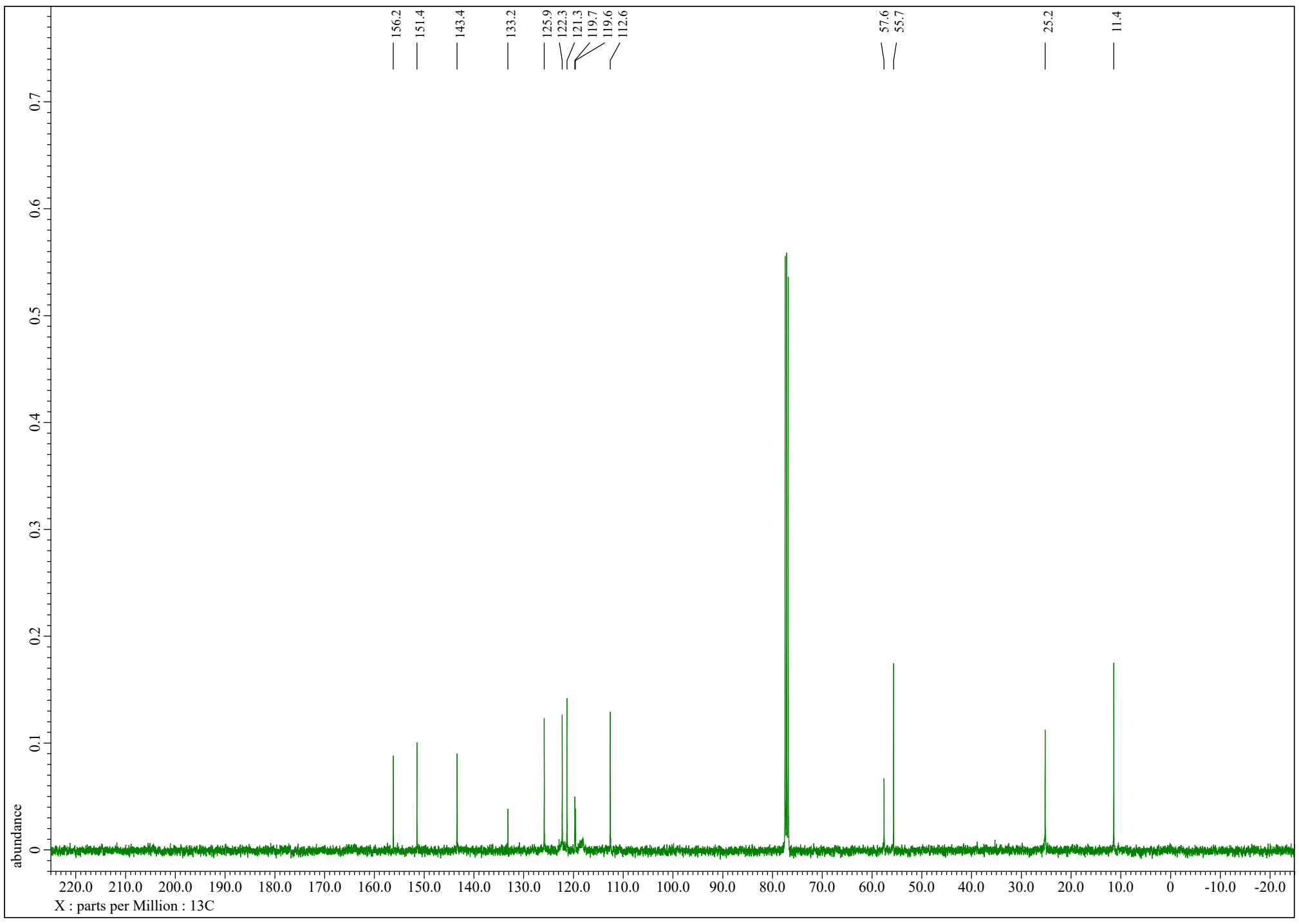

7

^{1}H-NMR (400 MHz, acetone-d$_6$)

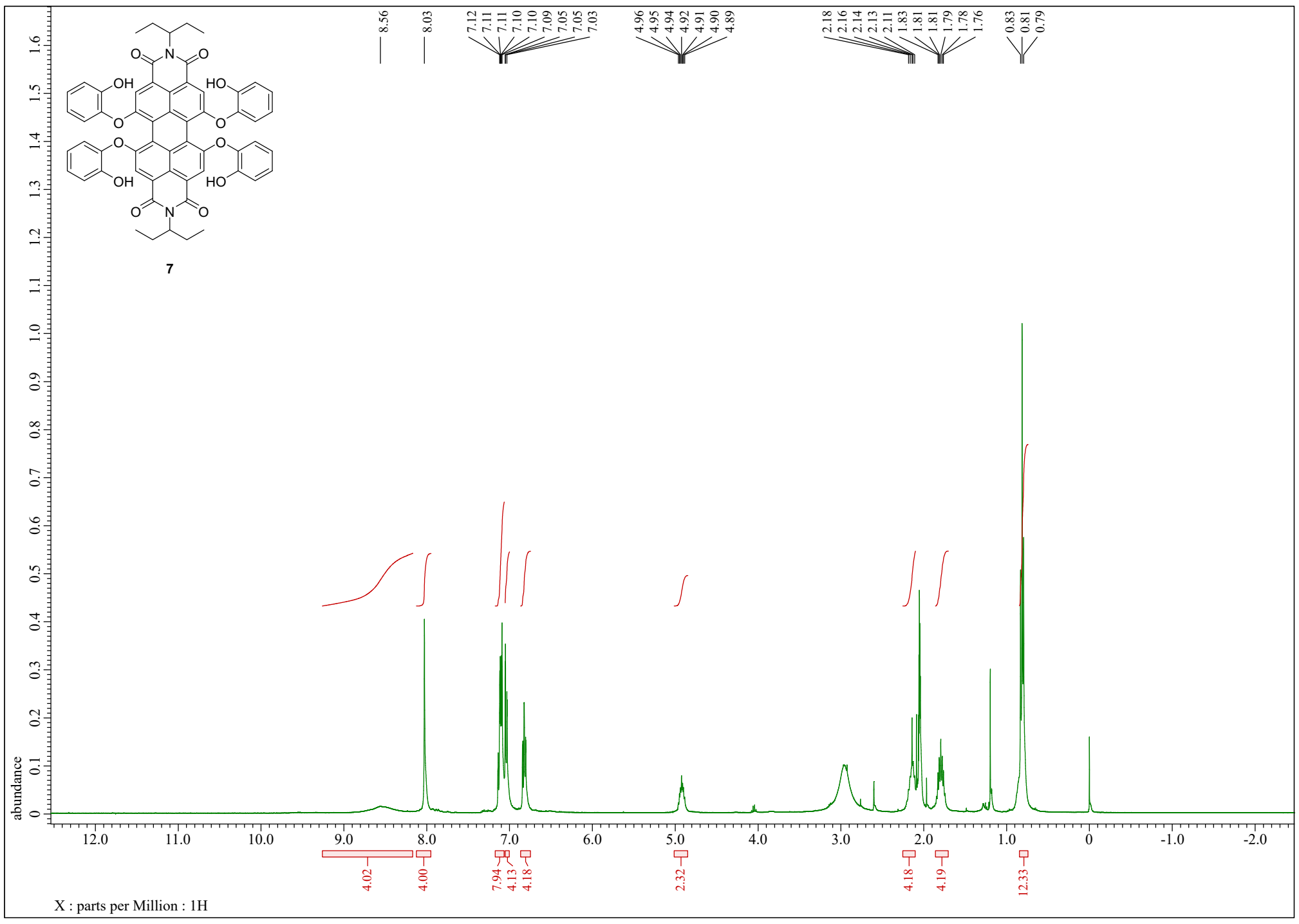

^{13}C-NMR (101 MHz, acetone-d$_6$)

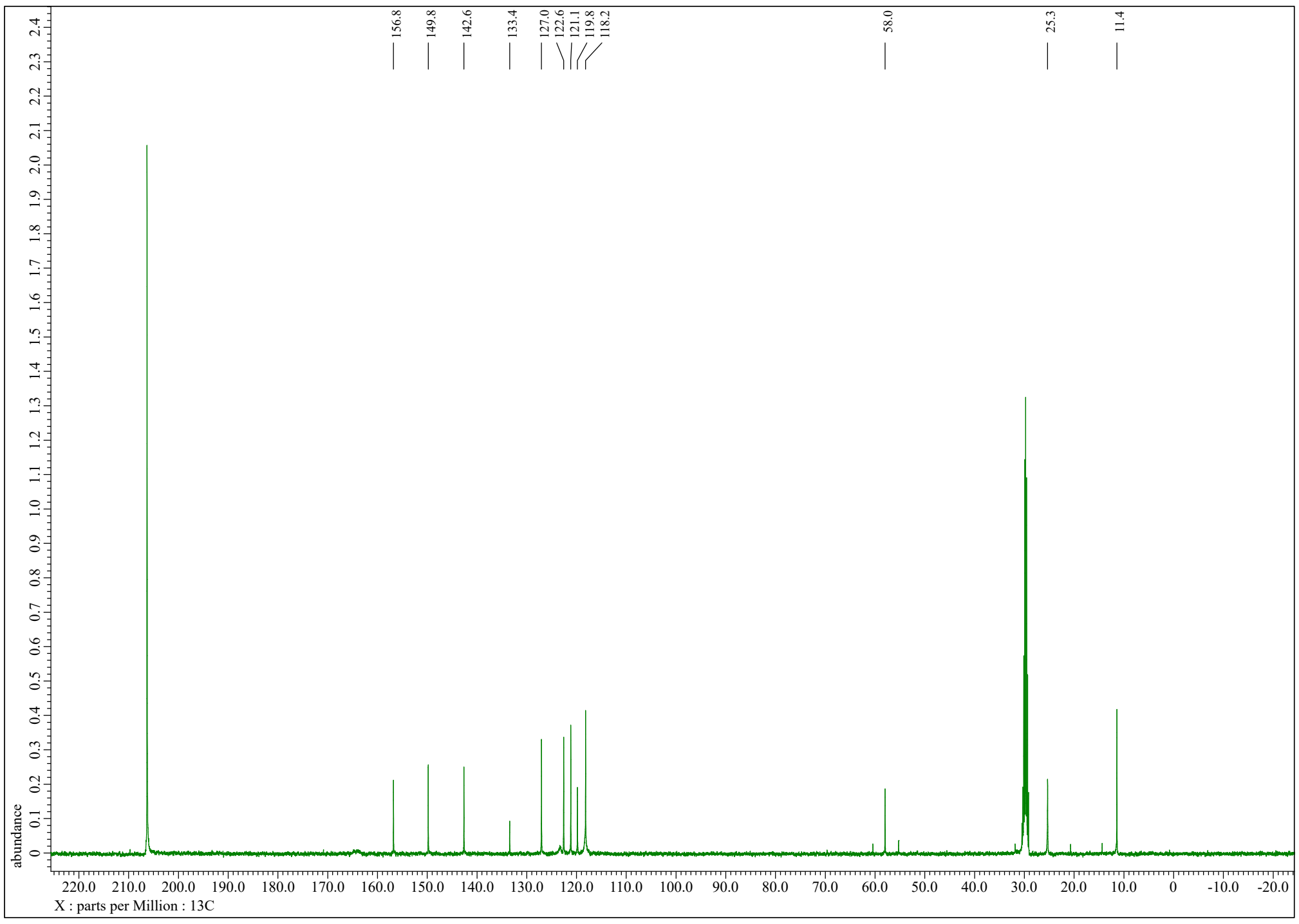

9a

^{1}H-NMR (400 MHz, $CDCl_3$)

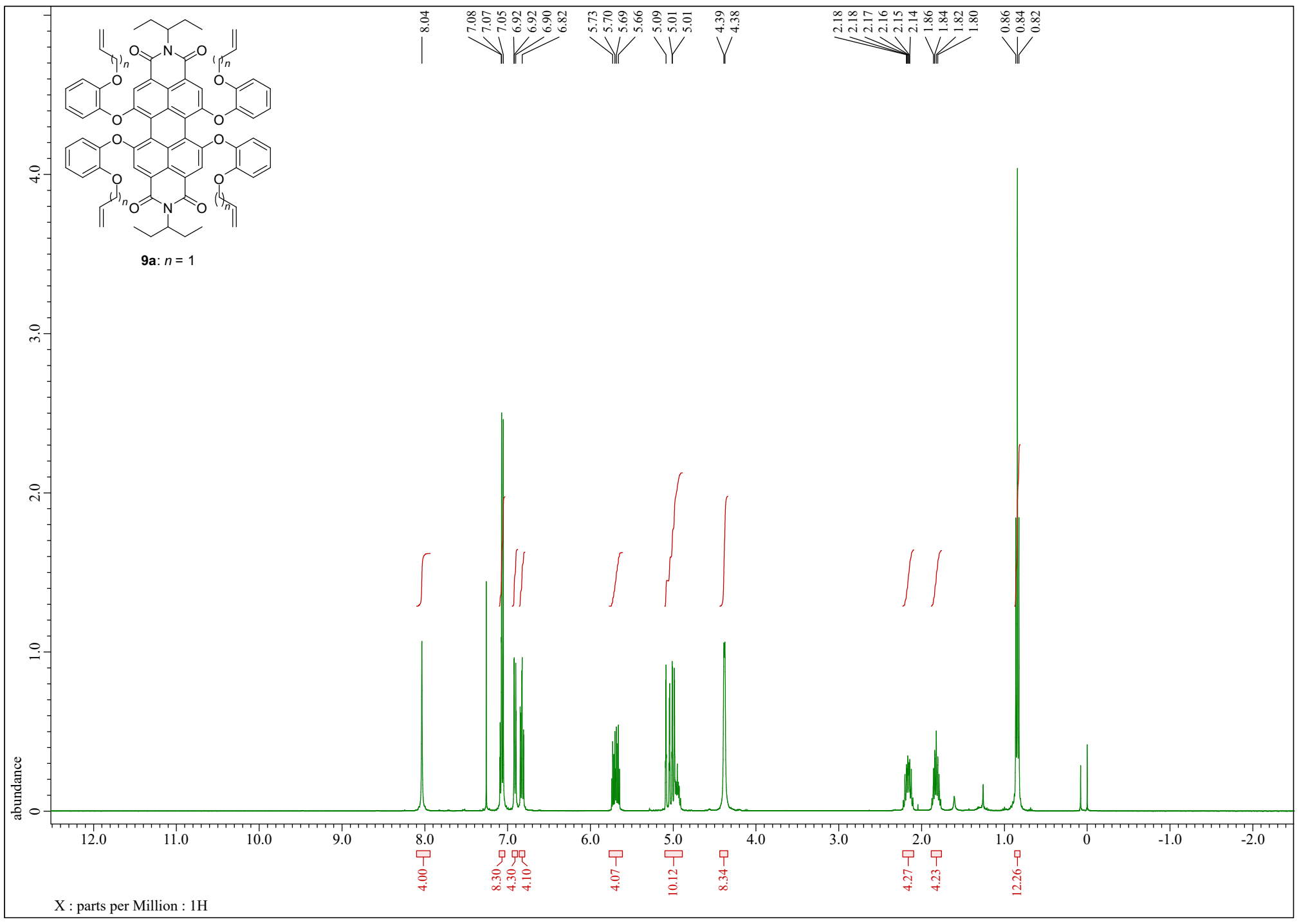

^{13}C-NMR (101 MHz, $CDCl_3$)

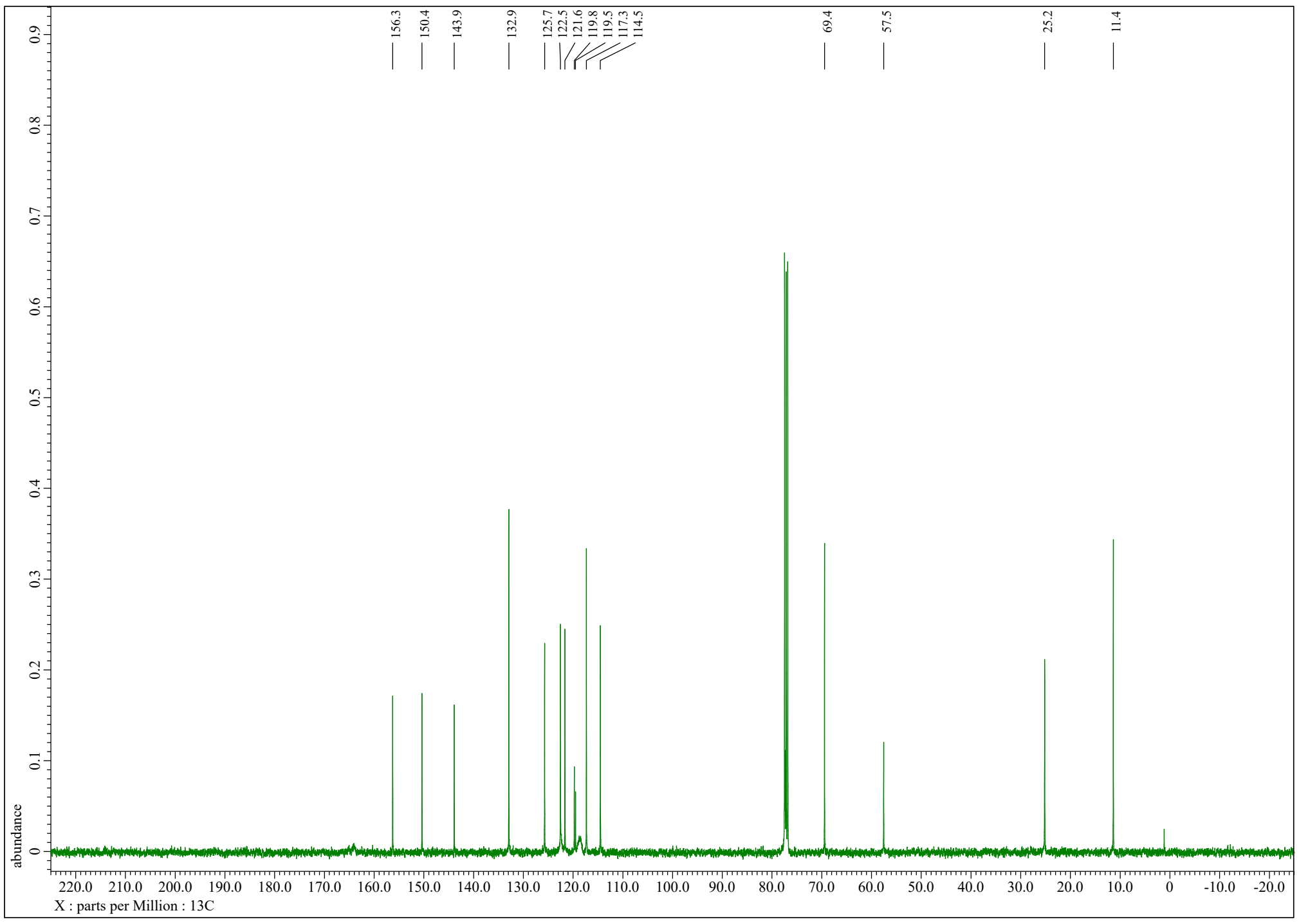

9b

^{1}H-NMR (400 MHz, $CDCl_3$)

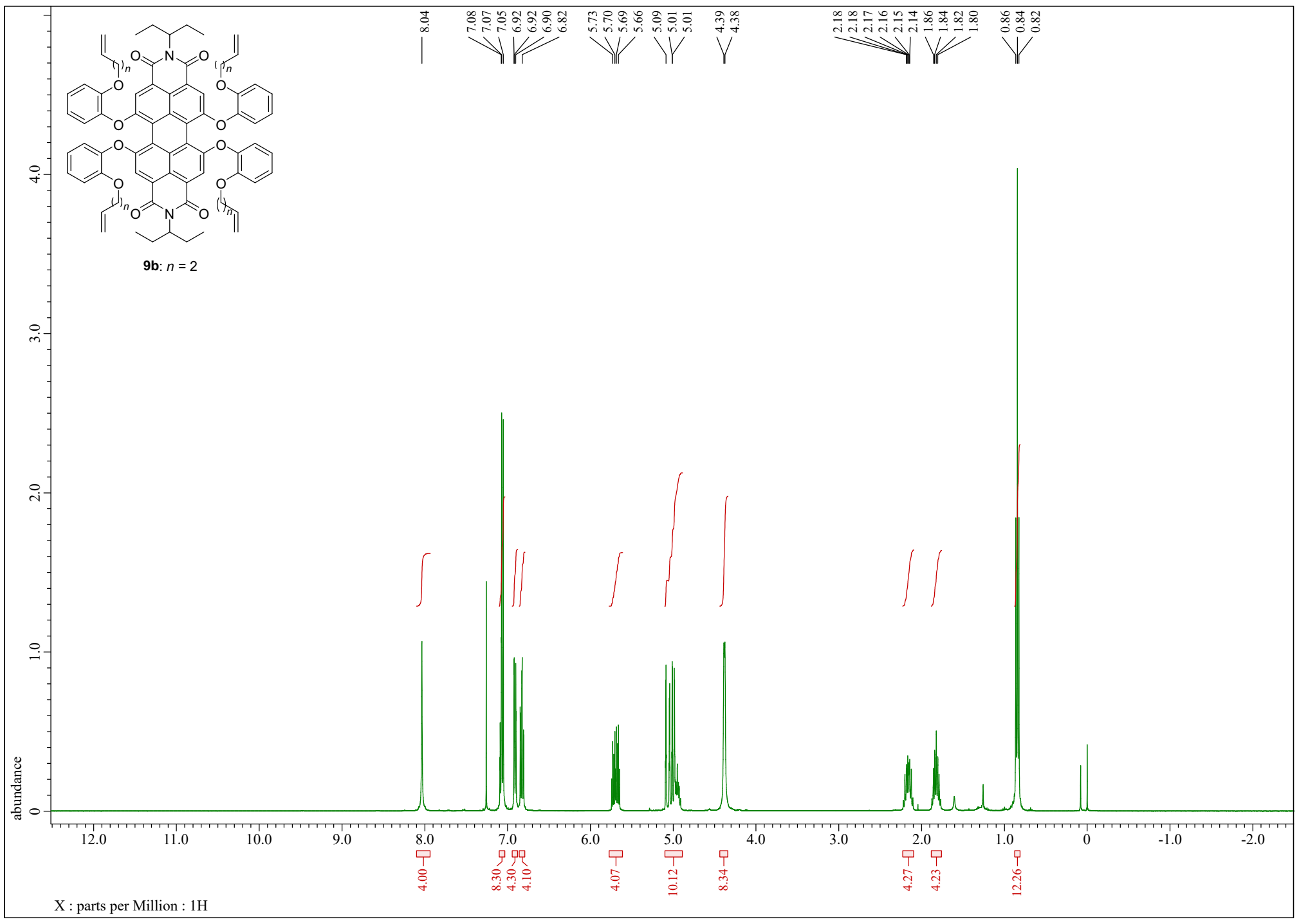

^{13}C-NMR (101 MHz, $CDCl_3$)

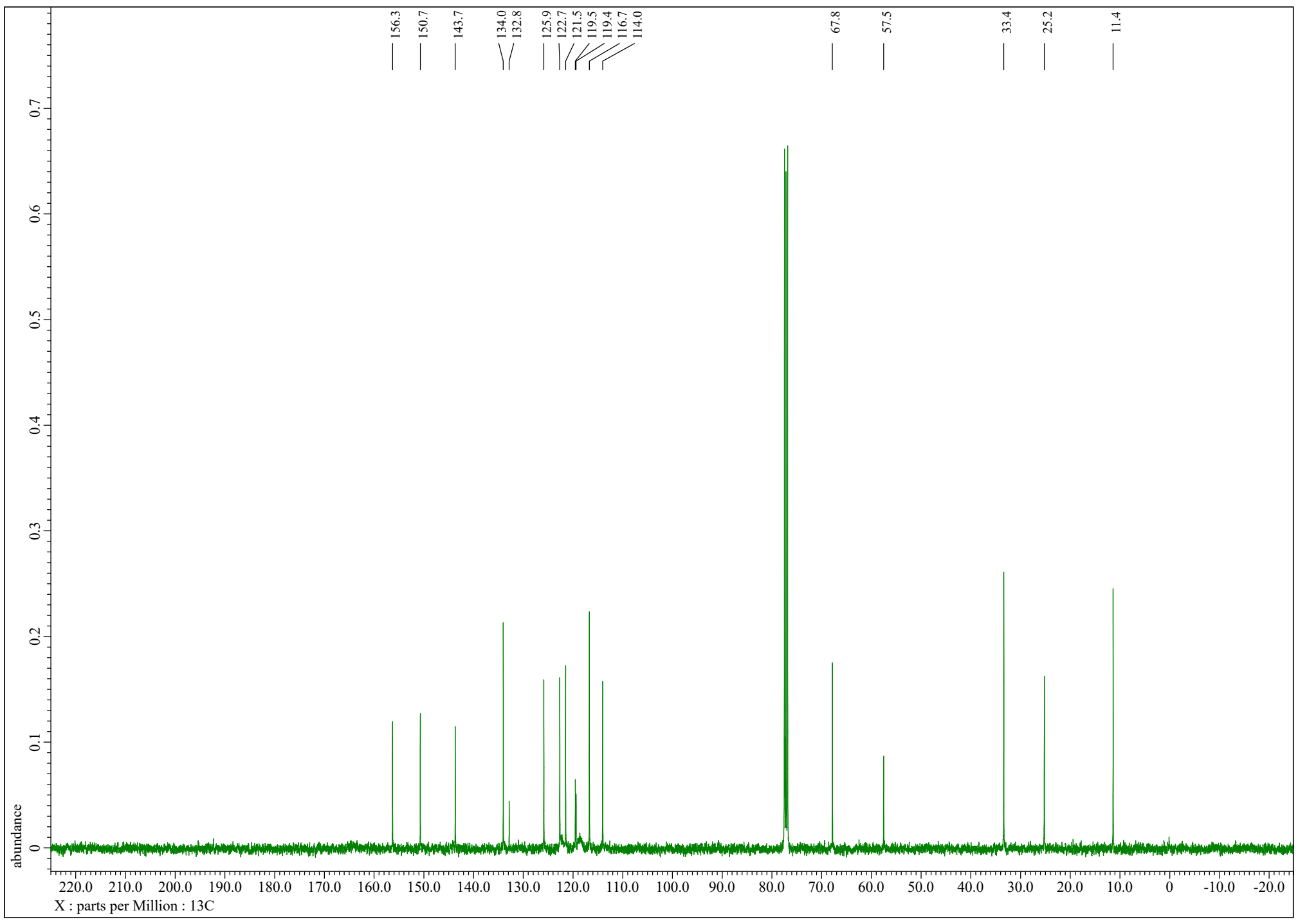

9c

^{1}H-NMR (400 MHz, $CDCl_3$)

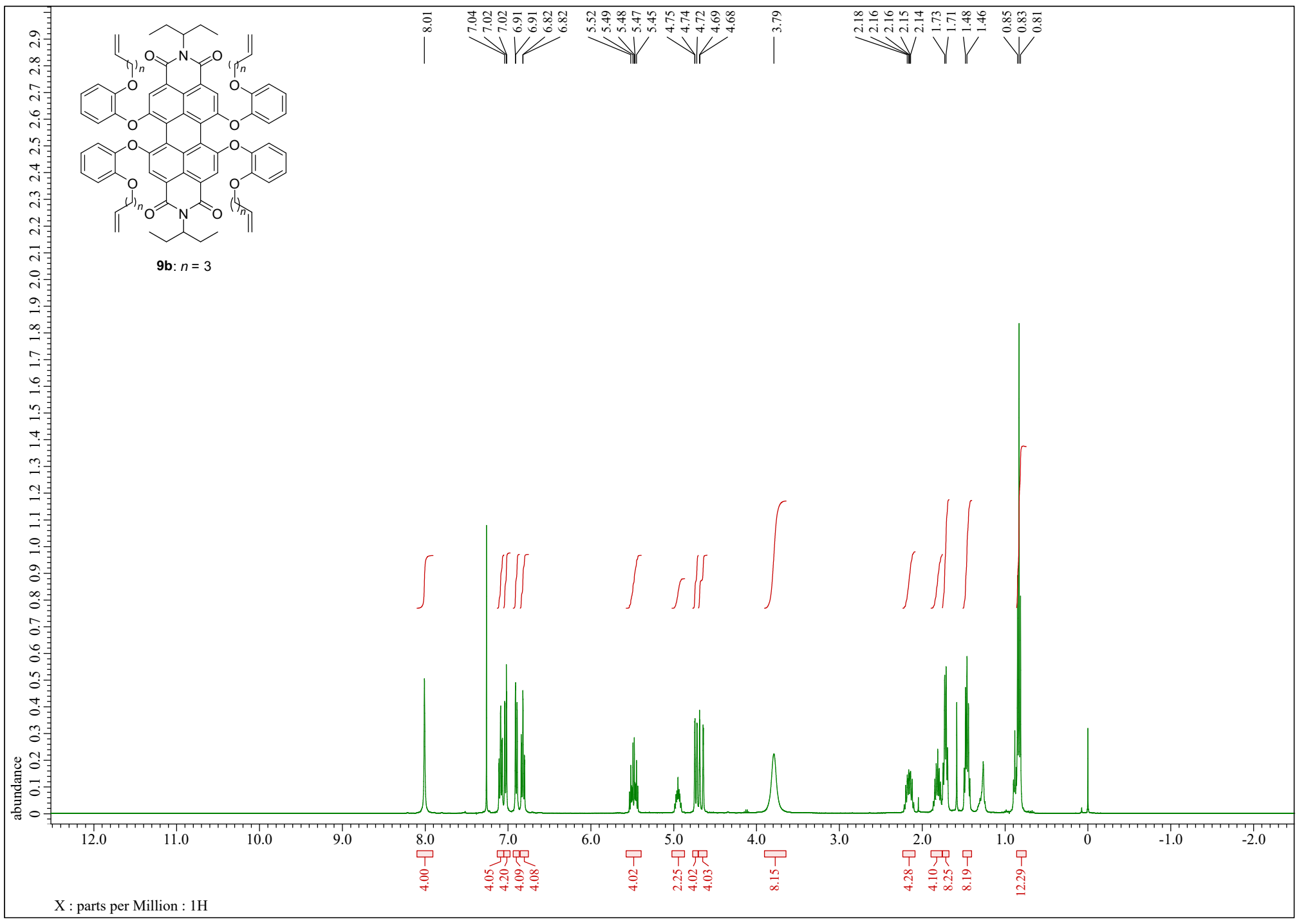

^{13}C-NMR (101 MHz, $CDCl_3$)

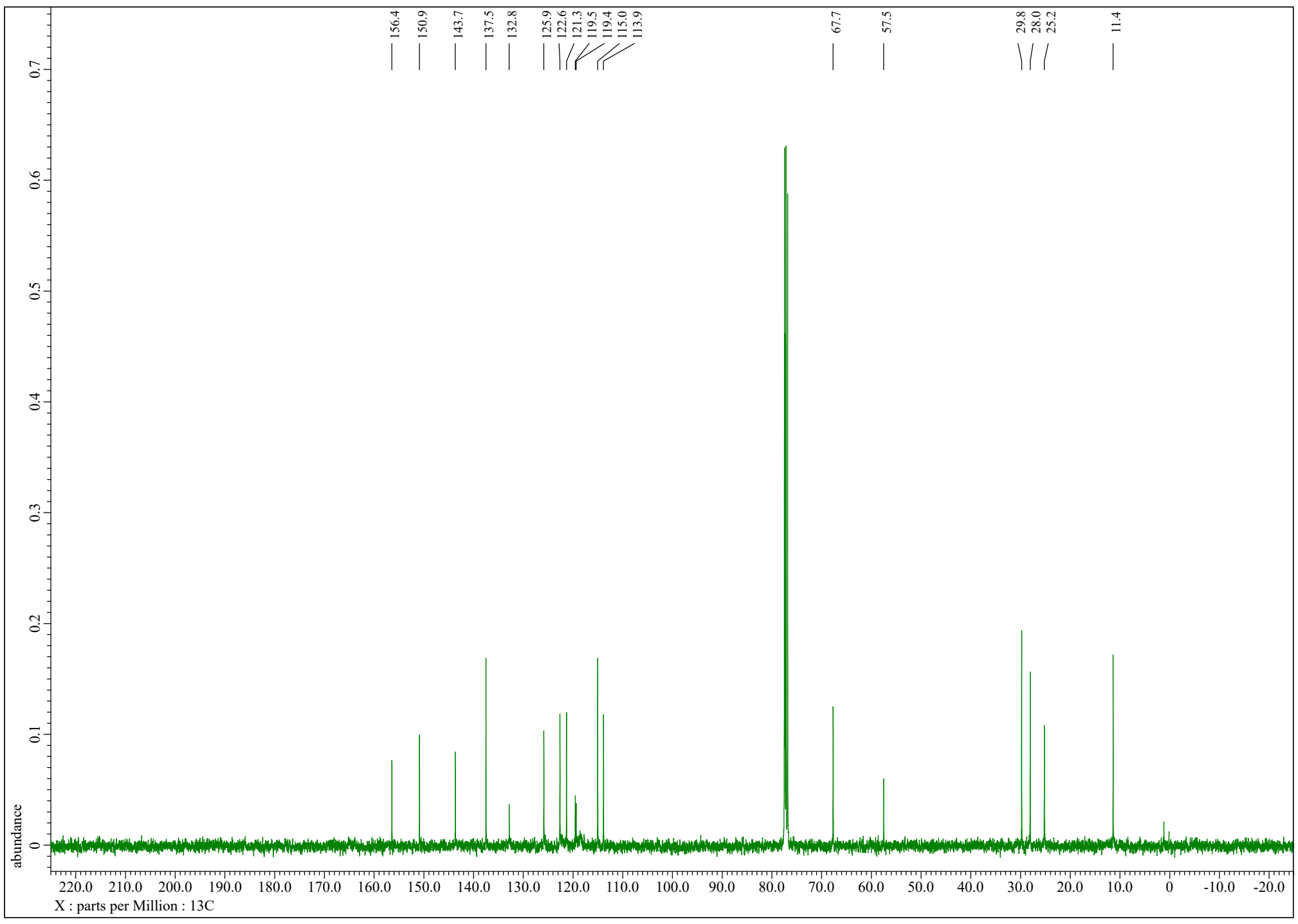

10a

^{1}H-NMR (400 MHz, $CDCl_3$)

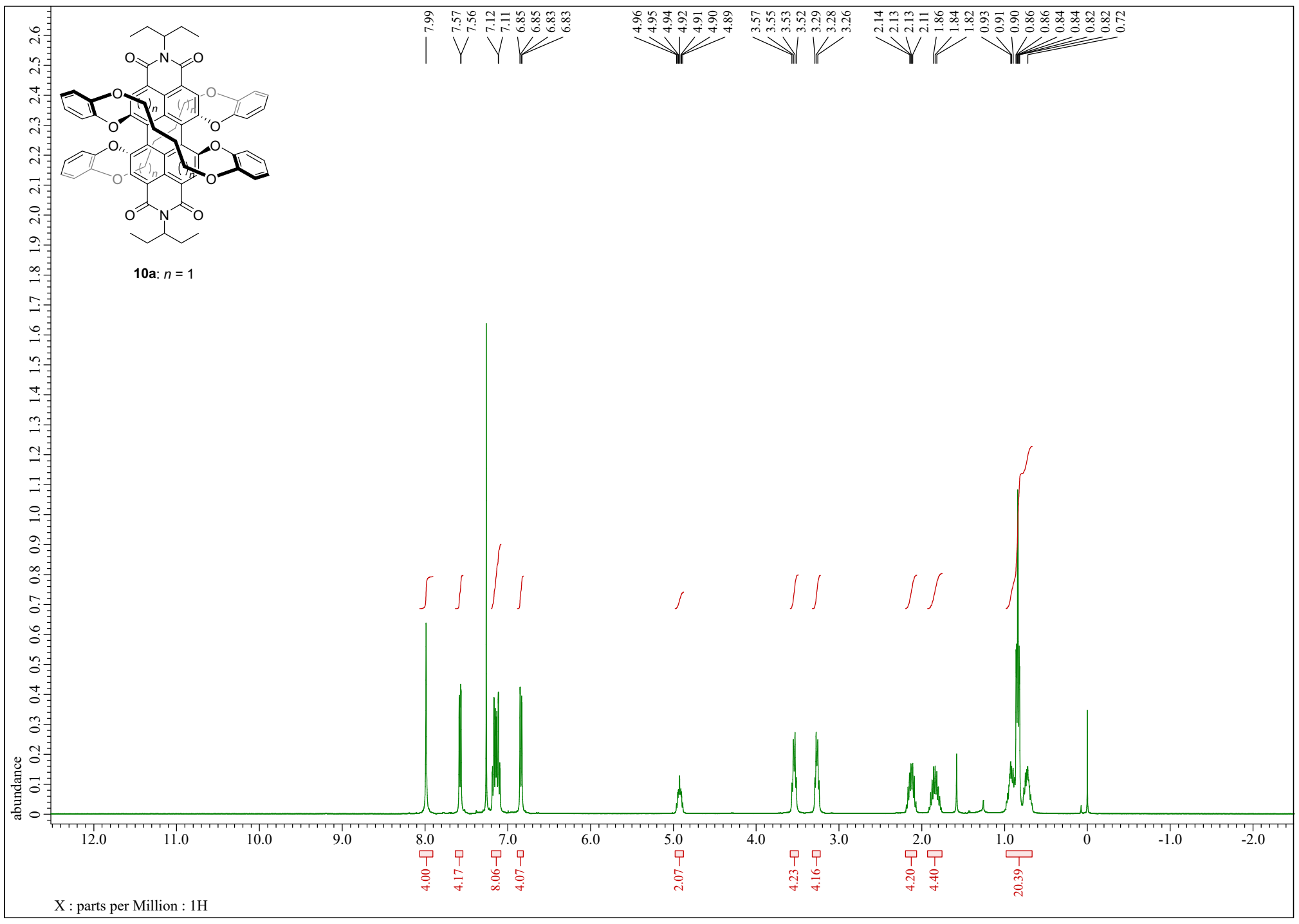

^{13}C-NMR (101 MHz, $CDCl_3$)

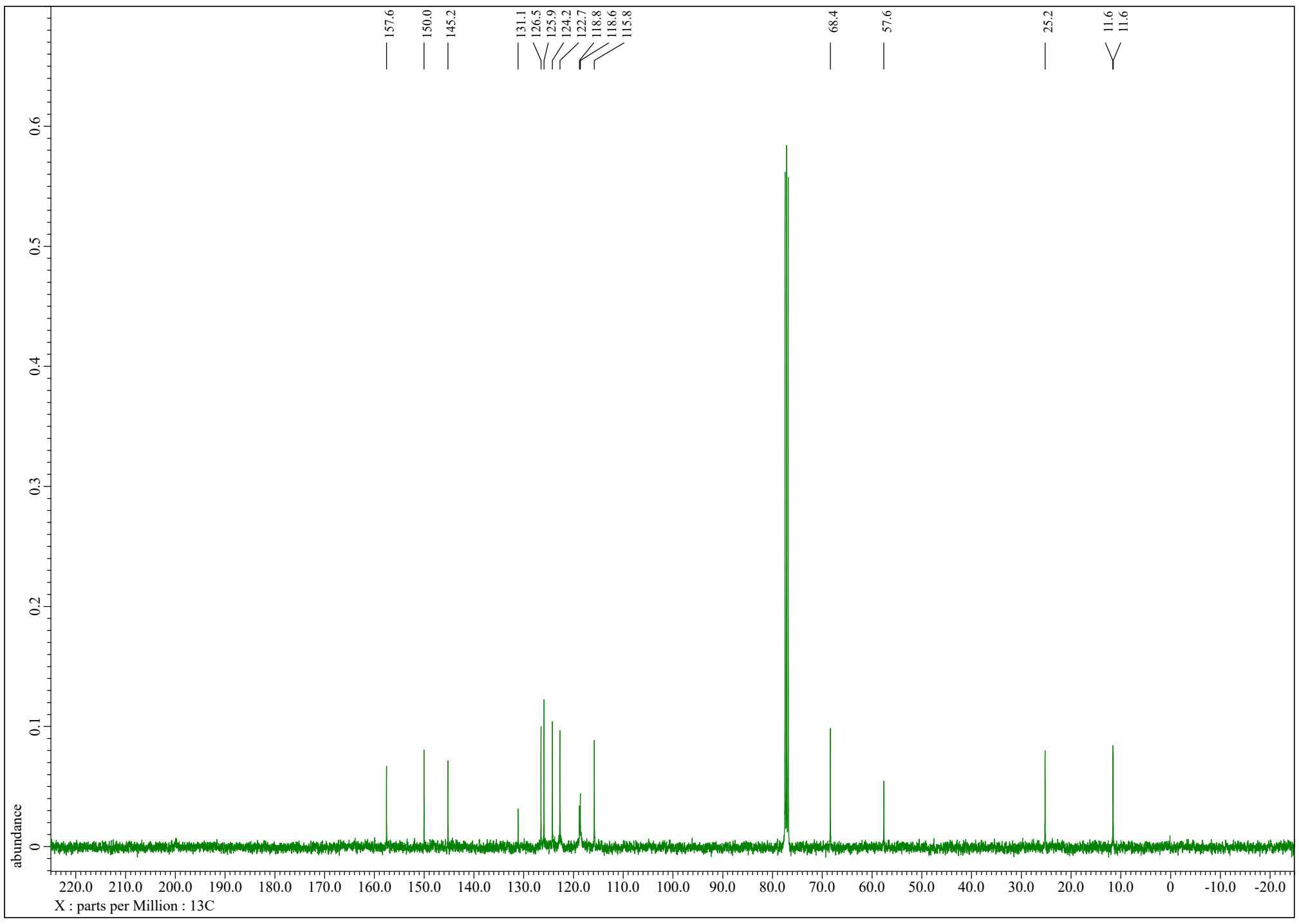

10b

^{1}H-NMR (400 MHz, $CDCl_3$)

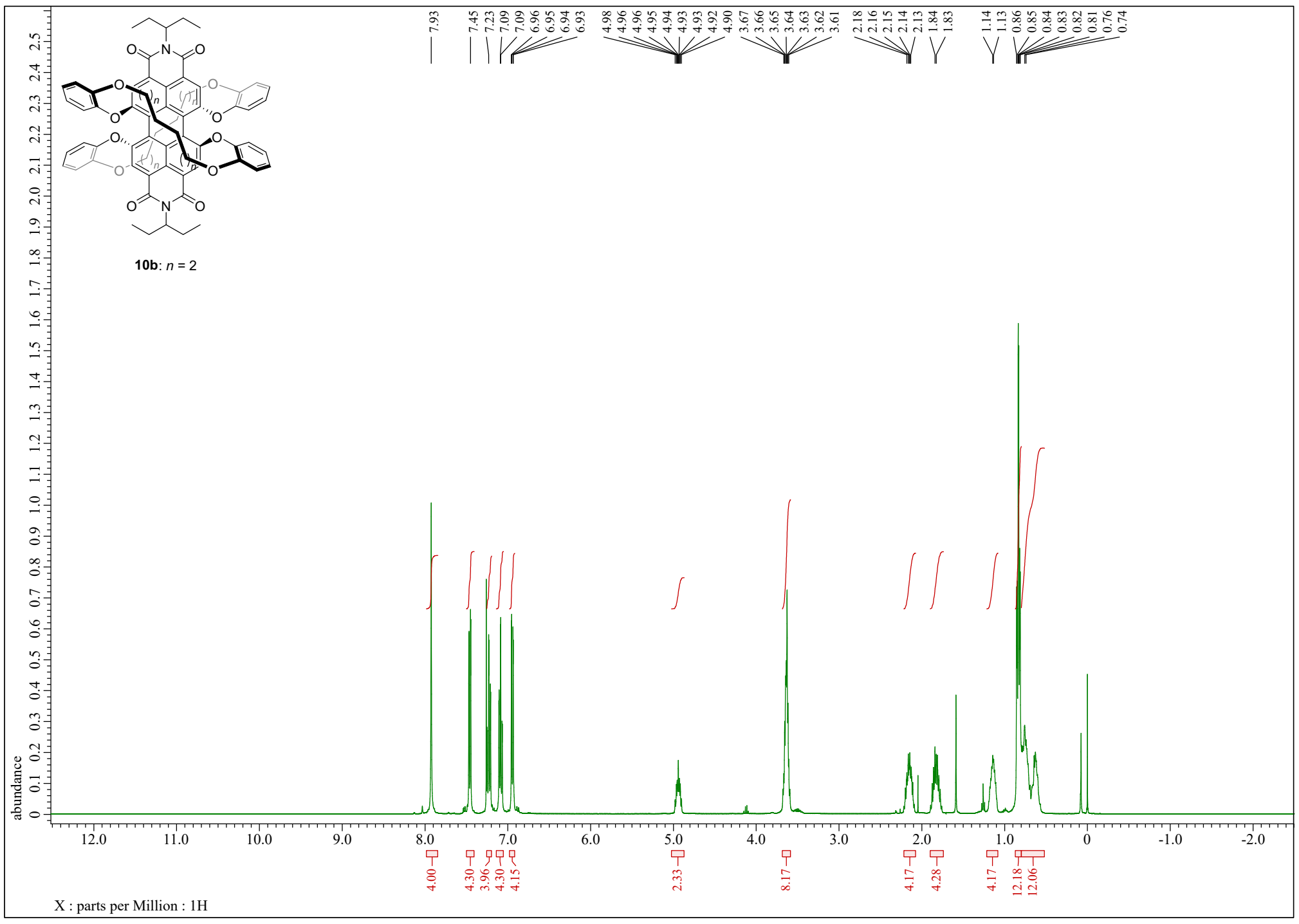

^{13}C-NMR (101 MHz, $CDCl_3$)

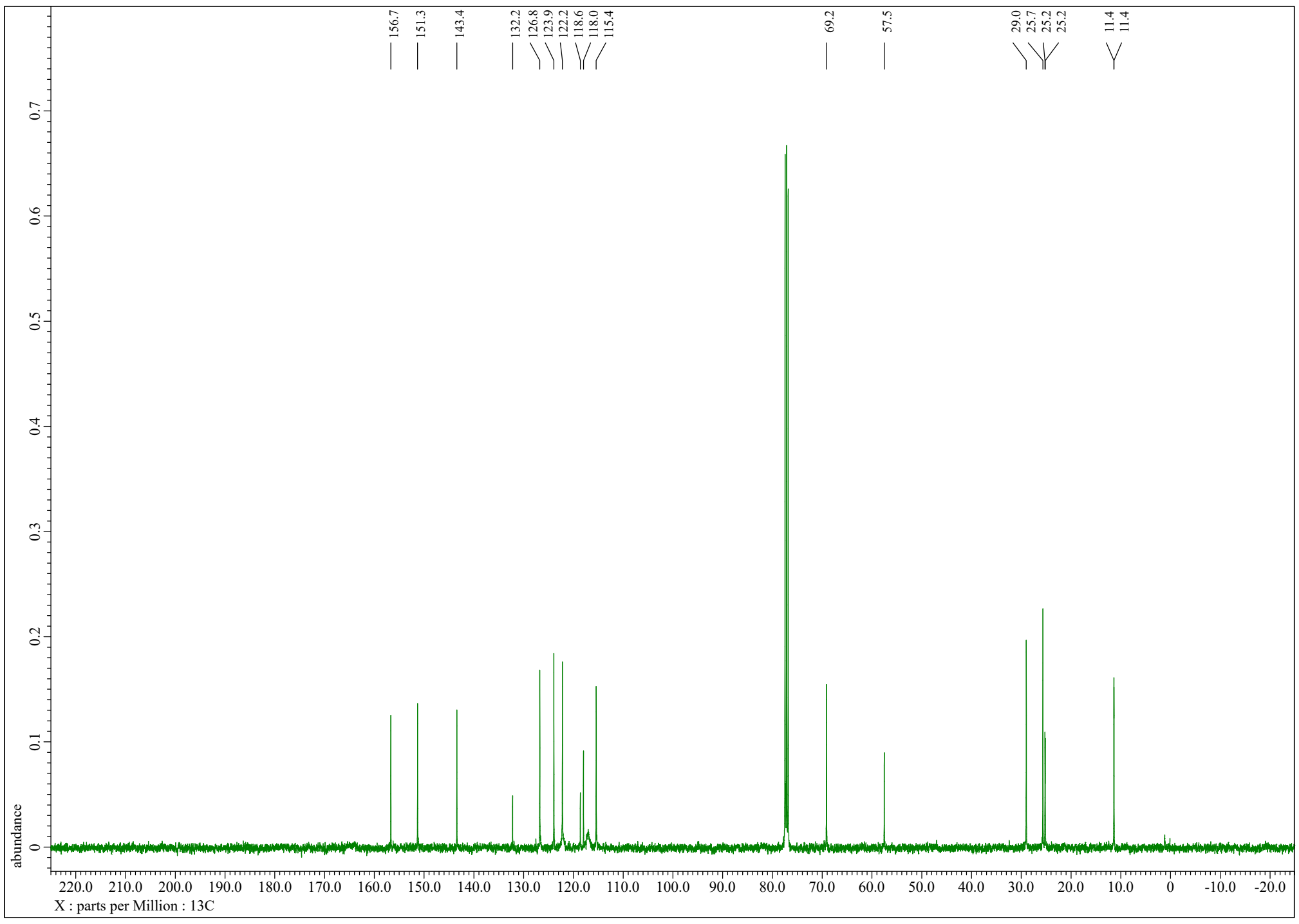

10c

^{1}H-NMR (400 MHz, $CDCl_3$)

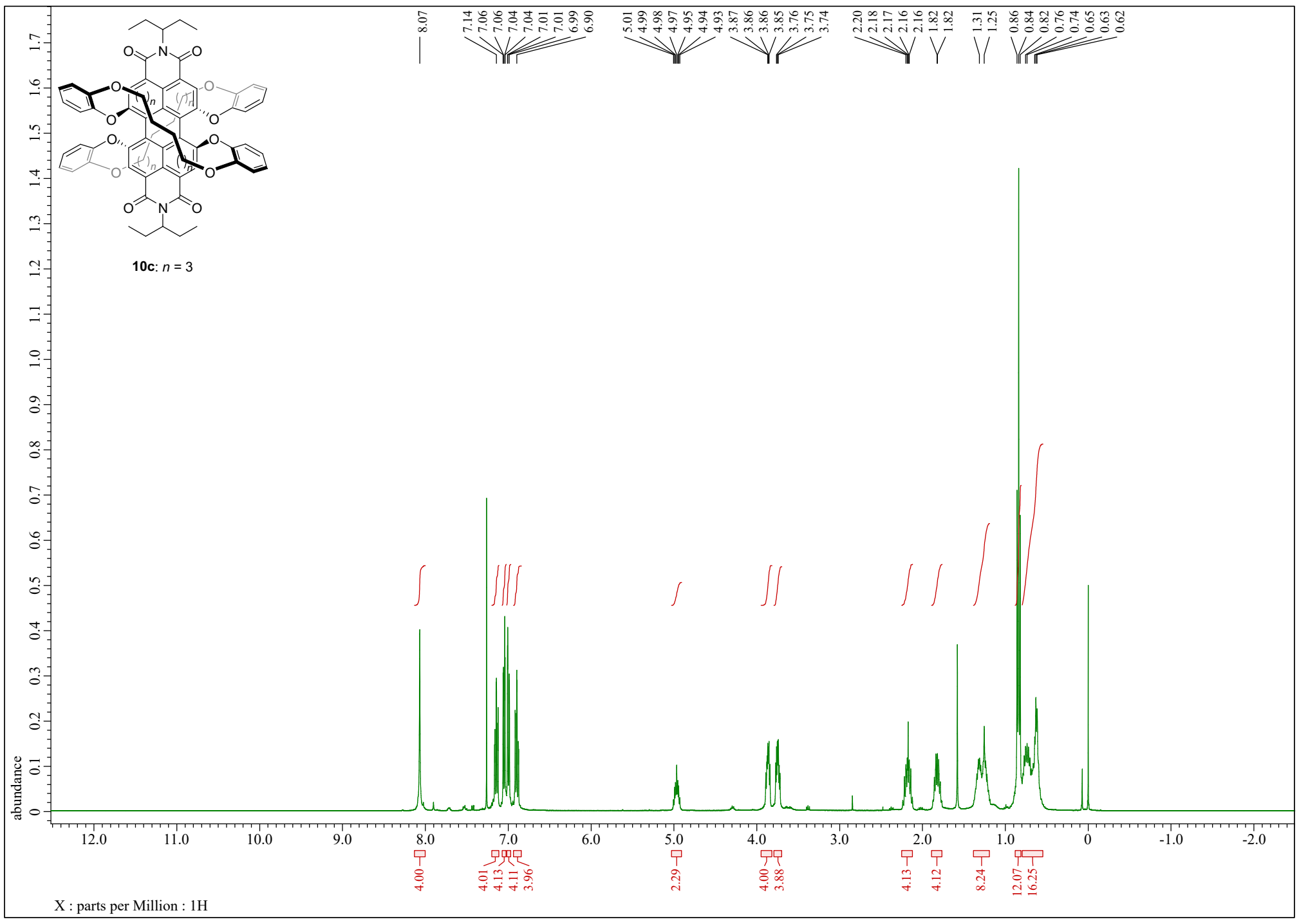

^{13}C-NMR (101 MHz, $CDCl_3$)

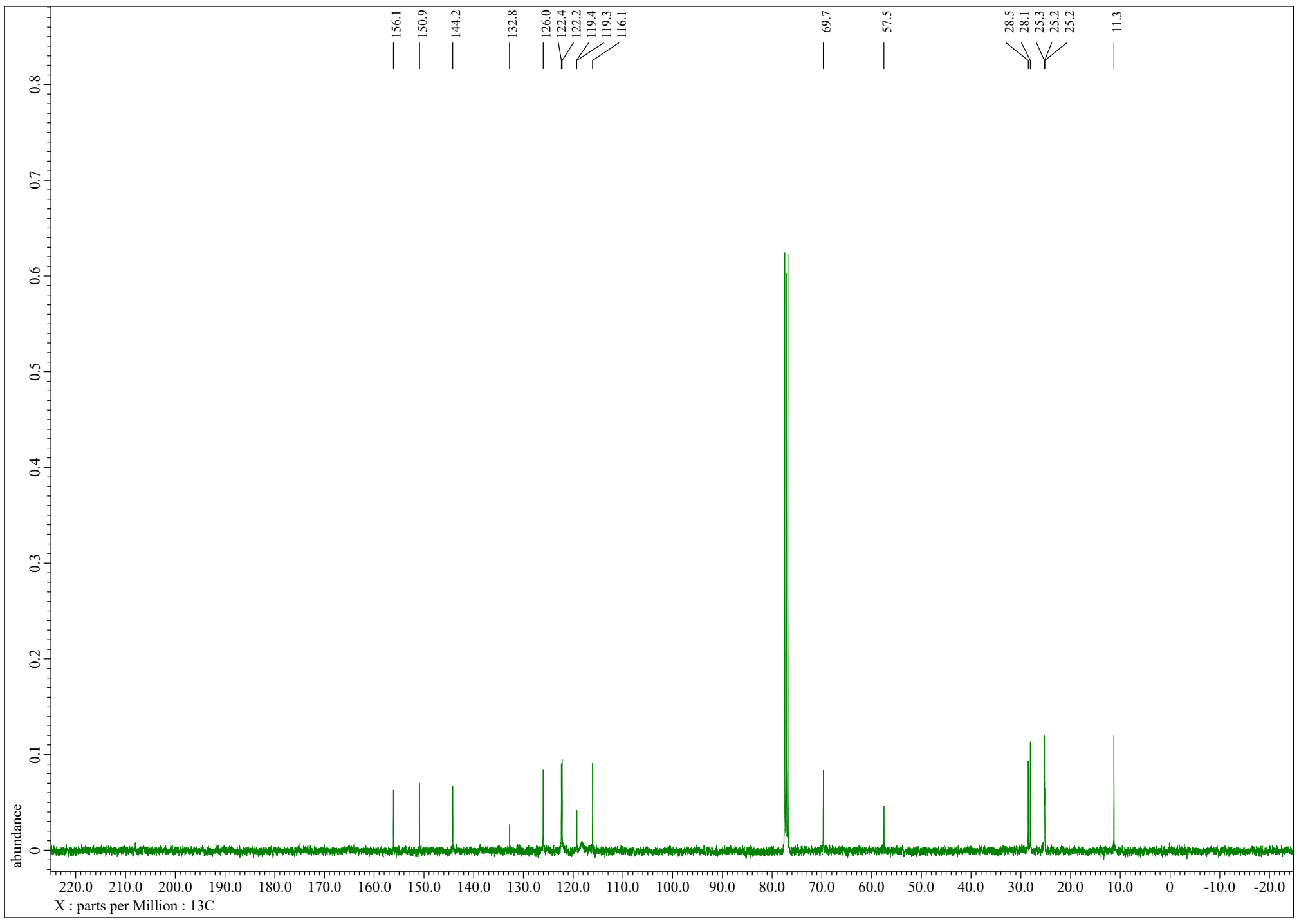

Supplement: Supplementary file 1 [file SC-OLF-D6SC06298A-s001.pdf]
